# Supplementary material for: Limitations to sustainable renewable jet fuels production attributed to cost than energy-water-food resource availability
Source: Nat Commun. 2023 Dec 9;14:8156. doi: 10.1038/s41467-023-44049-6 (PMC10710432; doi:10.1038/s41467-023-44049-6)
Supplement: Supplementary file 1 — Supplementary information [file 41467_2023_44049_MOESM1_ESM.pdf]

Supplementary Information

Limitations to sustainable renewable jet fuels production attributed to cost than energy-water-food resource availability

Cheng Tung Chong[a,*], Jo-Han Ng[b,*]

[a] China-UK Low Carbon College, Shanghai Jiao Tong University, Lingang, Shanghai 201306, China.

[b] Carbon Neutrality Research Group, University of Southampton Malaysia, 79100 Iskandar Puteri, Johor, Malaysia.

Email: [a]ctchong@sjtu.edu.cn, [b]J.Ng@soton.ac.uk

Outline of the supplementary information

No.	Content	Page number
1	Supplementary Figure 1	1
2	Data sources	2
3	Assumptions	17
4	Calculations	18
5	Exact unit conversion	26
6	Natural Resource Economics (NRE)	27
7	Equations for the model	28
8	Limiting factor (Profitable potential)	120
9	Indexing	127

List of Supplementary Table

Supplementary Table No.	Content	Page number
1	Data sources	2
2	Constants used in the model	7
3	Economic models	15
4	Processing cost for different production pathways	17
5	Description of the abbreviation used in the model	18
6	Crude Oil Price vs. Blending Ratio (COP vs. BR)	120
7	Water Stress vs. Blending Ratio (WS vs. BR)	121
8	Herfindahl-Hirschman Index vs. Blending Ratio (HHI vs. BR)	123
9	Food Stress vs. Blending Ratio (FS vs. BR)	124
10	Feedstock Quantity vs. Blending Ratio (FQ vs. BR)	125
11	Summary of the limiting factor calculation	126

1. Supplementary Figure 1

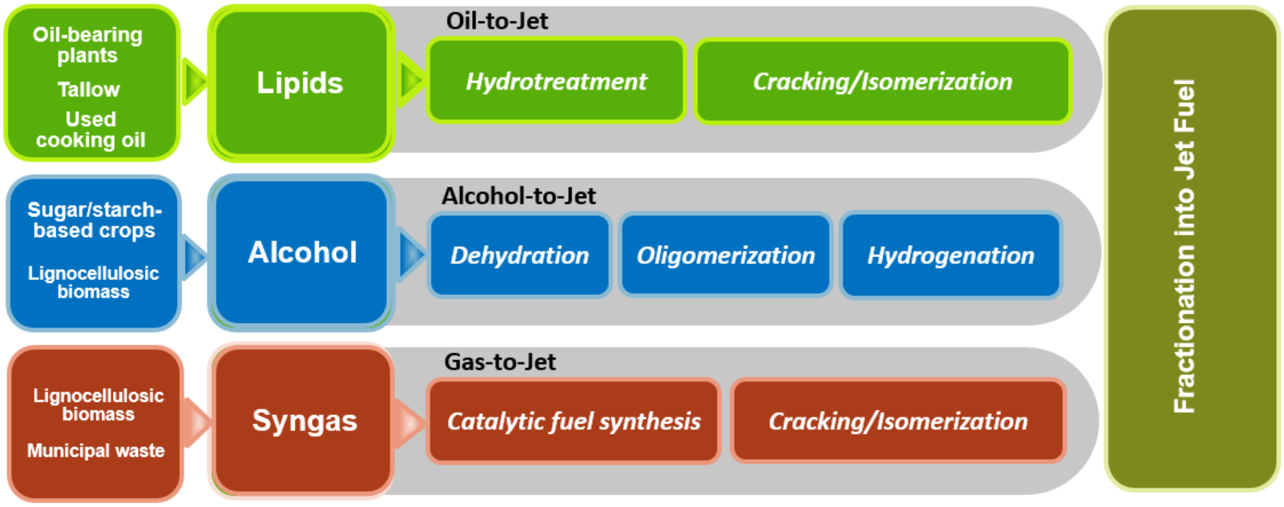

Supplementary Figure 1: RJF Conversion Pathways Methods. The three broad categories of biojet fuel production, namely oil-to-jet (OTJ), alcohol-to-jet (ATJ) and gas-to-jet (GTJ).

2. Data Sources

Supplementary Table 1: Data sources

Abbreviation	Name	Unit	Description	Timescale	Sources
CBP_c	Current Bio-Jet Fuel Production	1000 metric tonnes	Currrent bio-jet fuel production volume of each country	2011 – 2015	Source: UNdata & USDA (Additional)
EQ_{ac}	Export Quantity	metric tonnes	Quantity of each feedstock exported by each country	2016 – 2019	Source: FAOSTAT<br><br>39 feedstocks' selection: Inherited.<br>OTJ feedstocks: OTJ-EWF<br>ETJ feedstocks: E/GTJ-EWF (1st Gen)<br>GTJ feedstocks: E/GTJ-EWF (2nd Gen)
EV_{ac}	Export Value	1000 US$	Total value (US$) of trade export of each feedstock of each country	2016 – 2019	Source: FAOSTAT<br><br>39 feedstocks' selection: Inherited.<br>OTJ feedstocks: OTJ-EWF<br>ETJ feedstocks: E/GTJ-EWF (1st Gen)<br>GTJ feedstocks: E/GTJ-EWF (2nd Gen)
AH_{bc}	Area Harvested	ha	Total land area used for crop plantation of each country	2005 – 2019	Source: FAOSTAT<br><br>28 crops' selection: Inherited.<br>OTJ crops: OTJ-EWF<br>ETJ crops: E/GTJ-EWF (1st Gen)<br>GTJ crops: E/GTJ-EWF (2nd Gen)
JFC_c	Jet Fuel Consumption	1000 metric tonnes	Amount of jet fuel consumed by each country	1980 – 2020	Source: EIA
ES_c	Energy Sources / Primary Energy Balances	ktoe	Total primary energy sources of each country, including petroleum, primary energy, biofuels, coal, hydrocarbon gas liquids and natural gas and the corresponding refined products	2018 – 2019	Source: IEA<br><br>1. Coal<br>2. Crude oil<br>3. Oil products<br>4. Natural gas<br>5. Nuclear<br>6. Hydro<br>7. Wind,solar,etc.<br>8. Biofuels & Waste
$Population_c$	Population of the country	1000 pax	Population of each country	2019	Source: UN Population
JFI_c	Jet Fuel Import	1000 metric tonnes	Current jet fuel import quantity of each country	2016 – 2019	Source: Undata
TAL_c	Total Agricultural Land	1000 ha	Total agricultural land of a country for that year.<br><br>Agricultural area, this category is the sum of areas under FAO's	2016 – 2019	Source: FAOSTAT

			"Arable land", "Permanent crops" and "Permanent pastures".		
$CNEm_c$	Cropland Net Emissions	CO_2eq gigagrams	Net emissions in CO_2eq for all cropland of a country for that year.	2014 – 2017	Source: FAOSTAT
$AvgN_c$	Average Nitrogen N Use per Area of Cropland	kg/ha	Average nitrogen N used per cropland area.	2016 – 2019	Source: FAOSTAT
$SynNFEm_c$	Total Synthetic Nitrogen Fertilizers Emissions	CO_2eq gigagrams	Total emissions from the use of nitrogen fertilisers in the country for that year.	2014 – 2017	Source: FAOSTAT
AWW_c	Agricultural Water Withdrawal	10^9 m^3/year	Agricultural water withdrawal (10^9 m^3/year) of each country	2013 – 2017 (2017)	Source: AQUASTAT
$TRWR_c$	Total Renewable Water Resources	10^9 m^3/year	Total renewable water resources (10^9 m^3/year) of each country	2013 – 2017 (2017)	Source: AQUASTAT
$Precipitation_c$	Long-term Average Annual Precipitation in Volume	10^9 m^3/year	Long-term average annual precipitation in volume (10^9 m^3/year) of each country	2013 – 2017 (2017)	Source: AQUASTAT
$GNWF_{abc}$	Green Water Footprint	m^3/tonne	Water precipitation (rainwater) required per ton of crop / feedstock production for each country	1986 – 2016	Source: Zenodo (CWASI)
$BLWF_{abc}$	Blue Water Footprint	m^3/tonne	Irrigation water used per ton of crop / feedstock production for each country	1986 – 2016	Source: Zenodo (CWASI)
$GYWF_{abc}$	Grey Water Footprint	m^3/tonne	Fresh water required to dilute pollution caused per ton of crop / feedstock production for each country	1986 – 2016	Source: Zenodo (CWASI)
TWF_{abc}	Total Water Footprint	m^3/tonne	Total water required per ton of crop / feedstock production for each country	1986 – 2016	Source: Zenodo (CWASI)
CY_{bc}	Crop Yield	hg/ha	Yield of each feedstock of each country, depending on the feedstock type	2005 – 2019	Source: FAOSTAT<br><br>28 crops' selection: Inherited.<br>OTJ crops: OTJ-EWF<br>ETJ crops: E/GTJ-EWF (1st Gen)<br>GTJ crops: E/GTJ-EWF (2nd Gen)
HCY_b	Highest Crop Yield	hg/ha	Setting highest yield for every crop in the world. This is to get the highest crop production for every country by assuming that every country has the best technology for the crop production.	2005 – 2019	Source: FAOSTAT<br><br>28 crops' selection: Inherited.<br>OTJ crops: OTJ-EWF<br>ETJ crops: E/GTJ-EWF (1st Gen)<br>GTJ crops: E/GTJ-EWF (2nd Gen)

PQ_{bc}	Production Quantity	tonnes	Production quantity for each crop in each country	2005 – 2019	Source: FAOSTAT<br><br>28 crops' selection: Inherited.<br>OTJ crops: OTJ-EWF<br>ETJ crops: E/GTJ-EWF (1st Gen)<br>GTJ crops: E/GTJ-EWF (2nd Gen)
$VolCP_{bc}$	Crop Production Volatlity	%	A measure of the tendency for the production quantity of each crop to vary across 15 years in every country.	2005 – 2019	Source: FAOSTAT<br><br>28 crops' selection: Inherited.<br>OTJ crops: OTJ-EWF<br>ETJ crops: E/GTJ-EWF (1st Gen)<br>GTJ crops: E/GTJ-EWF (2nd Gen)<br><br>CALCULATION:<br><br>$\frac{\sigma_{PQ_{bc}}}{\mu_{PQ_{bc}}} \times 100\%$
$VolCPI_{bc}$	Intensified Crop Production Volatlity	%	A measure of the tendency for the intensified production quantity of each crop to vary across 15 years in every country.	2005 – 2019	Source: FAOSTAT<br><br>28 crops' selection: Inherited.<br>OTJ crops: OTJ-EWF<br>ETJ crops: E/GTJ-EWF (1st Gen)<br>GTJ crops: E/GTJ-EWF (2nd Gen)<br><br>CALCULATION:<br><br>$\frac{\sigma_{PQ\_I_{bc}}}{\mu_{PQ\_I_{bc}}} \times 100\%$
$ADESA_{c}$	Average Dietary Energy Supply Adequacy	%	Current percentage of Dietary Energy Supply of Average Dietary Energy Requirement, indicating the adequacy of food supply of the country in terms of calories	2017 (2016 – 2018) – 2020 (2019 – 2021)	Source: FAOSTAT
$ADER_{c}$	Average Dietary Energy Requirement	kcal/cap/day	Normative reference for adequate nutrition in the population of each country.<br><br>Average dietary energy requirement for each country is defined as the average caloric intake required to provide energy balance in the population (healthy weights for their genders, ages and activity levels).	2017 (2016 – 2018) – 2020 (2019 – 2021)	Source: FAOSTAT
$MDER_{c}$	Minimum Dietary Energy Requirement	kcal/cap/day	Minimum nutrition in the population for each country.<br><br>Minimum dietary energy requirement for	2017 (2016 – 2018) – 2020 (2019 – 2021)	Source: FAOSTAT

			each country is the average cut-off threshold caloric intake that the population would have to consume to attain their minimum acceptable weight for height, calculated based on the country's demographic weightings.		
DFD_c	Depth of Food Deficit	kcal/cap/day	Depth of food deficit for each country indicates how many calories would be needed to lift the undernourished from their status, everything else being constant.	2014 – 2016	Source: FAOSTAT
PU_c	Prevalence of Undernourishment	%	Prevalence of undernourishment shows the percentage of the population whose food intake is insufficient to meet dietary energy requirements continuously. Used MDER values.	2017 (2016 – 2018) – 2020 (2019 – 2021)	Source: FAOSTAT
PP_{bc}	Producer Prices	US$/tonnes	Producer prices in manufacturing measure prices of products (crops) sold as they leave the producer. They exclude any taxes, transport and trade margins that the purchaser may have to pay.	2005 – 2019	Source: FAOSTAT<br><br>28 crops' selection: Inherited.<br>OTJ crops: OTJ-EWF<br>ETJ crops: E/GTJ-EWF (1st Gen)<br>GTJ crops: E/GTJ-EWF (2nd Gen)
$VolPDP_{bc}$	Producer Price Volatility	%	A measure of the tendency for the producer price of each crop to vary across 15 years in each country.	2005 – 2019	Source: FAOSTAT<br><br>28 crops' selection: Inherited.<br>OTJ crops: OTJ-EWF<br>ETJ crops: E/GTJ-EWF (1st Gen)<br>GTJ crops: E/GTJ-EWF (2nd Gen)<br><br>CALCULATION:<br><br>$\frac{\sigma_{PP_{bc}}}{\mu_{PP_{bc}}} \times 100\%$
$VolPDPI_{bc}$	Intensified Producer Price Volatility	%	A measure of the tendency for the intensified producer price (defined as having intensified production quantity) of each crop to vary across 15 years in each country.	2005 – 2019	Source: FAOSTAT<br><br>28 crops' selection: Inherited.<br>OTJ crops: OTJ-EWF<br>ETJ crops: E/GTJ-EWF (1st Gen)<br>GTJ crops: E/GTJ-EWF (2nd Gen)<br><br>CALCULATION:

					$\frac{\sigma_{PP\_I_{bc}}}{\mu_{PP\_I_{bc}}} \times 100\%$
GI_c<br><br>1. VA_c<br>2. $PSAV_c$<br>3. GE_c<br>4. RQ_c<br>5. RL_c<br>6. CC_c	Governance Indicators	%	Governance indicators for each country (scaled by taking the maximum value as 100%)	1996 – 2019	Source: World Bank (Worldwide Governance Indicators)<br><br>1. Voice and Accountability<br>2. Political Stability and Absence of Violence<br>3. Government Effectiveness<br>4. Regulatory Quality<br>5. Rule of Law<br>6. Control of Corruption
BEV_c	Bio-jet Fuel Export	1000 metric tonnes	Export volume of pure bio-jet fuel for each country	2011 – 2019	Source: Eurostat
BIV_c	Bio-jet Fuel Import	1000 metric tonnes	Import volume of pure bio-jet fuel for each country	2011 – 2019	Source: Eurostat

Supplementary Table 2: Constants used in the model

Abbreviation	Name	Unit	Description	Value	Source
OY	OTJ Yield	-	Bio-jet fuel yield from OTJ process	0.79	Source: Journal Paper<br><br>[a1] The feasibility of short-term production strategies for renewable jet fuels – a comprehensive techno-economic comparison<br><br>CALCULATION:<br>a) Simple average of 0.75[a1] and 0.83 [a1] = 0.79
EY	ETJ Yield	-	Bio-jet fuel yield from ETJ process	0.56	Source: Journal Paper<br><br>[a1] The feasibility of short-term production strategies for renewable jet fuels – a comprehensive techno-economic comparison<br><br>CALCULATION:<br>Practical Yield, 0.56[90]
GY	GTJ Yield	-	Bio-jet fuel yield from GTJ process	0.38	Source: Journal Paper<br><br>[a2] IEA task 33, Aviation Biofuels through Biomass Gasification<br><br>CALCULATION:<br>$\frac{(108 \times 10^6) \times 0.788}{[(225 \times 1000) \times 1000]} = 0.38$<br><br>108 million litres of bio-jet fuel<br>225 kilotonnes of feedstocks<br>0.788 kg/L of bio-jet fuel density
THY(Sugar)	Theoretical Hydrolysis Yield of Sugar	-	Theoretical hydrolysis yield of sugar into glucose	1	Source: Inherited from E/GTJ-EWF<br><br>[b3] Enological Chemistry: Sugar in Must<br><br>1ST GEN:<br>a) Sugar Glucose per unit sugar = 1 [b3]
THY(Starch)	Theoretical Hydrolysis Yield of Starch	-	Theoretical hydrolysis yield of starch into glucose	1.111	Source: Inherited from E/GTJ-EWF<br><br>[b2] Assessment of ethanol production options for corn products<br><br>1ST GEN:<br>a) Starch Glucose per unit starch = 1.111 [b2]
HE(Sugar)	Hydrolysis Efficiency of Sugar Feedstock	-	Practical efficiency of hydrolysis process for conc. sulphuric acid, some sugar is degraded	1	Source: Inherited from E/GTJ-EWF<br><br>[b1] Bioethanol from Cellulosic Materials: A Renewable Motor Fuel from Biomass

					NOTE:<br><br>Since simple sugars are converted directly into bioethanol, hydrolysis process is not required for simple sugar (e.g glucose, fructose), so sugar to glucose hydrolysis is put as one
HE(Starch)	Hydrolysis Efficiency of Starch Feedstock	-	Practical efficiency of hydrolysis process for conc. sulphuric acid, some sugar is degraded	0.95	Source: Journal Paper<br><br>[b6] Process for enzymatic hydrolysis of starch to glucose<br><br>NOTE:<br><br>D-glucose yields are limited to approximately 95% in concentrated starch solutions.
FY	Fermentation Yield	-	Fermentation theoretical yield	0.511	Source: Inherited from E/GTJ-EWF<br><br>[b1] Bioethanol from Cellulosic Materials: A Renewable Motor Fuel from Biomass<br><br>NOTE:<br><br>- For Glucose: $C_6H_{12}O_6$ à $2C_2H_5OH$ + $2CO_2$<br>-100 grams of glucose produces 51.4g bioethanol and 48.8g carbon dioxide
FE	Fermentation Efficiency	-	Practical efficiency of fermentation process Due to formation of by-products such as microorganisms and other chemical products.<br>Sugar is used up for the cellular matter of yeast cell.	0.92	Source: Inherited from E/GTJ-EWF<br><br>[b5] Ethanol fuel from biomass: Review
SEE	Starch to Ethanol Efficiency	-	Overall efficiency of recovering ethanol from starch	0.95	Source: Inherited from E/GTJ-EWF<br><br>[b2] Assessment of ethanol production options for corn products
OD_{ab}	Oil Density	kg/L	Oil density of different feedstocks	-	Source: Journal Papers<br><br>[d4] Physical Properties of fats and Oils<br>[d6] Firestone, David-Physical and Chemical Characteristics of Oils, Fats, and Waxes (3rd Edition)-AOCS Press (2013)<br>[d8] ManualdeLange_9164

					[d9] Evaluation of rice bran, sesame and moringa oils as feasible sources of biodiesel and the effect of blending on their physicochemical properties<br><br>NOTE:<br><br>All oil density is taken at 15°C.
BD	Bio-Jet Fuel Density	kg/L	Density of bio-jet fuel	0.788	Source: Journal Paper<br><br>[h1] Techno-economic review of alternative fuels and propulsion systems for the aviation sector<br><br>NOTE:<br><br>Bio-jet fuel density is taken at 15°C.
FEC_{ab}	Feedstock Energy Content	MJ/kg	Energy content of each feedstock	-	Source: Journal Papers<br><br>For OTJ feedstocks:<br><br>[d2] Emission_kapok oxygen<br>[d10] Emission_Oxygen Content of Cotton Seed<br>[d11] Energy_FEC safflower<br>[d12] Energy_FEC palm kernel<br>[d13]Energy_FEC linseed rice bran<br><br>For ETJ feedstocks:<br><br>[e1] Feedstock Energy Content for Sugar Beet<br>[e2] Feedstock Energy Content Oats<br>[e3] ETJ feedstock energy content (Excel File)<br>[e4] Feedstock Energy Content Watermelon<br>[e5] Feedstock Energy Content Maizes<br>[e6] Feedstock Energy Content Barley<br>[e7] Feedstock Energy Content Sweet Potatoes<br>[e8] Feedstock Energy Content Banana<br>[e9] Feedstock Energy Cassava<br>[e10] Feedstock Energy Content Dates
OC_{ab}	Oil Content	wt%	Oil content of feedstock	-	Source: Journal Papers<br><br>[d1] A comprehensive review oh biodiesel as an alternative energy resource and its characteristics<br>[d2] Emission_kapok oxygen<br>[d3] Energy_FEC safflower

					[d5] Properties of various platns and animals feedstocks for biodiesel production<br>[d7] Oil Content and Composition of the Seed in the World Collection of Sesame Introductions
BEC	Bio-Jet Fuel Energy Content	MJ/kg	Energy content (lower heating value) of bio-jet fuel	43.90	Source: Journal Paper<br><br>[h1] Techno-economic review of alternative fuels and propulsion systems for the aviation sector
JEC	Jet Fuel Energy Content	MJ/kg	Energy content (lower heating value) of jet fuel	43.02	Source: Journal Paper<br><br>[h1] Techno-economic review of alternative fuels and propulsion systems for the aviation sector
BEI_{NOx}	Bio-Jet Fuel (NO_x) Emission Index	g_{NOx}/kg_{fuel}	NO_x emission index of bio-jet fuel	4.860	Source: ACRP<br><br>[f4] acrp_wod_41
BEI_{CO}	Bio-Jet Fuel (CO) Emission Index	g_{CO}/kg_{fuel}	CO emission index of bio-jet fuel	35.10	Source: ACRP<br><br>[f4] acrp_wod_41
BEI_{UHC}	Bio-Jet Fuel (UHC) Emission Index	g_{UHC}/kg_{fuel}	UHC emission index of bio-jet fuel	3.655	Source: SWAFEA, EC<br><br>[f2] SW_WP6_D.6.2_Environmental analysis_Onera_28Mar2011
BEI_{Soot}	Bio-Jet Fuel (Soot) Emission Index	g_{soot}/kg_{fuel}	Soot emission index of bio-jet fuel	0.001992	Source: ACRP<br><br>[f4] acrp_wod_41
$JFEI_{NOx}$	Jet Fuel (NO_x) Emission Index	g_{NOx}/kg_{fuel}	NO_x emission index of jet fuel	5.211	Source: EEDB, ICAO<br><br>[f1] Emission Databank for different Engines (Excel File)
$JFEI_{CO}$	Jet Fuel (CO) Emission Index	g_{CO}/kg_{fuel}	CO emission index of jet fuel	34.86	Source: EEDB, ICAO<br><br>[f1] Emission Databank for different Engines (Excel File)
$JFEI_{UHC}$	Jet Fuel (UHC) Emission Index	g_{UHC}/kg_{fuel}	UHC emission index of jet fuel	3.047	Source: EEDB, ICAO<br><br>[f1] Emission Databank for different Engines (Excel File)
$JFEI_{Soot}$	Jet Fuel (Soot) Emission Index	g_{soot}/kg_{fuel}	Soot emission index of jet fuel	0.003065	Source: EEDB, ICAO<br><br>[f1] Emission Databank for different Engines (Excel File)
JD	Jet Fuel Density	kg/L	Density of jet fuel	0.82	Source: Journal Paper

					[h1] Techno-economic review of alternative fuels and propulsion systems for the aviation sector<br><br>NOTE:<br><br>Jet fuel density is taken at 15°C.
MBR	Maximum Blending Ratio	%	Maximum allowable blending ratio by the regulation (ASTMD7566 – 19).	50	Source: IATA<br><br>[g1] Fact Sheet 2 Sustainable Aviation Fuel Technical Certification<br><br>NOTE:<br><br>Current regulated maximum blending ratio for Oil-to-Jet (HEFA), Ethanol-to-Jet (ATJ), Gas-to-Jet (FT process) is 50%.
WR(O)	Water Required (OTJ)	$L/L_{bio\text{-}jet}$	Water required by bio-jet fuel production through OTJ process	1.3977	Source: Journal Paper<br><br>[i1] A Techno-Economic and Environmental Assessment of Hydroprocessed Renewable Distillate Fuels<br><br>CALCULATION:<br><br>$\frac{1.4 \times 0.788}{0.79 \times 0.999099} = 1.3977$<br><br>1.4 pounds of water per pound of vegetable oil processed<br>OTJ yield = 0.79<br>Water density at 15°C = 0.999099<br>Bio-jet fuel density = 0.788<br><br>1.3977 litre of water per litre of bio-jet fuel produced<br><br>NOTE:<br><br>Only consider water used for bio-jet fuel production from OTJ feedstocks.
WR(E)	Water Required (ETJ)	$L/L_{bio\text{-}jet}$	Water required by bio-jet fuel production through ETJ process	8.5	Source: Journal Paper<br><br>[i2] Well-to-wake analysis of ethanol-to-jet and sugar-to-jet production pathway<br><br>NOTE:<br><br>Only consider water used for bio-jet fuel production from ETJ feedstocks.
WR(G)	Water Required (GTJ)	$L/L_{Bio\text{-}jet}$	Water required by bio-jet fuel production through GTJ process	4.5	Source: Journal Paper

					[i3] Water Footprint and Land Requirement of Solar Thermochemical Jet-Fuel Production<br><br>Taking the mid-point value of 2-7 $L/L_{bio\text{-}jet\ fuel}$<br><br>NOTE:<br><br>Only consider water used for bio-jet fuel production from GTJ feedstocks.
DMP_{ab}	Dry Matter Percentage	%	Dry matter percentage for 1st and 2nd generation sugar feedstock	-	Source: Journal Papers<br><br>For ETJ feedstocks:<br><br>[e11] Global potential bioethanol production from wasted crops and crop residues<br>[e12] Fruit quality of mini watermelon<br>[e13] Bioethanol production from tuber crops using fermentation technology a review<br>[e14] Banana biomass as potential renewable energy source<br>[e15] Effect of storage duration on some physical properties of date palm<br><br>For GTJ feedstocks:<br><br>[e11] Global potential bioethanol production from wasted crops and crop residues<br>[e16] Bioethanol from lignocellulose Status perspectives and challenges in Malaysia<br>[e17] Oil palm fibers_ Morphology, chemical composition, surface modification, and mechanical properties
SSC_{ab}	Starch or Sugar content	%	Starch or sugar content is the amount of starch or sugar present in feedstock's dry matter according to the type of feedstock	-	Source: Journal Papers<br><br>[e12] Fruit quality of mini watermelon<br>[e18] Ethanol production from date wastes adapted technologies, challenges and global potential<br>[e19] Bioethanol production from renewable sources current perspective and technological progress<br>[e20] What crop rotation will provide optimal first Ireland<br>[e21] Roots, tubers, plantains and bananas in animal feeding<br>[e22] An evaluation of cassava, sweet potato and field corn as

					potential carbohydrate sources for bioethanol production in Alabama and Maryland
WR_{ab}	Waste Ratio	-	Percentage of wastes from dry matter of GTJ (2nd generation) feedstock	-	Source: Journal Papers<br><br>[e11] Global potential bioethanol production from wasted crops and crop residues<br>[j1] Availability, use, and removal of oil palm biomass in Indonesia
WCR	Waste Collectible Ratio	-	Percentage of collectible agricultural waste per unit of total amount of agriculture waste (2nd generation)	0.35	Source: Inherited from E/GTJ-EWF<br><br>[j2] A Geographic Perspective on the Current Biomass Resource Availability in the United States
FC_a	Feedstock Cost	US$/tonne	Cost of feedstock per tonne for every 2nd generation feedstock, from producer perspective.	-	Source: Journal Papers & Reports<br><br>[k1] Corn stover for bioenergy production Cost<br>[k2] Barley and Wheat<br>[k3] Kulim IAR 2016 Sec4 Plantation<br>[k4] National Biomass Strategy 2020 New wealth creation for Malaysia's biomass industry<br>[k5] Oat straw<br>[k7] Cardosoetal.2013
PTC	Pretreatment Cost	US$/dry tonne feed	Drying cost per fry tonne of 2nd generation feedstock for GTJ (gasification) process	0.62	Source: Journal Paper<br><br>[j3] Biomass Pre-treatment for bioenergy<br><br>NOTE:<br><br>Only consider drying cost, size reduction cost of 2.78 and palletization of 4.50 are not considered.
GD	Glucose Density	kg/L	Glucose Density	1.562	Source: NCBI<br><br>[h2] D(+)-Glucose _ C6H12O6 – PubChem<br><br>NOTE:<br><br>Glucose Density is taken at 18°C.
ED	Bioethanol Density	kg/L	Bioethanol density	0.79	Source: Journal Paper<br><br>[h3] Diesel Biodiesel Gasoline Bioethanol Density<br><br>NOTE:<br><br>Glucose Density is taken at 15°C.

WFF_{ab}	Water Footprint Factors	-	Factors used to multiply with the water footprints of the main/root crops to determine the green, blue and grey water footprint of the processed/derived crops (feedstocks) in each country	-	Source: Journal Paper & Report<br><br>[m1] The Green, Blue and Grey Water Footprint of Crops and Derived Crop Products<br>[m2] uWFs_primary&derived_crops<br>[m2-1] country_list (Excel File)<br>[m2-2] item_list (Excel File)<br>[m3] Virtual water trade and water footprint of agricultural goods (1961-2016)

Supplementary Table 3: Economic models

Abbreviation	Name	Unit	Description	Value	Source
$PC(O)_d$	Processing Cost (OTJ)	$US\$_{2013}/L$	Processing cost per litre of pure bio-jet fuel produced through OTJ process (in 2013 USD)	0.4189, -0.2415, -0.6378	Source: Journal Paper<br><br>[a1] The feasibility of short-term production strategies for renewable jet fuels – a comprehensive techno-economic comparison<br><br>CALCULATION:<br><br>$\frac{(MFSP - Feedstock) \times 0.788}{1000 \times Conversion\ Rate}$<br><br>Minimum Jet Fuel Selling Price and Feedstock Cost (Used Cooking Oils) are determined using Plot Digitizer Online from the graph in [a1].<br>Bio-jet fuel density = 0.788<br>Conversion from USD_{2013} to $Euro_{2013}$ = 0.7528 (Geometric mean of annual conversion)<br><br>*Lowest PC is under full subsidy condition (Federal + Market Price subsidy)<br>*Middle PC value is under federal subsidy condition only<br>*Highest PC value is under without subsidy condition
$PC(E)_d$	Processing Cost (ETJ)	$US\$_{2013}/L$	Processing cost per litre of pure bio-jet fuel produced through ETJ process (in 2013 USD)	1.69, 1.0296, 0.6334	Source: Journal Paper<br><br>[a1] The feasibility of short-term production strategies for renewable jet fuels – a comprehensive techno-economic comparison<br><br>CALCULATION:<br><br>$\frac{(MFSP - Feedstock) \times 0.788}{1000 \times Conversion\ Rate}$<br><br>Minimum Jet Fuel Selling Price and Feedstock Cost (Forest Residues, Wheat Straws) are determined using Plot Digitizer Online from the graph in [a1].<br>Bio-jet fuel density = 0.788<br>Conversion from USD_{2013} to $Euro_{2013}$ = 0.7528 (Geometric mean of annual conversion)<br><br>*Lowest PC is under full subsidy condition (Federal + Market Price subsidy)<br>*Middle PC value is under federal subsidy condition only<br>*Highest PC value is under without subsidy condition

PC(G)$_d$	Processing Cost (GTJ)	US$_{2013}$/L	Processing cost per litre of pure bio-jet fuel produced through GTJ process (in 2013 USD)	1.1411, 0.2139, -0.1824	Source: Journal Paper<br><br>[a1] The feasibility of short-term production strategies for renewable jet fuels – a comprehensive techno-economic comparison<br><br>CALCULATION:<br><br>$\frac{(MFSP - Feedstock) \times 0.788}{1000 \times Conversion\ Rate}$<br><br>Minimum Jet Fuel Selling Price and Feedstock Cost (Used Cooking Oils) are determined using Plot Digitizer Online from the graph in [a1].<br>Bio-jet fuel density = 0.788<br>Conversion from USD$_{2013}$ to Euro$_{2013}$ = 0.7528 (Geometric mean of annual conversion)<br><br>*Lowest PC is under full subsidy condition (Federal + Market Price subsidy)<br>*Middle PC value is under federal subsidy condition only<br>*Highest PC value is under without subsidy condition
MFSP(O)	Minimum Fuel Selling Price (OTJ)	US$_{2013}$/L	Minimum fuel selling price per litre of pure bio-jet fuel produced through OTJ process (in 2013 USD)	1.3578	Source: Journal Paper<br><br>[a1] The feasibility of short-term production strategies for renewable jet fuels – a comprehensive techno-economic comparison
MFSP(E)	Minimum Fuel Selling Price (ETJ)	US$_{2013}$/L	Minimum fuel selling price per litre of pure bio-jet fuel produced through ETJ process (in 2013 USD)	2.9901	Source: Journal Paper<br><br>[a1] The feasibility of short-term production strategies for renewable jet fuels – a comprehensive techno-economic comparison
MFSP(G)	Minimum Fuel Selling Price (GTJ)	US$_{2013}$/L	Minimum fuel selling price per litre of pure bio-jet fuel produced through GTJ process (in 2013 USD)	2.1451	Source: Journal Paper<br><br>[a1] The feasibility of short-term production strategies for renewable jet fuels – a comprehensive techno-economic comparison

3. Assumptions

1. Processing cost (PC) as three-value variable
a) Without government (federal) subsidies
b) With government (federal) subsidies
c) With subsidy and D5/D4 RIN (stacking).

Supplementary Table 4: Processing cost for different production pathways

**Production Pathways**	**PC w/o Subsidies** (US$$_{2013}$/L)	**PC w/ Subsidies** (US$$_{2013}$/L)	**PC w/ Stacking** (US$$_{2013}$/L)
OTJ	0.405076110373821	-0.255354020521550	-0.651612099058773
ETJ	1.634277697593740	0.973847566698371	0.577589488161148
GTJ	1.103487420367910	0.176243516590808	-0.220014561946415

- Units: US$$_{2013}$/L

2. The highest production quantity amongst all countries (according to crop/feedstock types) to be quantity used for bio-jet fuel production for all countries in intensified scenario.
- All countries have the same (and the best) harvesting technology to produce the largest crop quantities.
- The highest crop yield achieved by one country is used for all other countries.

3. Production quantity for each country (according to crop/feedstock types) to be quantity used for bio-jet fuel production in harvested scenario.
- All the crops produced in every country is used to produce bio-jet fuel.

4. Export quantity to be quantity used for bio-jet fuel production in potential and profitable potential scenarios.
- Domestic sale of feedstocks and feedstock products are assumed to have other uses and cannot be put into further bio-jet fuel production (BP) under OTJ, ETJ and GTJ process.
- Exported feedstocks and feedstock products are assumed to be surplus that can be used in bio-jet fuel production without affecting other living necessities such as food.
- Threshold values: 10 tonnes per day (OTJ) and 100 tonnes per day (ETJ and GTJ)
    - Only feedstocks with export quantity higher than threshold values are taken for bio-jet fuel production.
    - This is to ensure a large-scale production.

5. Jet fuel price
- Jet fuel price is assumed to vary linearly with crude oil price.
    - Jet fuel is extracted from crude oil.
        - The extraction process used energy mainly from crude oil.
        - Extraction costs vary linearly with crude oil price.
- Average of jet fuel price to crude oil price ratio is taken for the last twenty years.

6. Bio-jet fuel is sold at a price ≤ jet fuel price
- Bio-jet fuel price ≤ jet fuel price so that bio-jet fuel can attract customers and investors.
- Bio-jet fuel is meant to replace jet fuel; therefore, its price is compared to jet fuel price.

7. All nitrogen use in agricultural soils comes from synthetic nitrogen fertilisers only.

4. Calculations

Supplementary Table 5: Description of the abbreviation used in the model

Abbreviation	Name	Unit	Description
1. Oil-to-Jet (OTJ)			
$FT(O)_{a}$	Feedstock Threshold (OTJ)	kg	Minimum feedstock quantity to produce bio-jet fuel for every feedstock in each country through OTJ process.
$PQ(O)\_I_{bc}$	Production Quantity (OTJ) _ Intensified	kg	The maximum crop produced for every country to produce bio-jet fuel under OTJ process.
$CC(O)_{abc}$	Crop Consumption (OTJ)	kg	Current crop consumption by every country for the crops being used for OTJ process.
LQ_NRE_{abc}	Lipid Quantity _ Natural Resource Economics	kg	Amount of lipid contained in each OTJ feedstock in every country under different NRE scenarios.
ULQ_P_{abc}	Usable Lipid Quantity _ Potential	kg	Amount of lipid of OTJ feedstock that can be used to produce bio-jet fuel (actual usable mass that will be used to produce bio-jet fuel in every country).<br><br>CONDITION:<br><br>Lipid quantity of a particular feedstock must be greater than the feedstock threshold (to ensure large scale utilisation of bio-jet fuel).
LEV_PrP_{abc}	Lipid Export Value _ Profitable Potential	US$/kg	Lipid exported price for every OTJ feedstock in every country.
$UBEV(O)\_PrP_{abcde}$	Upper Boundary Export Value (OTJ) _ Profitable Potential	US$/kg	The highest cost that can be spent per kilogram of lipid in feedstock for bio-jet fuel production to be profitable through OTJ process under different COP / JFP and PC condition.
BP(O)_NRE	Bio-Jet Fuel Production (OTJ) _ Natural Resource Economics	L	Bio-jet fuel production volume from OTJ process under different NRE scenarios.
NAF(O)_NRE	Number of Available Feedstock (OTJ) _ Natural Resource Economics	-	Number of available OTJ feedstocks available to produce bio-jet fuel under different NRE scenarios.<br><br>NOTE:<br><br>Maximum number of available OTJ feedstocks is 16.
$CDF(O)_{ac}$	Current Dominant Feedstock (OTJ)	-	OTJ feedstock with the highest bio-jet fuel production for each country.
$DPA(O)_{abc}$	Domestic Plantation Area (OTJ)	ha	Current OTJ domestic feedstock plantation area for each country (with OTJ dominant feedstock).
PA(O)_NRE	Plantation Area (OTJ) _ Natural Resource Economics	ha	Plantation area required to produce bio-jet fuel under different NRE scenarios for each OTJ feedstock in each country.
CWR(O)_NRE	Crop Water Required (OTJ) _ Natural Resource Economics	m^3	Water required to produce each OTJ feedstock for bio-jet fuel production in each country under different NRE scenarios.
WRP(O)_NRE	Water Required for Production (OTJ) _ Natural Resource Economics	L	Total amount of water required for OTJ process to produce bio-jet fuel for each country under different NRE scenarios.
EF(O)_NRE	Energy of Feedstock (OTJ) _ Natural Resource Economics	MJ	Energy of each OTJ feedstock used by each country under different NRE scenarios.
$CY(O)\_PrP_{bcde}$	Crop Yield (OTJ) _ Profitable Potential	hg/ha	Quantity produced per hectare of plantation area for each OTJ crops for each country under different COP / JFP and PC condition.

VolCP(O)_PrP$_{bcde}$	Volatility of Crop Production (OTJ) _ Profitable Potential	%	A measure of the tendency for the production quantity of OTJ crop to vary across 15 years for each country under different COP / JFP and PC condition.
VolPDP(O)_PrP$_{bcde}$	Producer Price Volatility (OTJ) _ Profitable Potential	%	A measure of the tendency for the producer price of OTJ crop to vary across 15 years for each country under different COP / JFP and PC condition.
2. Ethanol-to-Jet (ETJ)			
SCR$_{CT}$	Sugar to Composition Ratio	-	Conversion ratio from sugar/starch to glucose.
BY$_{CT}$	Bioethanol Yield	-	Conversion ratio from sugar/starch to bioethanol.
FT(E)$_{a}$	Feedstock Threshold (ETJ)	kg	Minimum feedstock quantity to produce bio-jet fuel for every feedstock in each country through ETJ process.
PQ(E)_I$_{bc}$	Production Quantity (ETJ) _ Intensified	kg	The maximum crop produced for every country to produce bio-jet fuel under ETJ process.
CC(E)$_{abc}$	Crop Consumption (ETJ)	kg	Current crop consumption by every country for the crops being used for ETJ process.
DMQ(E)_NRE$_{abc}$	Dry Matter Quantity (ETJ) _ Natural Resource Economics	kg	Amount of dry matter contained in each ETJ feedstock under different NRE scenarios.
UDMQ(E)_P$_{abc}$	Usable Dry Matter Quantity (ETJ) _ Potential	kg	Amount of dry matter from ETJ feedstock that can be used to produce bio-jet fuel (actual usable mass that will be used to produce bio-jet fuel in every country).<br><br>CONDITION:<br><br>Dry matter quantity of a particular feedstock must be greater than the feedstock threshold (to ensure large scale utilisation of bio-jet fuel).
SQ_PrP$_{abc}$	Sugar Quantity _ Profitable Potential	kg	Quantity of the sugar content from each ETJ feedstock in every country.
SEV_PrP$_{abc}$	Sugar Export Value _ Profitable Potential	US$/kg	Sugar exported price for each ETJ feedstock in every country.
UBEV(E)_PrP$_{abcde}$	Upper Boundary Export Value (ETJ) _ Profitable Potential	US$/kg	The highest cost that can be spent per kilogram of dry matter in feedstock for bio-jet fuel production to be profitable through ETJ process under different COP / JFP and PC condition.
BP(E)_NRE	Bio-Jet Fuel Production (ETJ) _ Natural Resource Economics	L	Bio-jet fuel production volume from ETJ process under different NRE scenarios.
NAF(E)_NRE	Number of Available Feedstock (ETJ) _ Natural Resource Economics	-	Number of available ETJ feedstocks available to produce bio-jet fuel under different NRE scenarios.<br><br>NOTE:<br><br>Maximum number of available ETJ feedstocks is 12.
CDF(E)$_{ac}$	Current Dominant Feedstock (ETJ)	-	ETJ feedstock with the highest bio-jet fuel production for each country.
DPA(E)$_{abc}$	Domestic Plantation Area (ETJ)	ha	Current ETJ domestic feedstock plantation area for each country (with ETJ dominant feedstock).
PA(E)_NRE	Plantation Area (ETJ) _ Natural Resource Economics	ha	Plantation area required to produce bio-jet fuel under different NRE scenarios for each ETJ feedstock in each country.
CWR(E)_NRE	Crop Water Required (ETJ) _ Natural Resource Economics	m^3	Water required to produce each ETJ feedstock for bio-jet fuel production in each country under different NRE scenarios.
WRP(E)_NRE	Water Required for Production (ETJ) _ Natural Resource Economics	L	Total amount of water required for ETJ process to produce bio-jet fuel for each country under different NRE scenarios.
EF(E)_NRE	Energy of Feedstock (ETJ) _ Natural Resource Economics	MJ	Energy of each ETJ feedstock used by each country under different NRE scenarios.

CY(E)_PrP$_{bcde}$	Crop Yield (ETJ) _ Profitable Potential	hg/ha	Quantity produced per hectare of plantation area for each ETJ crops for each country under different COP / JFP and PC condition.
VolCP(E)_PrP$_{bcde}$	Volatility of Crop Production (ETJ) _ Profitable Potential	%	A measure of the tendency for the production quantity of ETJ crop to vary across 15 years for each country under different COP / JFP and PC condition.
VolPDP(E)_PrP$_{bcde}$	Producer Price Volatility (ETJ) _ Profitable Potential	%	A measure of the tendency for the producer price of ETJ crop to vary across 15 years for each country under different COP / JFP and PC condition.
3. Gas-to-Jet (GTJ)			
FT(G)$_{a}$	Feedstock Threshold (GTJ)	kg	Minimum feedstock quantity to produce bio-jet fuel for every feedstock in each country through GTJ process.
PQ(G)_I$_{bc}$	Production Quantity (GTJ) _ Intensified	kg	The maximum crop produced for every country to produce bio-jet fuel under GTJ process.
DMQ(G)_NRE$_{abc}$	Dry Matter Quantity (GTJ) _ Natural Resource Economics	kg	Amount of 2nd generation dry matter that can be obtained from agricultural waste for GTJ process under different NRE scenarios.
UDMQ(G)_P$_{abc}$	Usable Dry Matter Quantity (GTJ) _ Potential	kg	Amount of 2nd generation dry matter from GTJ wastes that can be used to produce bio-jet fuel (actual usable mass that will be used to produce bio-jet fuel in every country).<br><br>CONDITION:<br><br>Dry matter quantity of a particular feedstock must be greater than the feedstock threshold (to ensure large scale utilisation of bio-jet fuel).
DMV_PrP$_{a}$	Dry Matter Value _ Profitable Potential	US$/kg	2nd generation dry matter quantity price for each country.
UBEV(G)_PrP$_{abcde}$	Upper Boundary Export Value (GTJ) _ Profitable Potential	US$/kg	The highest cost that can be spent per kilogram of dry matter in feedstock for bio-jet fuel production to be profitable through GTJ process under different COP / JFP and PC condition.
BP(G)_NRE	Bio-Jet Fuel Production (GTJ) _ Natural Resource Economics	L	Bio-jet fuel production volume from GTJ process under different NRE scenarios.
NAF(G)_NRE	Number of Available Feedstock (GTJ) _ Natural Resource Economics	-	Number of available GTJ wastes available to produce bio-jet fuel under different NRE scenarios.<br><br>NOTE:<br><br>Maximum number of available GTJ wastes is 11.
CDF(G)$_{ac}$	Current Dominant Feedstock (GTJ)	-	GTJ feedstock with the highest bio-jet fuel production for each country.
DPA(G)$_{abc}$	Domestic Plantation Area (GTJ)	ha	Current GTJ domestic feedstock plantation area for each country (with GTJ dominant feedstock).
PA(G)_NRE	Plantation Area (GTJ) _ Natural Resource Economics	ha	Plantation area required to produce bio-jet fuel under different NRE scenarios for each GTJ waste in each country.
WRP(G)_NRE	Water Required for Production (GTJ) _ Natural Resource Economics	L	Total amount of water required for GTJ process to produce bio-jet fuel for each country under different NRE scenarios.
CY(G)_PrP$_{bcde}$	Crop Yield (GTJ) _ Profitable Potential	hg/ha	Quantity produced per hectare of plantation area for each GTJ crops for each country under different COP / JFP and PC condition.
VolCP(G)_PrP$_{bcde}$	Volatility of Crop Production (GTJ) _ Profitable Potential	%	A measure of the tendency for the production quantity of GTJ crop to vary across 15 years for each country under different COP / JFP and PC condition.
VolPDP(G)_PrP$_{bcde}$	Producer Price Volatility (GTJ) _ Profitable Potential	%	A measure of the tendency for the producer price of GTJ crop to vary across 15 years for each country under different COP / JFP and PC condition.
4. Energy Security – Production (NRE)			

CBP_c	Current Bio-Jet Fuel Production	L	Current bio-jet fuel production for each country in real life.
TBP_NRE	Total Bio-Jet Fuel Production _ Natural Resource Economics	L	Total bio-jet fuel production volume under different NRE scenarios.
TBR_NRE	Total Blending Ratio _ Natural Resource Economics	%	Percentage of bio-jet fuel amount that can be blended with conventional jet fuel under different NRE scenarios.
FBR_NRE	Filtered Blending Ratio _ Natural Resource Economics	%	Filtered total blending ratio with the allowable maximum blending ratio regulated by ASTM D7566 – 19 (50%), under different NRE scenarios.
TBEV_NRE	Total Bio-Jet Fuel Export Volume _ Natural Resource Economics	L	Volume of bio-jet fuel that can be exported if the total blending ratio exceeds the maximum blending ratio allowed by ASTM D7566 – 19 (50%), depending on each NRE scenario.
$CGDP\_PrP_{cde}$	Change in Gross Domestic Product _ Profitable Potential	US$	Change in GDP of each country, benefited from bio-jet fuel production under different COP / JFP and PC condition.
TNAF_NRE	Total Number of Available Feedstock _ Natural Resource Economics	-	Total number of available feedstocks (including 3 production pathways) to produce bio-jet fuel under each NRE scenario for each country.<br><br>NOTE:<br><br>Maximum total number of available feedstocks is 39.
5. Energy Security – Energy Use (NRE)			
$CBPE_c$	Current Bio-Jet Fuel Production in Energy	MJ	Current bio-jet fuel production energy content for each country in real life.
TBPE_NRE	Total Bio-Jet Fuel Production in Energy _ Natural Resource Economics	MJ	Total amount of bio-jet fuel energy content that can be produced under different NRE scenarios.
$JFCE_c$	Jet Fuel Consumption in Energy	MJ	Amount of jet fuel used by every country (in terms of energy).
BWE_NRE	Biofuel & Waste Energy _ Natural Resource Economics	MJ	Energy content of biofuels and waste energy sector of each country under different NRE scenarios.
TPES_NRE	Total Primary Energy Supply _ Natural Resource Economics	MJ	Sum of primary energy supply after accounting for the bio-jet fuel produced under each NRE scenario for every country.
EUC_NRE	Energy Use per Capita _ Natural Resource Economics	MJ/capita	Amount of energy used for each person under each NRE scenario for every country.
BPCT_NRE	Biofuel Percentage _ Natural Resource Economics	%	Percentage of biofuels and waste energy sector to the total energy used by each country under different NRE scenarios.
DBPCT_NRE	Difference in Biofuel Percentage _ Natural Resource Economics	%	Percentage difference to the original biofuels and waste energy sector after taking bio-jet fuel production of each country into account, under different NRE scenarios.
BSE_NRE	Bio-Jet Fuel Shares in Energy _ Natural Resource Economics	%	Percentage of bio-jet fuel to the total energy used by each country under each NRE scenario.
RSE_NRE	Renewable Shares in Energy _ Natural Resource Economics	%	Percentage of renewable energy after considering the energy content of bio-jet fuel produced into total primary energy source of a country under different NRE scenarios.
CRSE_NRE	Current Renewable Shares in Energy _ Natural Resource Economics	%	The original percentage of renewable energy in a country without considering the energy content of bio-jet fuel under different NRE scenarios.
DRSE_NRE	Difference in Renewable Shares in Energy _ Natural Resource Economics	%	Percentage change of renewable energy compared to current renewable energy content of each country under different NRE scenarios.
HHI_NRE	Herfindahl-Hirschman Index _ Natural Resource Economics	-	Herfindahl-Hirschman index of each country under different NRE scenarios.<br><br>NOTE:

			High HHI means higher shares of bio-jet fuel energy in each country, thus lowers the competition and increases the saturation in the market, and vice versa.
NJIV_NRE	New Jet Fuel Import Volume _ Natural Resource Economics	L	New jet fuel import volume when bio-jet fuel is produced from the feedstocks of each country under different NRE scenarios.
$CJFID_c$	Current Jet Fuel Import Dependency	%	Percentage of jet fuel import dependency of each country in real life.
ID_NRE	Import Dependency _ Natural Resource Economics	%	Jet fuel import dependency after taking the production of bio-jet fuel from the feedstocks of each country into account, under different NRE scenarios.
6. Energy Security – Environmental Emissions (NRE)			
JFE_{cf}	Jet Fuel Emission	kg	Amount of emission of NO_x, CO, UHC or soot by jet fuel in each country.
BE_NRE	Bio-Jet Fuel Emission _ Natural Resource Economics	kg	Amount of emission of NO_x, CO, UHC or soot by bio-jet fuel in each country under different NRE scenarios.
ER_NRE	Emission Reduction _ Natural Resource Economics	kg	Amount of NO_x, CO, UHC or soot emissions reduced if bio-jet fuel is produced in each country under different NRE scenarios.
PER_NRE	Percentage Emission Reduction _ Natural Resource Economics	%	Percentage reduction of NO_x, CO, UHC or soot emission if bio-jet fuel is produced in each country under different NRE scenarios.
DPA_c	Domestic Plantation Area	ha	Plantation area of the dominant feedstock amongst each production pathway.<br><br>NOTE:<br><br>To prevent any overlapping of the crops' plantation area.
TPA_NRE	Total Plantation Area _ Natural Resource Economics	ha	Total plantation area of feedstock required by each country in each NRE scenario.
LUEm_NRE	Land Use Emission _ Natural Resource Economics	kg CO_2eq	Amount of GHG emission due to the usage (ILUC) of the total plantation area of each country under different NRE scenarios.
FertUEm_NRE	Fertiliser Use Emission _ Natural Resource Economics	kg CO_2eq	Amount of GHG emission due to the usage of nitrogenous fertiliser on the total plantation area of each country under different NRE scenarios.
GHGEm_NRE	GHG Emission _ Natural Resource Economics	kg CO_2eq	Total amount of GHG emission due to bio-jet fuel production under different NRE scenarios.
7. Water Security – Water Stress (NRE)			
$BWSI_c$	Baseline Water Stress Index	-	A measure of the amount of pressure exerted to natural water resources due to agricultural activity in each country. It is the total annual water withdrawals (consider agricultural only, not municipal and industrial) expressed as a percent of the total annual available flow (renewable supply).<br><br>NOTE:<br><br>Higher values indicate more competition among users.
TCWR_NRE	Total Crop Water Required _ Natural Resource Economics	m^3	Total volume of water required to produce the quantity of each crop or feedstock for bio-jet fuel production in each country under different NRE scenarios.
TWR_NRE	Total Water Required _ Natural Resource Economics	L	Total amount of water required to produce bio-jet fuel (from scratch) in each country under different NRE scenarios.
FWSI_NRE	Fraction of Water Stress Index _ Natural Resource Economics	-	Fraction of contribution to the water stress of each country by their corresponding bio-jet fuel production under different NRE scenarios.

WWC_NRE	Water Withdrawal per Capita _ Natural Resource Economics	L/capita	Amount of water being withdrawn from the society in each country due to bio-jet fuel production under each NRE scenario.
TGNWF_NRE	Total Green Water Footprint _ Natural Resource Economics	m^3	Total amount of rainwater used for bio-jet fuel production of each country under each NRE scenario.
TBLWF_NRE	Total Blue Water Footprint _ Natural Resource Economics	m^3	Total amount of irrigation water used for bio-jet fuel production of each country under each NRE scenario.
TGYWF_NRE	Total Grey Water Footprint _ Natural Resource Economics	m^3	Total amount of fresh and groundwater used for bio-jet fuel production of each country under each NRE scenario.
8. Water Security – Agriculture (NRE)			
GNWSI_NRE	Green Water Scarcity Index _ Natural Resource Economics	-	Rainwater insufficiency due to the total green water footprint for bio-jet fuel production in each country under each NRE scenario.
BLWSI_NRE	Blue Water Scarcity Index _ Natural Resource Economics	-	Irrigation water insufficiency due to the total blue water footprint for bio-jet fuel production in each country under each NRE scenario.
GYWSI_NRE	Grey Water Scarcity Index _ Natural Resource Economics	-	Fresh and groundwater insufficiency due to the total grey water footprint for bio-jet fuel production in each country under each NRE scenario.
AD_NRE	Agricultural Dependency _ Natural Resource Economics	%	Dependency of water used in Bio-jet fuel production to agricultural water usage for each NRE scenario.
9. Food Security – Availability (NRE)			
CLC_NRE	Cropland per Capita _ Natural Resource Economics	ha/capita	Total area of cropland used by the society in each country for bio-jet fuel production under different NRE scenarios.
BPC_NRE	Bio-Jet Fuel Production for Crops _ Natural Resource Economics	L	Volume of bio-jet fuel produced from each corresponding crop through OTJ, ETJ and GTJ processes in each country under different NRE scenarios.<br><br>NOTE:<br><br>Be careful especially when calculating across OTJ, ETJ & GTJ processes that share the same corresponding crops, as follows: Barley, Maize, Oats, Oil Palm Fruit, Rice, paddy, and Wheat. Other corresponding crops do not repeat across OTJ, ETJ and GTJ processes.
CY_NRE	Crop Yield _ Natural Resource Economics	hg/ha	Quantity produced from each hectare of plantation area for each crop in each country under different NRE scenarios.<br><br>NOTE:<br><br>Values will be the same for all countries under intensified scenario. Be careful especially when calculating across OTJ, ETJ & GTJ processes that share the same corresponding crops, as follows: Barley, Maize, Oats, Oil Palm Fruit, Rice, paddy, and Wheat. Other corresponding crops do not repeat across OTJ, ETJ and GTJ processes.
TCY_NRE	Total Crop Yield _ Natural Resource Economics	hg/ha	Sum of the quantity produced from each hectare of plantation area for each crop in each country under different NRE scenarios.<br><br>NOTE:<br><br>Values will be the same for all countries under intensified scenario. Be careful especially when calculating across OTJ, ETJ & GTJ processes that share the same corresponding crops, as follows: Barley, Maize, Oats, Oil Palm Fruit, Rice, paddy, and Wheat. Other corresponding crops do not repeat across OTJ, ETJ and GTJ processes.

VolCP_NRE$_{bc}$	Volatility of Crop Production Natural Resource Economics	%	A measure of the tendency for the production quantity of the 28 crops to vary across 15 years for each country under each NRE scenario.
VolAP_NRE	Volatility of Agricultural Production Natural Resource Economics	%	Sum of all the tendency for the production quantity of the 28 crops to vary across 15 years for each country under each NRE scenario.
constVol_P$_{bc}$	Constant Volatility for Crops	%	The volatility portions of each crop produced in every country.
constVol_P$_{c}$	Constant Volatility	%	Sum of all the volatility portions of each crop produced in every country.
varVol_PrP$_{cde}$	Variable Volatility	%	Collective volatility of the crops used for bio-jet fuel production under different COP / JFP and PC condition.
TEF_NRE	Total Energy of Feedstock Natural Resource Economics	MJ	Total energy content of the feedstocks used for bio-jet production in each country under different NRE scenarios.
DES_NRE	Dietary Energy Supply Natural Resource Economics	kcal/capita /day	Amount of dietary energy required by the society (supplied by total energy of feedstock) under different NRE scenarios.
ADESA_NRE	Average Dietary Energy Supply Adequacy (ADESA) Natural Resource Economics	%	The adequacy of food supply in terms of calories after accounting bio-jet fuel production in each country under different NRE scenarios.
FADESA_NRE	Fraction of ADESA Natural Resource Economics	-	Fraction of ADESA in each country under different NRE scenarios
NADESA_NRE	New ADESA Natural Resource Economics	%	New ADESA after taking feedstocks away from the food market of a country (through import quantities) under different NRE scenarios.
CADESA_NRE	Current ADESA Natural Resource Economics	%	ADESA of the current food market if the feedstocks are used to supply food for the society.
NPF_NRE	Number of People Fed Natural Resource Economics	pax	Number of people fed based on the total calorific values of all feedstocks on the average dietary energy requirement under different NRE scenarios.
minADESA$_{c}$	Minimum ADESA	%	Percentage ratio of MDER and ADER of each country.
10. Food Security – Access			
DFD$_{c}$	Depth of Food Deficit	kcal/capita /day	Total calories required to lift undernourished from their status, when everything else being constant, in each country.
PU$_{c}$	Prevalence of Undernourishment	%	The percentage of total population in each country whose food intake is insufficient to meet dietary energy requirements continuously.
11. Food Security – Stability (NRE)			
VolPDP_NRE$_{bc}$	Producer Price Volatility Natural Resource Economics	%	A measure of the tendency for the producer price of each crop to vary across 15 years for each country under different COP / JFP and PC condition.<br><br>NOTE:<br><br>PDP_I$_{bc}$ is the producer price for the country that have intensified crop production, after calculating across 15 years.
VolDCP_NRE	Domestic Crop Price Volatility Natural Resource Economics	%	Sum of all the tendency for the producer price of the 28 crops to vary across 15 years for each country under each NRE scenario.
12. Governance			
VA$_{c}$	Voice and Accountability	%	A measure to reflect perceptions of the extent to which a country's citizens can participate in selecting their government, as well as freedom of expression, freedom of association, and a free media.
PSAV$_{c}$	Political Stability and Absence of Violence	%	A measure to reflect perceptions of the likelihood of political instability and/or politically motivated violence.
GE$_{c}$	Government Effectiveness	%	A measure to capture perceptions of the quality of public services, the quality of the civil service and the degree of its

			independence from political pressures, the quality of policy formulation and implementation, and the credibility of the government's commitment to such policies.
RQ_c	Regulatory Quality	%	A measure to capture perceptions of the ability of the government to formulate and implement sound policies and regulations that. permit and promote private sector development.
RL_c	Rule of Law	%	A measure of law constraint and influence towards the behavior of citizen in each country.
CC_c	Control of Corruption	%	A measure of the public power or bureaucratic regulation exercised for private gain from which it creates corruption in each country, and which may hinder for the foreign investors.

5. Exact Unit Conversion

Mass

From Metric tons to Kilograms

$$1\ t =\ 1 \times 10^3\ kg$$

From Pounds to Kilograms

$$1\ lb =\ 0.45359237\ kg$$

Volume

From U.S. fluid gallons to Litres

$$\begin{aligned}&1\ US\ gal = 231\ in^3 = [231 \times (0.0254)^3]\ m^3 = [231 \times (0.0254)^3 \times 1000]\ L \\ &= 3.785411784\ L\end{aligned}$$

From Barrels of oil to Litres

$$\begin{aligned}&1\ bbl =\ 42\ US\ gal = (42 \times 3.785411784)\ L \\ &= 158.987294928\ L\end{aligned}$$

From Cubic metres to Litres

$$1\ cm^3 = 1 \times 10^3\ L$$

Energy

From Kilotons of oil equivalent to Kilocalories

$$1\ ktoe = 1 \times 10^{10}\ kcal$$

From Kilocalories to Megajoules

$$1\ kcal = 4.1868 \times 10^{-3}\ MJ$$

From Kilotons of oil equivalent to Megajoules

$$\begin{aligned}&1\ ktoe = 4.1868 \times 10^7\ MJ \\ &= 41868000\ MJ\end{aligned}$$

From Megajoules to Kilocalories

$$\begin{aligned}1\ MJ &= \frac{1}{4.1868 \times 10^{-3}}\ kcal \\ &= \frac{1 \times 10^7}{41868}\ kcal\end{aligned}$$

Crude Oil

From Metric tons to Barrels

$$1\ t_{crude\ oil} = 7.333\ bbl_{crude\ oil}$$

- Based on worldwide weighted average

6. Natural Resource Economics (NRE)

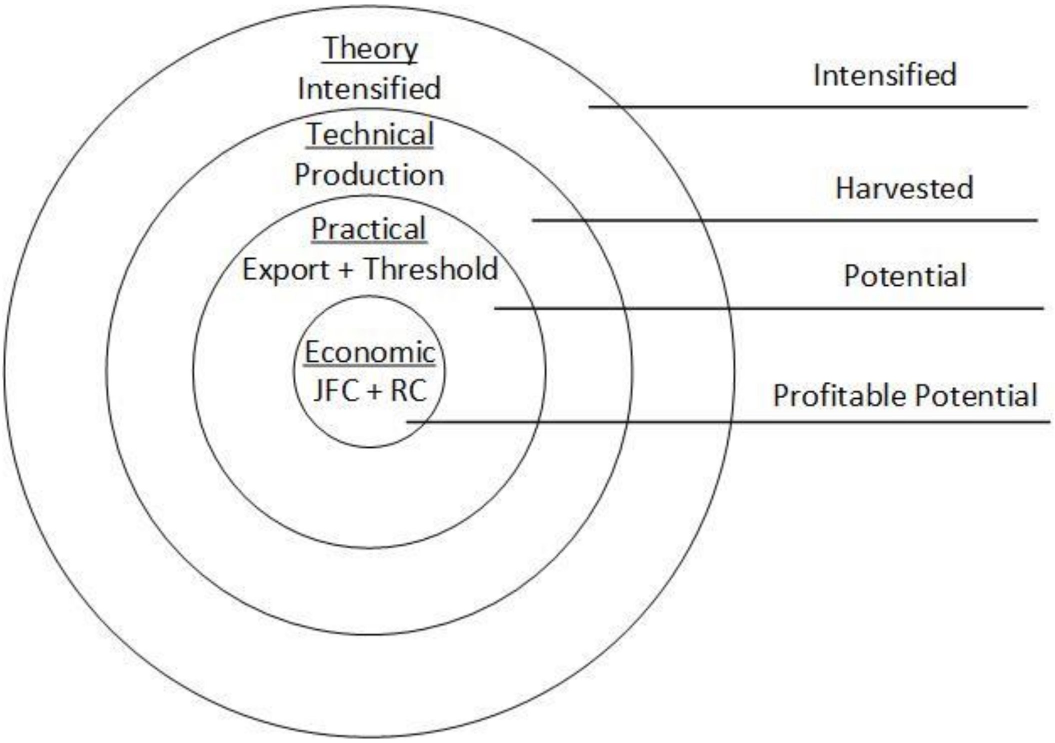

Natural Resource Economics (NRE) will represent

1. Potential sector
2. Profitable Potential sector
3. Harvested sector
4. Intensified sector

7. Equations for the model

A) Oil-to-Jet (OTJ)

1) Feedstock Threshold (OTJ) [$FT(O)_a$]

$$FT(O)_a = 365 \times 10\,000 \times \left(\frac{OC_{ab}}{100}\right)$$

Abbreviation	Name	Units	Description
$FT(O)_a$	Feedstock Threshold (OTJ)	kg	Minimum feedstock quantity to produce bio-jet fuel for every feedstock in each country through OTJ process.<br><br>NOTE:<br><br>Assuming 10 000 kg per day and 365 days per year.
OC_{ab}	Oil Content	wt%	Oil content of different OTJ feedstocks (in weight percent).

2) Production Quantity (OTJ) _ Intensified [$PQ(O)\_I_{bc}$]

$$PQ(O)\_I_{bc} = \frac{HCY(O)_b}{10} \times AH(O)_{bc}$$

Abbreviation	Name	Units	Description
$PQ(O)\_I_{bc}$	Production Quantity (OTJ) _ Intensified	kg	The maximum crop produced for every country to produce bio-jet fuel under OTJ process.
$HCY(O)_b$	Highest Crop Yield (OTJ)	hg/ha	Setting highest yield for every OTJ crop in the world. This is to get the highest crop production for every country by assuming that every country has the best technology for the crop production.<br><br>Multiplied by 0.1 to convert from hecto to kilo.
$AH(O)_{bc}$	Area Harvested (OTJ)	ha	Total land area used for OTJ crop plantation of each country.

3) Crop Consumption (OTJ) [$CC(O)_{abc}$]

Potential:

$$CC(O)_{abc} = (PQ(O)_{bc} - EQ(O)_{ac}) \times 1000$$

Abbreviation	Name	Units	Description
$CC(O)_{abc}$	Crop Consumption (OTJ)	kg	Current crop consumption by every country for the crops being used for OTJ process.
$PQ(O)_{bc}$	Production Quantity (OTJ)	tonnes	Production quantity for each OTJ crop in each country.<br><br>Multiplied by 1000 to convert from tonnes to kilogram.
$EQ(O)_{ac}$	Export Quantity (OTJ)	tonnes	Quantity of each OTJ feedstock exported by each country.<br><br>Multiplied by 1000 to convert from tonnes to kilogram.

4) Lipid Quantity _ Natural Resource Economics [LQ_NRE$_{abc}$]

Intensified:

$$LQ\_I_{abc} = PQ(O)\_I_{bc} \times \left(\frac{OC_{ab}}{100}\right)$$

Harvested:

$$LQ\_H_{abc} = (PQ(O)_{bc} \times 1000) \times \left(\frac{OC_{ab}}{100}\right)$$

Potential:

$$LQ\_P_{abc} = (EQ(O)_{ac} \times 1000) \times \left(\frac{OC_{ab}}{100}\right)$$

Profitable Potential:

$$LQ\_PrP_{abc} = LQ\_P_{abc}$$

Abbreviation	Name	Units	Description
LQ_NRE$_{abc}$	Lipid Quantity _ Natural Resource Economics	kg	Amount of lipid contained in each OTJ feedstock in every country under different NRE scenarios.
PQ(O)_I$_{bc}$	Production Quantity (OTJ) _ Intensified	kg	The maximum crop produced for every country to produce bio-jet fuel under OTJ process.
PQ(O)$_{bc}$	Production Quantity (OTJ)	tonnes	Production quantity for each OTJ crop in each country.<br><br>Multiplied by 1000 to convert from tonnes to kilogram.
EQ(O)$_{ac}$	Export Quantity (OTJ)	tonnes	Quantity of each OTJ feedstock exported by each country.<br><br>Multiplied by 1000 to convert from tonnes to kilogram.
OC$_{ab}$	Oil Content	wt%	Oil content of different OTJ feedstocks (in weight percent).

5) Usable Lipid Quantity _ Potential [ULQ_P$_{abc}$]

$$\begin{gathered} If\ LQ\_P_{abc} > FT(O)_a, \\ then\ ULQ\_P_{abc} = LQ\_P_{abc}, \\ else\ ULQ\_P_{abc} = 0. \end{gathered}$$

Abbreviation	Name	Units	Description
ULQ_P$_{abc}$	Usable Lipid Quantity _ Potential	kg	Amount of lipid of OTJ feedstock that can be used to produce bio-jet fuel (actual usable mass that will be used to produce bio-jet fuel in every country).<br><br>CONDITION:<br><br>Lipid quantity of a particular feedstock must be greater than the feedstock threshold (to ensure large scale utilisation of bio-jet fuel).
LQ_NRE$_{abc}$	Lipid Quantity _ Natural Resource Economics	kg	Amount of lipid contained in each OTJ feedstock in every country under different NRE scenarios.
FT(O)$_a$	Feedstock Threshold (OTJ)	kg	Minimum feedstock quantity to produce bio-jet fuel for every feedstock in each country through OTJ process.

6) Lipid Export Value _ Profitable Potential [LEV_PrP_{abc}]

$$LEV\_PrP_{abc} = \frac{EV(O)_{ac} \times 1000}{LQ\_PrP_{abc}}$$

Abbreviation	Name	Units	Description
LEV_PrP_{abc}	Lipid Export Value _ Profitable Potential	US$/kg	Lipid exported price for every OTJ feedstock in every country.
$EV(O)_{ac}$	Export Value (OTJ)	1000 US$	Total value (US$) of trade export of each OTJ feedstock of each country.<br><br>Multiplied by 1000 to convert from 1000 US$ to US$.
LQ_NRE_{abc}	Lipid Quantity _ Natural Resource Economics	kg	Amount of lipid contained in each OTJ feedstock in every country under different NRE scenarios.

7) Upper Boundary Export Value (OTJ) _ Profitable Potential [$UBEV(O)\_PrP_{abcde}$]

$$UBEV(O)\_PrP_{abcde} = \left[\frac{\left(\frac{JFP_e}{158.987294928}\right)}{JD} - \left(\frac{PC(O)_d}{BD}\right) \right] \times OY$$

Abbreviation	Name	Units	Description
$UBEV(O)\_PrP_{abcde}$	Upper Boundary Export Value (OTJ) _ Profitable Potential	US$/kg	The highest cost that can be spent per kilogram of lipid in feedstock for bio-jet fuel production to be profitable through OTJ process under different COP / JFP and PC condition.
JFP_e	Jet Fuel Price	US$/bbl	Jet fuel price derived from crude oil price based on the market trend.<br><br>NOTE:<br><br>Obtained by multiplying crude oil price (in US$/bbl) with 1.21682489655049. Refer to the economic model (Excel Workbook) for more information. Divided by 158.987294928 to convert from per barrel of oil to per litre.
$PC(O)_d$	Processing Cost (OTJ)	US$/L	Total processing cost per litre of pure bio-jet fuel produced from OTJ feedstock.
JD	Jet Fuel Density	kg/L	Density of jet fuel at a temperature of 15 °C.
BD	Bio-Jet Fuel Density	kg/L	Density of bio-jet fuel at a temperature of 15 °C.
OY	OTJ Yield	-	Yield of bio-jet fuel through OTJ process.

8) Bio-Jet Fuel Production (OTJ) _ Natural Resource Economics [BP(O)_NRE]

Intensified:

$$BP(O)\_I_{abc} = \frac{LQ\_I_{abc} \times OY}{BD}$$

Harvested:

$$BP(O)\_H_{abc} = \frac{LQ\_H_{abc} \times \ OY}{BD}$$

Potential:

$$BP(O)\_P_{abc} = \frac{ULQ\_P_{abc} \times OY}{BD}$$

Profitable Potential:

$$If\ LEV\_PrP_{abc} < UBEV(O)\_PrP_{abcde},$$
$$BP(O)\_PrP_{abcde} = BP(O)\_P_{abc},$$
$$else\ BP(O)\_PrP_{abcde} = 0.$$

Abbreviation	Name	Units	Description
BP(O)_NRE	Bio-Jet Fuel Production (OTJ) _ Natural Resource Economics	L	Bio-jet fuel production volume from OTJ process under different NRE scenarios.
LQ_NRE_{abc}	Lipid Quantity _ Natural Resource Economics	kg	Amount of lipid contained in each OTJ feedstock in every country under different NRE scenarios.
ULQ_P_{abc}	Usable Lipid Quantity _ Potential	kg	Amount of lipid of OTJ feedstock that can be used to produce bio-jet fuel (actual usable mass that will be used to produce bio-jet fuel in every country).<br><br>CONDITION:<br><br>Lipid quantity of a particular feedstock must be greater than the feedstock threshold (to ensure large scale utilisation of bio-jet fuel).
LEV_PrP_{abc}	Lipid Export Value _ Profitable Potential	US$/kg	Lipid exported price for every OTJ feedstock in every country.
$UBEV(O)\_PrP_{abcde}$	Upper Boundary Export Value (OTJ) _ Profitable Potential	US$/kg	The highest cost that can be spent per kilogram of lipid in feedstock for bio-jet fuel production to be profitable through OTJ process under different COP / JFP and PC condition.
OY	OTJ Yield	-	Yield of bio-jet fuel through OTJ process.
BD	Bio-Jet Fuel Density	kg/L	Density of bio-jet fuel at a temperature of 15 °C.

9) Number of Available Feedstock (OTJ) _ Natural Resource Economics [NAF(O)_NRE]

Intensified:

$$If\ BP(O)\_I_{abc} > 0,$$
$$NAF(O)\_I_{ac} = 1,$$
$$else\ NAF(O)\_I_{ac} = 0.$$

Harvested:

$$If\ BP(O)\_H_{abc} > 0,$$
$$NAF(O)\_H_{ac} = 1,$$

$$else\ NAF(O)\_H_{ac} = 0.$$

Potential:

$$If\ BP(O)\_P_{abc} > 0,$$
$$NAF(O)\_P_{ac} = 1,$$
$$else\ NAF(O)\_P_{ac} = 0.$$

Profitable Potential:

$$If\ BP(O)\_PrP_{abcde} > 0,$$
$$NAF(O)\_PrP_{acde} = 1,$$
$$else\ NAF(O)\_PrP_{acde} = 0.$$

Abbreviation	Name	Units	Description
NAF(O)_NRE	Number of Available Feedstock (OTJ) _ Natural Resource Economics	-	Number of available OTJ feedstocks available to produce bio-jet fuel under different NRE scenarios.<br><br>NOTE:<br><br>Maximum number of available OTJ feedstocks is 16.
BP(O)_NRE	Bio-Jet Fuel Production (OTJ) _ Natural Resource Economics	L	Bio-jet fuel production volume from OTJ process under different NRE scenarios.

10) Current Dominant Feedstock (OTJ) [$CDF(O)_{ac}$]

$$CDF(O)_{ac} = Feedstock\ with\ \max[BP(O)\_P_{abc}]$$

Abbreviation	Name	Units	Description
$CDF(O)_{ac}$	Current Dominant Feedstock (OTJ)	-	OTJ feedstock with the highest bio-jet fuel production for each country.
$BP(O)\_P_{abc}$	Bio-Jet Fuel Production (OTJ) _ Potential	L	Bio-jet fuel production volume from OTJ process under potential scenario.

11) Domestic Plantation Area (OTJ) [$DPA(O)_{abc}$]

$$DPA(O)_{abc} = \frac{BP_c \times 10^6}{\left(\frac{EQ(O)_{ac}}{PQ(O)_{bc}} \times \frac{CY(O)_{bc}}{10}\right) \times \left(\frac{OC_{ab}}{100}\right) \times OY} \quad for\ CDF(O)_{ac}\ only$$

Abbreviation	Name	Units	Description
$DPA(O)_{abc}$	Domestic Plantation Area (OTJ)	ha	Current OTJ domestic feedstock plantation area for each country (with OTJ dominant feedstock).
BP_c	Current Bio-Jet Fuel Production	1000 tonnes	Current bio-jet fuel production volume in each country in real life.<br><br>Multiplied by 1×10^6 to convert from 1000 tonnes to kilogram.
$EQ(O)_{ac}$	Export Quantity (OTJ)	tonnes	Quantity of each OTJ feedstock exported by each country.
$PQ(O)_{bc}$	Production Quantity (OTJ)	tonnes	Production quantity for each OTJ crop in each country.
$CY(O)_{bc}$	Crop Yield (OTJ)	hg/ha	Quantity produced from each hectare of plantation area for each OTJ crop in each country.<br><br>Multiplied by 0.1 to convert from hecto to kilo.
$CDF(O)_{ac}$	Current Dominant Feedstock (OTJ)	-	OTJ feedstock with the highest bio-jet fuel production for each country.
OC_{ab}	Oil Content	wt%	Oil content of different OTJ feedstocks (in weight percent).
OY	OTJ Yield	-	Yield of bio-jet fuel through OTJ process.

12) Plantation Area (OTJ) _ Natural Resource Economics [PA(O)_NRE]

Intensified:

$$PA(O)\_I_{abc} = \frac{BP(O)\_I_{abc} \times BD}{\left(\frac{HCY(O)_b}{10}\right) \times \left(\frac{OC_{ab}}{100}\right) \times OY}$$

Harvested:

$$PA(O)\_H_{abc} = \frac{BP(O)\_H_{abc} \times BD}{\left(\frac{CY(O)_{bc}}{10}\right) \times \left(\frac{OC_{ab}}{100}\right) \times OY}$$

Potential:

$$PA(O)\_P_{abc} = \frac{BP(O)\_P_{abc} \times BD}{\left(\frac{EQ(O)_{ac}}{PQ(O)_{bc}} \times \frac{CY(O)_{bc}}{10}\right) \times \left(\frac{OC_{ab}}{100}\right) \times OY}$$

Profitable Potential:

$$If\ BP(O)\_PrP_{abcde} > 0,$$
$$PA(O)\_PrP_{abcde} = PA(O)\_P_{abc},$$
$$else\ PA(O)\_PrP_{abcde} = 0.$$

Abbreviation	Name	Units	Description
PA(O)_NRE	Plantation Area (OTJ) _ Natural Resource Economics	ha	Plantation area required to produce bio-jet fuel under different NRE scenarios for each OTJ feedstock in each country.
BP(O)_NRE	Bio-Jet Fuel Production (OTJ) _ Natural Resource Economics	L	Bio-jet fuel production volume from OTJ process under different NRE scenarios.
$HCY(O)_b$	Highest Crop Yield (OTJ)	hg/ha	Setting highest yield for every OTJ crop in the world. This is to get the highest crop production for every country by assuming that every country has the best technology for the crop production.<br><br>Multiplied by 0.1 to convert from hecto to kilo.
$CY(O)_{bc}$	Crop Yield (OTJ)	hg/ha	Quantity produced from each hectare of plantation area for each OTJ crop in each country.<br><br>Multiplied by 0.1 to convert from hecto to kilo.
$EQ(O)_{ac}$	Export Quantity (OTJ)	tonnes	Quantity of each OTJ feedstock exported by each country.
$PQ(O)_{bc}$	Production Quantity (OTJ)	tonnes	Production quantity for each OTJ crop in each country.
BD	Bio-Jet Fuel Density	kg/L	Density of bio-jet fuel at a temperature of 15 °C.
OC_{ab}	Oil Content	wt%	Oil content of different OTJ feedstocks (in weight percent).
OY	OTJ Yield	-	Yield of bio-jet fuel through OTJ process.

13) Crop Water Required (OTJ) _ Natural Resource Economics [CWR(O)_NRE]

Intensified:

$$CWR(O)\_I_{abc} = PQ(O)\_I_{bc} \times \frac{TWF_{abc}}{1000}$$

Harvested:

$$CWR(O)\_H_{abc} = PQ(O)_{bc} \times TWF_{abc}$$

Potential:

$$\begin{gathered} If\ BP(O)\_P_{abc} > 0, \\ CWR(O)\_P_{abc} = EQ(O)_{ac} \times TWF_{abc}, \\ else\ CWR(O)\_P_{abc} = 0. \end{gathered}$$

Profitable Potential:

$$\begin{gathered} If\ BP(O)\_PrP_{abcde} > 0, \\ CWR(O)\_PrP_{abcde} = CWR(O)\_P_{abc}, \\ else\ CWR(O)\_PrP_{abcde} = 0. \end{gathered}$$

Abbreviation	Name	Units	Description
CWR(O)_NRE	Crop Water Required (OTJ) _ Natural Resource Economics	m^3	Water required to produce each OTJ feedstock for bio-jet fuel production in each country under different NRE scenarios.
$PQ(O)\_I_{bc}$	Production Quantity (OTJ) _ Intensified	kg	The maximum crop produced for every country to produce bio-jet fuel under OTJ process.
$PQ(O)_{bc}$	Production Quantity (OTJ)	tonnes	Production quantity for each OTJ crop in each country.
$EQ(O)_{ac}$	Export Quantity (OTJ)	tonnes	Quantity of each OTJ feedstock exported by each country.
TWF_{abc}	Total Water Footprint	m^3/tonne	Total water required per ton of crop / feedstock production for each country.<br><br>Multiplied by 0.001 to convert from tonnes to kilogram under intensified scenario only.
BP(O)_NRE	Bio-Jet Fuel Production (OTJ) _ Natural Resource Economics	L	Bio-jet fuel production volume from OTJ process under different NRE scenarios.

14) Water Required for Production (OTJ) _ Natural Resource Economics [WRP(O)_NRE]

Intensified:

$$WRP(O)\_I_{abc} = BP(O)\_I_{abc} \times WR(O)$$

Harvested:

$$WRP(O)\_H_{abc} = BP(O)\_H_{abc} \times WR(O)$$

Potential:

$$WRP(O)\_P_{abc} = BP(O)\_P_{abc} \times WR(O)$$

Profitable Potential:

$$WRP(O)\_PrP_{abcde} = BP(O)\_PrP_{abcde} \times WR(O)$$

Abbreviation	Name	Units	Description
WRP(O)_NRE	Water Required for Production (OTJ) _ Natural Resource Economics	L	Total amount of water required for OTJ process to produce bio-jet fuel for each country under different NRE scenarios.
BP(O)_NRE	Bio-Jet Fuel Production (OTJ) _ Natural Resource Economics	L	Bio-jet fuel production volume from OTJ process under different NRE scenarios.
WR(O)	Water Required (OTJ)	$L/L_{bio\text{-}jet}$	Water required to produce 1 litre of bio-jet fuel through OTJ process.

15) Energy of Feedstock (OTJ) _ Natural Resource Economics [EF(O)_NRE]

Intensified:

$$EF(O)\_I_{abc} = FEC_{ab} \times PQ(O)\_I_{bc}$$

Harvested:

$$EF(O)\_H_{abc} = FEC_{ab} \times (PQ(O)_{bc} \times 1000)$$

Potential:

$$\begin{gathered} If\ BP(O)\_P_{abc} > 0, \\ EF(O)\_P_{abc} = FEC_{ab} \times (EQ(O)_{ac} \times 1000), \\ else\ EF(O)\_P_{abc} = 0. \end{gathered}$$

Profitable Potential:

$$\begin{gathered} If\ BP(O)\_PrP_{abcde} > 0, \\ EF(O)\_PrP_{abcde} = EF(O)\_P_{abc}, \\ else\ EF(O)\_PrP_{abcde} = 0. \end{gathered}$$

Abbreviation	Name	Units	Description
EF(O)_NRE	Energy of Feedstock (OTJ) _ Natural Resource Economics	MJ	Energy of each OTJ feedstock used by each country under different NRE scenarios.
$PQ(O)\_I_{bc}$	Production Quantity (OTJ) _ Intensified	kg	The maximum crop produced for every country to produce bio-jet fuel under OTJ process.
$PQ(O)_{bc}$	Production Quantity (OTJ)	tonnes	Production quantity for each OTJ crop in each country.<br><br>Multiplied by 1000 to convert from tonnes to kilogram.
$EQ(O)_{ac}$	Export Quantity (OTJ)	tonnes	Quantity of each OTJ feedstock exported by each country.<br><br>Multiplied by 1000 to convert from tonnes to kilogram.
BP(O)_NRE	Bio-Jet Fuel Production (OTJ) _ Natural Resource Economics	L	Bio-jet fuel production volume from OTJ process under different NRE scenarios.
FEC_{ab}	Feedstock Energy Content	MJ/kg	Energy content in megajoules per kilogram of feedstock.

16) Crop Yield (OTJ) _ Profitable Potential [$CY(O)\_PrP_{bcde}$]

$$CY(O)\_PrP_{bcde} = \frac{BP(O)\_PrP_{abcde}}{BP(O)\_H_{abc} + BP(E)\_H_{abc} + BP(G)\_H_{abc}} \times CY(O)_{bc}$$

Abbreviation	Name	Units	Description
$CY(O)\_PrP_{bcde}$	Crop Yield (OTJ) _ Profitable Potential	hg/ha	Quantity produced per hectare of plantation area for each OTJ crops for each country under different COP / JFP and PC condition.
$BP(O)\_PrP_{abcde}$	Bio-Jet Fuel Production (OTJ) _ Profitable Potential	L	Bio-jet fuel production volume from OTJ process under different COP / JFP and PC condition.
$BP(O)\_H_{abc}$	Bio-Jet Fuel Production (OTJ) _ Harvested	L	Bio-jet fuel production volume from OTJ process under harvested scenario.
$BP(E)\_H_{abc}$	Bio-Jet Fuel Production (ETJ) _ Harvested	L	Bio-jet fuel production volume from ETJ process under harvested scenario.
$BP(G)\_H_{abc}$	Bio-Jet Fuel Production (GTJ) _ Harvested	L	Bio-jet fuel production volume from GTJ process under harvested scenario.
$CY(O)_{bc}$	Crop Yield (OTJ)	hg/ha	Quantity produced from each hectare of plantation area for each OTJ crop in each country.

17) Volatility of Crop Production (OTJ) _ Profitable Potential [$VolCP(O)\_PrP_{bcde}$]

$$VolCP(O)\_PrP_{bcde} = \frac{BP(O)\_PrP_{abcde}}{BP(O)\_P_{abc} + BP(E)\_P_{abc} + BP(G)\_P_{abc}} \times VolCP_{bc}$$

Abbreviation	Name	Units	Description
$VolCP(O)\_PrP_{bcde}$	Volatility of Crop Production (OTJ) _ Profitable Potential	%	A measure of the tendency for the production quantity of OTJ crop to vary across 15 years for each country under different COP / JFP and PC condition.
$BP(O)\_PrP_{abcde}$	Bio-Jet Fuel Production (OTJ) _ Profitable Potential	L	Bio-jet fuel production volume from OTJ process under different COP / JFP and PC condition.
$BP(O)\_P_{abc}$	Bio-Jet Fuel Production (OTJ) _ Potential	L	Bio-jet fuel production volume from OTJ process under potential scenario.
$BP(E)\_P_{abc}$	Bio-Jet Fuel Production (ETJ) _ Potential	L	Bio-jet fuel production volume from ETJ process under potential scenario.
$BP(G)\_P_{abc}$	Bio-Jet Fuel Production (GTJ) _ Potential	L	Bio-jet fuel production volume from GTJ process under potential scenario.
$VolCP_{bc}$	Crop Production Volatility	%	A measure of the tendency for the production quantity of each crop to vary across 15 years in every country.

18) Producer Price Volatility (OTJ) _ Profitable Potential [VolPDP(O)_PrP$_{bcde}$]

$$VolPDP(O)\_PrP_{bcde} = \frac{BP(O)\_PrP_{abcde}}{BP(O)\_P_{abc} + BP(E)\_P_{abc} + BP(G)\_P_{abc}} \times VolPDP_{bc}$$

Abbreviation	Name	Units	Description
VolPDP(O)_PrP$_{bcde}$	Producer Price Volatility (OTJ) _ Profitable Potential	%	A measure of the tendency for the producer price of OTJ crop to vary across 15 years for each country under different COP / JFP and PC condition.
BP(O)_PrP$_{abcde}$	Bio-Jet Fuel Production (OTJ) _ Profitable Potential	L	Bio-jet fuel production volume from OTJ process under different COP / JFP and PC condition.
BP(O)_P$_{abc}$	Bio-Jet Fuel Production (OTJ) _ Potential	L	Bio-jet fuel production volume from OTJ process under potential scenario.
BP(E)_P$_{abc}$	Bio-Jet Fuel Production (ETJ) _ Potential	L	Bio-jet fuel production volume from ETJ process under potential scenario.
BP(G)_P$_{abc}$	Bio-Jet Fuel Production (GTJ) _ Potential	L	Bio-jet fuel production volume from GTJ process under potential scenario.
VolPDP$_{bc}$	Producer Price Volatility	%	A measure of the tendency for the producer price of each crop to vary across 15 years in each country.

B) Ethanol-to-Jet (ETJ)

1) Sugar to Composition Ratio [SCR_{CT}]

$$SCR_{CT} = THY_{CT} \times HE_{CT}$$

Abbreviation	Name	Units	Description
SCR_{CT}	Sugar to Composition Ratio	-	Conversion ratio from sugar/starch to glucose.
THY_{CT}	Theoretical Hydrolysis Yield	-	Theoretical hydrolysis yield of sugar / starch / cellulose / hemicellulose into glucose / xylose.
HE_{CT}	Hydrolysis Efficiency	-	Practical efficiency of hydrolysis process for sugar or starch.

2) Bioethanol Yield [BY_{CT}]

$$BY_{sugar} = SCR_{CT} \times FE \times FY$$

$$BY_{starch} = SCR_{CT} \times FE \times SEE \times FY$$

Abbreviation	Name	Units	Description
BY_{CT}	Bioethanol Yield	-	Conversion ratio from sugar/starch to bioethanol.
SCR_{CT}	Sugar to Composition Ratio	-	Conversion ratio from sugar/starch to glucose.
FE	Fermentation Efficiency	-	Practical efficiency of fermentation process due to formation of by-products such as microorganisms and other chemical products. Sugar is used up for the cellular matter of yeast cell.
SEE	Starch to Ethanol Efficiency	-	Overall efficiency of recovering ethanol from starch.
FY	Fermentation Yield	-	Fermentation theoretical yield.<br><br>NOTE:<br><br>For Glucose: $C_6H_{12}O_6 \rightarrow 2C_2H_5OH + 2CO_2$<br>- 100 grams of glucose produce 51.4g bioethanol and 48.8g carbon dioxide.<br>For Xylose: $3C_5H_{10}O_5 \rightarrow 5C_2H_5OH + 2CO_2$

3) Feedstock Threshold (ETJ) [$FT(E)_a$]

$$FT(E)_a = 365 \times 100\,000 \times \left(\frac{DMP_{ab}}{100}\right)$$

Abbreviation	Name	Units	Description
$FT(E)_a$	Feedstock Threshold (ETJ)	kg	Minimum feedstock quantity to produce bio-jet fuel for every feedstock in each country through ETJ process.<br><br>NOTE:<br><br>Assuming 100 000 kg per day and 365 days per year.
DMP_{ab}	Dry Matter Percentage	%	Percentage of dry matter in each type of ETJ (1st generation) feedstock.

4) Production Quantity (ETJ) _ Intensified [$PQ(E)\_I_{bc}$]

$$PQ(E)\_I_{bc} = \frac{HCY(E)_b}{10} \times AH(E)_{bc}$$

Abbreviation	Name	Units	Description
$PQ(E)\_I_{bc}$	Production Quantity (ETJ) _ Intensified	kg	The maximum crop produced for every country to produce bio-jet fuel under ETJ process.
$HCY(E)_b$	Highest Crop Yield (ETJ)	hg/ha	Setting highest yield for every ETJ crop in the world. This is to get the highest crop production for every country by assuming that every country has the best technology for the crop production.<br><br>Multiplied by 0.1 to convert from hecto to kilo.
$AH(E)_{bc}$	Area Harvested (ETJ)	ha	Total land area used for ETJ crop plantation of each country.

5) Crop Consumption (ETJ) [$CC(E)_{abc}$]

Potential:

$$CC(E)_{abc} = (PQ(E)_{bc} - EQ(E)_{ac}) \times 1000$$

Abbreviation	Name	Units	Description
$CC(E)_{abc}$	Crop Consumption (ETJ)	kg	Current crop consumption by every country for the crops being used for ETJ process.
$PQ(E)_{bc}$	Production Quantity (ETJ)	tonnes	Production quantity for each ETJ crop in each country.<br><br>Multiplied by 1000 to convert from tonnes to kilogram.
$EQ(E)_{ac}$	Export Quantity (ETJ)	tonnes	Quantity of each ETJ feedstock exported by each country.<br><br>Multiplied by 1000 to convert from tonnes to kilogram.

6) Dry Matter Quantity (ETJ) _ Natural Resource Economics [$DMQ(E)\_NRE_{abc}$]

Intensified:

$$DMQ(E)\_I_{abc} = PQ(E)\_I_{bc} \times \left(\frac{DMP_{ab}}{100}\right)$$

Harvested:

$$DMQ(E)\_H_{abc} = (PQ(E)_{bc} \times 1000) \times \left(\frac{DMP_{ab}}{100}\right)$$

Potential:

$$DMQ(E)\_P_{abc} = (EQ(E)_{ac} \times 1000) \times \left(\frac{DMP_{ab}}{100}\right)$$

Abbreviation	Name	Units	Description
$DMQ(E)\_NRE_{abc}$	Dry Matter Quantity (ETJ) _ Natural Resource Economics	kg	Amount of dry matter contained in each ETJ feedstock under different NRE scenarios.
$PQ(E)\_I_{bc}$	Production Quantity (ETJ) _ Intensified	kg	The maximum crop produced for every country to produce bio-jet fuel under ETJ process.
$PQ(E)_{bc}$	Production Quantity (ETJ)	tonnes	Production quantity for each ETJ crop in each country.<br><br>Multiplied by 1000 to convert from tonnes to kilogram.
$EQ(E)_{ac}$	Export Quantity (ETJ)	tonnes	Quantity of each ETJ feedstock exported by each country.<br><br>Multiplied by 1000 to convert from tonnes to kilogram.
DMP_{ab}	Dry Matter Percentage	%	Percentage of dry matter in each type of ETJ (1st generation) feedstock.

7) Usable Dry Matter Quantity (ETJ) _ Potential [$UDMQ(E)\_P_{abc}$]

$$\begin{gathered} If\ DMQ(E)\_P_{abc} > FT(E)_a, \\ then\ UDMQ(E)\_P_{abc} = DMQ(E)\_P_{abc}, \\ else\ UDMQ(E)\_P_{abc} = 0. \end{gathered}$$

Abbreviation	Name	Units	Description
$UDMQ(E)\_P_{abc}$	Usable Dry Matter Quantity (ETJ) _ Potential	kg	Amount of dry matter from ETJ feedstock that can be used to produce bio-jet fuel (actual usable mass that will be used to produce bio-jet fuel in every country).<br><br>CONDITION:<br><br>Dry matter quantity of a particular feedstock must be greater than the feedstock threshold (to ensure large scale utilisation of bio-jet fuel).
$DMQ(E)\_P_{abc}$	Dry Matter Quantity (ETJ) _ Potential	kg	Amount of dry matter contained in each ETJ feedstock under potential scenario.
$FT(E)_a$	Feedstock Threshold (ETJ)	kg	Minimum feedstock quantity to produce bio-jet fuel for every feedstock in each country through ETJ process.

8) Sugar Quantity _ Profitable Potential [SQ_PrP$_{abc}$]

$$SQ\_PrP_{abc} = (EQ(E)_{ac} \times 1000) \times \left(\frac{DMP_{ab}}{100}\right) \times \left(\frac{SSC_{ab}}{100}\right) \times SCR_{CT}$$

Abbreviation	Name	Units	Description
SQ_PrP_{abc}	Sugar Quantity _ Profitable Potential	kg	Quantity of the sugar content from each ETJ feedstock in every country.
$EQ(E)_{ac}$	Export Quantity (ETJ)	tonnes	Quantity of each ETJ feedstock exported by each country.<br><br>Multiplied by 1000 to convert from tonnes to kilogram.
DMP_{ab}	Dry Matter Percentage	%	Percentage of dry matter in each type of ETJ (1st generation) feedstock.
SSC_{ab}	Sugar or Starch Content	wt%	Percentage of sugar or starch present in dry matter of each type of ETJ (1st generation) feedstock.
SCR_{CT}	Sugar to Composition Ratio	-	Conversion ratio from sugar/starch to glucose.

9) Sugar Export Value _ Profitable Potential [SEV_PrP$_{abc}$]

$$SEV\_PrP_{abc} = \frac{EV(E)_{ac} \times 1000}{SQ\_PrP_{abc}}$$

Abbreviation	Name	Units	Description
SEV_PrP_{abc}	Sugar Export Value _ Profitable Potential	US$/kg	Sugar exported price for each ETJ feedstock in every country.
$EV(E)_{ac}$	Export Value (ETJ)	1000 US$	Total value (US$) of trade export of each ETJ feedstock of each country.<br><br>Multiplied by 1000 to convert from 1000 US$ to US$.
SQ_PrP_{abc}	Sugar Quantity _ Profitable Potential	kg	Quantity of the sugar content from each ETJ feedstock in every country.

10) Upper Boundary Export Value (ETJ) _ Profitable Potential [UBEV(E)_PrP$_{abcde}$]

$$UBEV(E)\_PrP_{abcde} = \left[\frac{\left(\frac{JFP_e}{158.987294928} \right)}{JD} - \left(\frac{PC(E)_d}{BD} \right) \right] \times FY \times EY$$

Abbreviation	Name	Units	Description
UBEV(E)_PrP$_{abcde}$	Upper Boundary Export Value (ETJ) _ Profitable Potential	US$/kg	The highest cost that can be spent per kilogram of dry matter in feedstock for bio-jet fuel production to be profitable through ETJ process under different COP / JFP and PC condition.
JFP$_e$	Jet Fuel Price	US$/bbl	Jet fuel price derived from crude oil price based on the market trend.<br><br>NOTE:<br><br>Obtained by multiplying crude oil price (in US$/bbl) with 1.21682489655049. Refer to the economic model (Excel Workbook) for more information. Divided by 158.987294928 to convert from per barrel of oil to per litre.
PC(E)$_d$	Processing Cost (ETJ)	US$/L	Total processing cost per litre of pure bio-jet fuel produced from ETJ feedstock.
JD	Jet Fuel Density	kg/L	Density of jet fuel at a temperature of 15 °C.
BD	Bio-Jet Fuel Density	kg/L	Density of bio-jet fuel at a temperature of 15 °C.
FY	Fermentation Yield	-	Fermentation theoretical yield.<br><br>NOTE:<br><br>For Glucose: $C_6H_{12}O_6 \rightarrow 2C_2H_5OH + 2CO_2$<br>- 100 grams of glucose produce 51.4g bioethanol and 48.8g carbon dioxide.<br>For Xylose: $3C_5H_{10}O_5 \rightarrow 5C_2H_5OH + 2CO_2$
EY	ETJ Yield	-	Yield of bio-jet fuel through ETJ process.

11) Bio-Jet Fuel Production (ETJ) _ Natural Resource Economics [BP(E)_NRE]

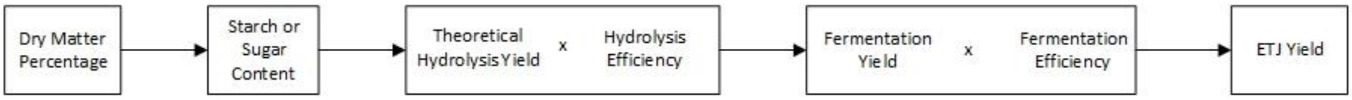

Intensified:

$$BP(E)\_I_{abc} = \frac{DMQ(E)\_I_{abc} \times \left(\frac{SSC_{ab}}{100}\right) \times BY_{CT} \times EY}{BD}$$

Harvested:

$$BP(E)\_H_{abc} = \frac{DMQ(E)\_H_{abc} \times \left(\frac{SSC_{ab}}{100}\right) \times BY_{CT} \times EY}{BD}$$

Potential:

$$BP(E)\_P_{abc} = \frac{UDMQ(E)\_P_{abc} \times \left(\frac{SSC_{ab}}{100}\right) \times BY_{CT} \times EY}{BD}$$

Profitable Potential:

$$If\ SEV\_PrP_{abc} < UBEV(E)\_PrP_{abcde},$$
$$BP(E)\_PrP_{abcde} = BP(E)\_P_{abc},$$
$$else\ BP(E)\_PrP_{abcde} = 0.$$

Abbreviation	Name	Units	Description
BP(E)_NRE	Bio-Jet Fuel Production (ETJ) _ Natural Resource Economics	L	Bio-jet fuel production volume from ETJ process under different NRE scenarios.
$DMQ(E)\_NRE_{abc}$	Dry Matter Quantity (ETJ) _ Natural Resource Economics	kg	Amount of dry matter contained in each ETJ feedstock under different NRE scenarios.
$UDMQ(E)\_P_{abc}$	Usable Dry Matter Quantity (ETJ) Potential	kg	Amount of dry matter from ETJ feedstock that can be used to produce bio-jet fuel (actual usable mass that will be used to produce bio-jet fuel in every country).<br><br>CONDITION:<br><br>Dry matter quantity of a particular feedstock must be greater than the feedstock threshold (to ensure large scale utilisation of bio-jet fuel).
SEV_PrP_{abc}	Sugar Export Value Profitable Potential	US$/kg	Sugar exported price for each ETJ feedstock in every country.
$UBEV(E)\_PrP_{abcde}$	Upper Boundary Export Value (ETJ) Profitable Potential	US$/kg	The highest cost that can be spent per kilogram of dry matter in feedstock for bio-jet fuel production to be profitable through ETJ process under different COP / JFP and PC condition.
SSC_{ab}	Sugar or Starch Content	wt%	Percentage of sugar or starch present in dry matter of each type of ETJ (1st generation) feedstock.
BY_{CT}	Bioethanol Yield	-	Conversion ratio from sugar/starch to bioethanol.
EY	ETJ Yield	-	Yield of bio-jet fuel through ETJ process.
BD	Bio-Jet Fuel Density	kg/L	Density of bio-jet fuel at a temperature of 15 °C.

12) Number of Available Feedstock (ETJ) _ Natural Resource Economics [NAF(E)_NRE]

Intensified:

$$If\ BP(E)\_I_{abc} > 0,$$
$$NAF(E)\_I_{ac} = 1,$$
$$else\ NAF(E)\_I_{ac} = 0.$$

Harvested:

$$If\ BP(E)\_H_{abc} > 0,$$
$$NAF(E)\_H_{ac} = 1,$$
$$else\ NAF(E)\_H_{ac} = 0.$$

Potential:

$$If\ BP(E)\_P_{abc} > 0,$$
$$NAF(E)\_P_{ac} = 1,$$
$$else\ NAF(E)\_P_{ac} = 0.$$

Profitable Potential:

$$If\ BP(E)\_PrP_{abcde} > 0,$$
$$NAF(E)\_PrP_{acde} = 1,$$
$$else\ NAF(E)\_PrP_{acde} = 0.$$

Abbreviation	Name	Units	Description
NAF(E)_NRE	Number of Available Feedstock (ETJ) _ Natural Resource Economics	-	Number of available ETJ feedstocks available to produce bio-jet fuel under different NRE scenarios.<br><br>NOTE:<br><br>Maximum number of available ETJ feedstocks is 12.
BP(E)_NRE	Bio-Jet Fuel Production (ETJ) _ Natural Resource Economics	L	Bio-jet fuel production volume from ETJ process under different NRE scenarios.

13) Current Dominant Feedstock (ETJ) [$CDF(E)_{ac}$]

$$CDF(E)_{ac} = Feedstock\ with\ \max[BP(E)\_P_{abc}]$$

Abbreviation	Name	Units	Description
$CDF(E)_{ac}$	Current Dominant Feedstock (ETJ)	-	ETJ feedstock with the highest bio-jet fuel production for each country.
$BP(E)\_P_{abc}$	Bio-Jet Fuel Production (ETJ) _ Potential	L	Bio-jet fuel production volume from ETJ process under potential scenario.

14) Domestic Plantation Area (ETJ) [$DPA(E)_{abc}$]

$$DPA(E)_{abc} = \frac{BP_c \times 10^6}{\left(\frac{EQ(E)_{ac}}{PQ(E)_{bc}} \times \frac{CY(E)_{bc}}{10}\right) \times \left(\frac{DMP_{ab}}{100}\right) \times \left(\frac{SSC_{ab}}{100}\right) \times BY_{CT} \times EY} \quad for\ CDF(E)_{ac}\ only$$

Abbreviation	Name	Units	Description
$DPA(E)_{abc}$	Domestic Plantation Area (ETJ)	ha	Current ETJ domestic feedstock plantation area for each country (with ETJ dominant feedstock).
BP_c	Current Bio-Jet Fuel Production	1000 tonnes	Current bio-jet fuel production volume in each country in real life.<br><br>Multiplied by 1×10^6 to convert from 1000 tonnes to kilogram.
$EQ(E)_{ac}$	Export Quantity (ETJ)	tonnes	Quantity of each ETJ feedstock exported by each country.
$PQ(E)_{bc}$	Production Quantity (ETJ)	tonnes	Production quantity for each ETJ crop in each country.
$CY(E)_{bc}$	Crop Yield (ETJ)	hg/ha	Quantity produced from each hectare of plantation area for each ETJ crop in each country.<br><br>Multiplied by 0.1 to convert from hecto to kilo.
$CDF(E)_{ac}$	Current Dominant Feedstock (ETJ)	-	ETJ feedstock with the highest bio-jet fuel production for each country.
DMP_{ab}	Dry Matter Percentage	%	Percentage of dry matter in each type of ETJ (1st generation) feedstock.
SSC_{ab}	Sugar or Starch Content	wt%	Percentage of sugar or starch present in dry matter of each type of ETJ (1st generation) feedstock.
BY_{CT}	Bioethanol Yield	-	Conversion ratio from sugar/starch to bioethanol.
EY	ETJ Yield	-	Yield of bio-jet fuel through ETJ process.

15) Plantation Area (ETJ) _ Natural Resource Economics [PA(E)_NRE]

Intensified:

$$PA(E)\_I_{abc} = \frac{BP(E)\_I_{abc} \times BD}{\left(\frac{HCY(E)_b}{10}\right) \times \left(\frac{DMP_{ab}}{100}\right) \times \left(\frac{SSC_{ab}}{100}\right) \times BY_{CT} \times EY}$$

Harvested:

$$PA(E)\_H_{abc} = \frac{BP(E)\_H_{abc} \times BD}{\left(\frac{CY(E)_{bc}}{10}\right) \times \left(\frac{DMP_{ab}}{100}\right) \times \left(\frac{SSC_{ab}}{100}\right) \times BY_{CT} \times EY}$$

Potential:

$$PA(E)\_P_{abc} = \frac{BP(E)\_P_{abc} \times BD}{\left(\frac{EQ(E)_{ac}}{PQ(E)_{bc}} \times \frac{CY(E)_{bc}}{10}\right) \times \left(\frac{DMP_{ab}}{100}\right) \times \left(\frac{SSC_{ab}}{100}\right) \times BY_{CT} \times EY}$$

Profitable Potential:

$$If\ BP(E)\_PrP_{abcde} > 0,$$
$$PA(E)\_PrP_{abcde} = PA(E)\_P_{abc},$$
$$else\ PA(E)\_PrP_{abcde} = 0.$$

Abbreviation	Name	Units	Description
PA(E)_NRE	Plantation Area (ETJ) _ Natural Resource Economics	ha	Plantation area required to produce bio-jet fuel under different NRE scenarios for each ETJ feedstock in each country.
BP(E)_NRE	Bio-Jet Fuel Production (ETJ) _ Natural Resource Economics	L	Bio-jet fuel production volume from ETJ process under different NRE scenarios.
$HCY(E)_b$	Highest Crop Yield (ETJ)	hg/ha	Setting highest yield for every ETJ crop in the world. This is to get the highest crop production for every country by assuming that every country has the best technology for the crop production.<br><br>Multiplied by 0.1 to convert from hecto to kilo.
$CY(E)_{bc}$	Crop Yield (ETJ)	hg/ha	Quantity produced from each hectare of plantation area for each ETJ crop in each country.<br><br>Multiplied by 0.1 to convert from hecto to kilo.
$EQ(E)_{ac}$	Export Quantity (ETJ)	tonnes	Quantity of each ETJ feedstock exported by each country.
$PQ(E)_{bc}$	Production Quantity (ETJ)	tonnes	Production quantity for each ETJ crop in each country.
BD	Bio-Jet Fuel Density	kg/L	Density of bio-jet fuel at a temperature of 15 °C.
DMP_{ab}	Dry Matter Percentage	%	Percentage of dry matter in each type of ETJ (1st generation) feedstock.
SSC_{ab}	Sugar or Starch Content	wt%	Percentage of sugar or starch present in dry matter of each type of ETJ (1st generation) feedstock.
BY_{CT}	Bioethanol Yield	-	Conversion ratio from sugar/starch to bioethanol.
EY	ETJ Yield	-	Yield of bio-jet fuel through ETJ process.

16) Crop Water Required (ETJ) _ Natural Resource Economics [CWR(E)_NRE]

Intensified:

$$CWR(E)\_I_{abc} = PQ(E)\_I_{bc} \times \frac{TWF_{abc}}{1000}$$

Harvested:

$$CWR(E)\_H_{abc} = PQ(E)_{bc} \times TWF_{abc}$$

Potential:

$$If\ BP(E)\_P_{abc} > 0,$$
$$CWR(E)\_P_{abc} = EQ(E)_{ac} \times TWF_{abc},$$
$$else\ CWR(E)\_P_{abc} = 0.$$

Profitable Potential:

$$If\ BP(E)\_PrP_{abcde} > 0,$$
$$CWR(E)\_PrP_{abcde} = CWR(E)\_P_{abc},$$
$$else\ CWR(E)\_PrP_{abcde} = 0.$$

Abbreviation	Name	Units	Description
CWR(E)_NRE	Crop Water Required (ETJ) _ Natural Resource Economics	m^3	Water required to produce each ETJ feedstock for bio-jet fuel production in each country under different NRE scenarios.
$PQ(E)\_I_{bc}$	Production Quantity (ETJ) _ Intensified	kg	The maximum crop produced for every country to produce bio-jet fuel under ETJ process.
$PQ(E)_{bc}$	Production Quantity (ETJ)	tonnes	Production quantity for each ETJ crop in each country.
$EQ(E)_{ac}$	Export Quantity (ETJ)	tonnes	Quantity of each ETJ feedstock exported by each country.
TWF_{abc}	Total Water Footprint	m^3/tonne	Total water required per ton of crop / feedstock production for each country.<br><br>Multiplied by 0.001 to convert from tonnes to kilogram under intensified scenario only.
BP(E)_NRE	Bio-Jet Fuel Production (ETJ) _ Natural Resource Economics	L	Bio-jet fuel production volume from ETJ process under different NRE scenarios.

17) Water Required for Production (ETJ) _ Natural Resource Economics [WRP(E)_NRE]

Intensified:

$$WRP(E)\_I_{abc} = BP(E)\_I_{abc} \times WR(E)$$

Harvested:

$$WRP(E)\_H_{abc} = BP(E)\_H_{abc} \times WR(E)$$

Potential:

$$WRP(E)\_P_{abc} = BP(E)\_P_{abc} \times WR(E)$$

Profitable Potential:

$$WRP(E)\_PrP_{abcde} = BP(E)\_PrP_{abcde} \times WR(E)$$

Abbreviation	Name	Units	Description
WRP(E)_NRE	Water Required for Production (ETJ) _ Natural Resource Economics	L	Total amount of water required for ETJ process to produce bio-jet fuel for each country under different NRE scenarios.
BP(E)_NRE	Bio-Jet Fuel Production (ETJ) _ Natural Resource Economics	L	Bio-jet fuel production volume from ETJ process under different NRE scenarios.
WR(E)	Water Required (ETJ)	$L/L_{bio\text{-}jet}$	Water required to produce 1 litre of bio-jet fuel through ETJ process.

18) Energy of Feedstock (ETJ) _ Natural Resource Economics [EF(E)_NRE]

Intensified:

$$EF(E)\_I_{abc} = FEC_{ab} \times PQ(E)\_I_{bc}$$

Harvested:

$$EF(E)\_H_{abc} = FEC_{ab} \times (PQ(E)_{bc} \times 1000)$$

Potential:

$$If\ BP(E)\_P_{abc} > 0,$$
$$EF(E)\_P_{abc} = FEC_{ab} \times (EQ(E)_{ac} \times 1000),$$
$$else\ EF(E)\_P_{abc} = 0.$$

Profitable Potential:

$$If\ BP(E)\_PrP_{abcde} > 0,$$
$$EF(E)\_PrP_{abcde} = EF(E)\_P_{abc},$$
$$else\ EF(E)\_PrP_{abcde} = 0.$$

Abbreviation	Name	Units	Description
EF(E)_NRE	Energy of Feedstock (ETJ) _ Natural Resource Economics	MJ	Energy of each ETJ feedstock used by each country under different NRE scenarios.
$PQ(E)\_I_{bc}$	Production Quantity (ETJ) _ Intensified	kg	The maximum crop produced for every country to produce bio-jet fuel under ETJ process.
$PQ(E)_{bc}$	Production Quantity (ETJ)	tonnes	Production quantity for each ETJ crop in each country.<br><br>Multiplied by 1000 to convert from tonnes to kilogram.
$EQ(E)_{ac}$	Export Quantity (ETJ)	tonnes	Quantity of each ETJ feedstock exported by each country.<br><br>Multiplied by 1000 to convert from tonnes to kilogram.
BP(E)_NRE	Bio-Jet Fuel Production (ETJ) _ Natural Resource Economics	L	Bio-jet fuel production volume from ETJ process under different NRE scenarios.
FEC_{ab}	Feedstock Energy Content	MJ/kg	Energy content in megajoules per kilogram of feedstock.

19) Crop Yield (ETJ) _ Profitable Potential [CY(E)_PrP$_{bcde}$]

$$CY(E)\_PrP_{bcde} = \frac{BP(E)\_PrP_{abcde}}{BP(O)\_H_{abc} + BP(E)\_H_{abc} + BP(G)\_H_{abc}} \times CY(E)_{bc}$$

Abbreviation	Name	Units	Description
$CY(E)\_PrP_{bcde}$	Crop Yield (ETJ) _ Profitable Potential	hg/ha	Quantity produced per hectare of plantation area for each ETJ crops for each country under different COP / JFP and PC condition.
$BP(E)\_PrP_{abcde}$	Bio-Jet Fuel Production (ETJ) _ Profitable Potential	L	Bio-jet fuel production volume from ETJ process under different COP / JFP and PC condition.
$BP(O)\_H_{abc}$	Bio-Jet Fuel Production (OTJ) _ Harvested	L	Bio-jet fuel production volume from OTJ process under harvested scenario.
$BP(E)\_H_{abc}$	Bio-Jet Fuel Production (ETJ) _ Harvested	L	Bio-jet fuel production volume from ETJ process under harvested scenario.
$BP(G)\_H_{abc}$	Bio-Jet Fuel Production (GTJ) _ Harvested	L	Bio-jet fuel production volume from GTJ process under harvested scenario.
$CY(E)_{bc}$	Crop Yield (ETJ)	hg/ha	Quantity produced from each hectare of plantation area for each ETJ crop in each country.

20) Volatility of Crop Production (ETJ) _ Profitable Potential [VolCP(E)_PrP$_{bcde}$]

$$VolCP(E)\_PrP_{bcde} = \frac{BP(E)\_PrP_{abcde}}{BP(O)\_P_{abc} + BP(E)\_P_{abc} + BP(G)\_P_{abc}} \times VolCP_{bc}$$

Abbreviation	Name	Units	Description
VolCP(E)_PrP$_{bcde}$	Volatility of Crop Production (ETJ) _ Profitable Potential	%	A measure of the tendency for the production quantity of ETJ crop to vary across 15 years for each country under different COP / JFP and PC condition.
BP(E)_PrP$_{abcde}$	Bio-Jet Fuel Production (ETJ) _ Profitable Potential	L	Bio-jet fuel production volume from ETJ process under different COP / JFP and PC condition.
BP(O)_P$_{abc}$	Bio-Jet Fuel Production (OTJ) _ Potential	L	Bio-jet fuel production volume from OTJ process under potential scenario.
BP(E)_P$_{abc}$	Bio-Jet Fuel Production (ETJ) _ Potential	L	Bio-jet fuel production volume from ETJ process under potential scenario.
BP(G)_P$_{abc}$	Bio-Jet Fuel Production (GTJ) _ Potential	L	Bio-jet fuel production volume from GTJ process under potential scenario.
VolCP$_{bc}$	Crop Production Volatility	%	A measure of the tendency for the production quantity of each crop to vary across 15 years in every country.

21) Producer Price Volatility (ETJ) _ Profitable Potential [VolPDP(E)_PrP$_{bcde}$]

$$VolPDP(E)\_PrP_{bcde} = \frac{BP(E)\_PrP_{abcde}}{BP(O)\_P_{abc} + BP(E)\_P_{abc} + BP(G)\_P_{abc}} \times VolPDP_{bc}$$

Abbreviation	Name	Units	Description
VolPDP(E)_PrP$_{bcde}$	Producer Price Volatility (ETJ) _ Profitable Potential	%	A measure of the tendency for the producer price of ETJ crop to vary across 15 years for each country under different COP / JFP and PC condition.
BP(E)_PrP$_{abcde}$	Bio-Jet Fuel Production (ETJ) _ Profitable Potential	L	Bio-jet fuel production volume from ETJ process under different COP / JFP and PC condition.
BP(O)_P$_{abc}$	Bio-Jet Fuel Production (OTJ) _ Potential	L	Bio-jet fuel production volume from OTJ process under potential scenario.
BP(E)_P$_{abc}$	Bio-Jet Fuel Production (ETJ) _ Potential	L	Bio-jet fuel production volume from ETJ process under potential scenario.
BP(G)_P$_{abc}$	Bio-Jet Fuel Production (GTJ) _ Potential	L	Bio-jet fuel production volume from GTJ process under potential scenario.
VolPDP$_{bc}$	Producer Price Volatility	%	A measure of the tendency for the producer price of each crop to vary across 15 years in each country.

C) Gas-to-Jet (GTJ)

1) Feedstock Threshold (GTJ) [$FT(G)_a$]

$$FT(G)_a = 365 \times 100\,000 \times \left(\frac{DMP_{ab}}{100}\right)$$

Abbreviation	Name	Units	Description
$FT(G)_a$	Feedstock Threshold (GTJ)	kg	Minimum feedstock quantity to produce bio-jet fuel for every feedstock in each country through GTJ process.<br><br>NOTE:<br><br>Assuming 100 000 kg per day and 365 days per year.
DMP_{ab}	Dry Matter Percentage	%	Percentage of dry matter in each type of GTJ (2nd generation) feedstock.

2) Production Quantity (GTJ) _ Intensified [$PQ(G)\_I_{bc}$]

$$PQ(G)\_I_{bc} = \frac{HCY(G)_b}{10} \times AH(G)_{bc}$$

Abbreviation	Name	Units	Description
$PQ(G)\_I_{bc}$	Production Quantity (GTJ) _ Intensified	kg	The maximum crop produced for every country to produce bio-jet fuel under GTJ process.
$HCY(G)_b$	Highest Crop Yield (GTJ)	hg/ha	Setting highest yield for every GTJ crop in the world. This is to get the highest crop production for every country by assuming that every country has the best technology for the crop production.<br><br>Multiplied by 0.1 to convert from hecto to kilo.
$AH(G)_{bc}$	Area Harvested (GTJ)	ha	Total land area used for GTJ crop plantation of each country.

3) Dry Matter Quantity (GTJ) _ Natural Resource Economics [$DMQ(G)\_NRE_{abc}$]

Intensified:

$$DMQ(G)\_I_{abc} = PQ(G)\_I_{bc} \times WR_{ab} \times WCR \times \left(\frac{DMP_{ab}}{100}\right)$$

Harvested:

$$DMQ(G)\_H_{abc} = (PQ(G)_{bc} \times 1000) \times WR_{ab} \times WCR \times \left(\frac{DMP_{ab}}{100}\right)$$

Potential:

$$DMQ(G)\_P_{abc} = (PQ(G)_{bc} \times 1000) \times WR_{ab} \times WCR \times \left(\frac{DMP_{ab}}{100}\right)$$

Abbreviation	Name	Units	Description
$DMQ(G)\_NRE_{abc}$	Dry Matter Quantity (GTJ) _ Natural Resource Economics	kg	Amount of 2nd generation dry matter that can be obtained from agricultural waste for GTJ process under different NRE scenarios.
$PQ(G)\_I_{bc}$	Production Quantity (GTJ) _ Intensified	kg	The maximum crop produced for every country to produce bio-jet fuel under GTJ process.
$PQ(G)_{bc}$	Production Quantity (GTJ)	tonnes	Production quantity for each GTJ crop in each country.<br><br>Multiplied by 1000 to convert from tonnes to kilogram.
WR_{ab}	Waste Ratio	%	Percentage of wastes from dry matter of GTJ (2nd generation) feedstock.
WCR	Waste Collectible Ratio	%	Percentage of collectible agricultural waste per unit of total amount of agriculture waste (2nd generation).
DMP_{ab}	Dry Matter Percentage	%	Percentage of dry matter in each type of GTJ (2nd generation) feedstock.

4) Usable Dry Matter Quantity (GTJ) _ Potential [$UDMQ(G)\_P_{abc}$]

$$\begin{gathered} If\ DMQ(G)\_P_{abc} > FT(G)_a, \\ then\ UDMQ(G)\_P_{abc} = DMQ(G)\_P_{abc}, \\ else\ UDMQ(G)\_P_{abc} = 0. \end{gathered}$$

Abbreviation	Name	Units	Description
$UDMQ(G)\_P_{abc}$	Usable Dry Matter Quantity (GTJ) _ Potential	kg	Amount of 2nd generation dry matter from GTJ wastes that can be used to produce bio-jet fuel (actual usable mass that will be used to produce bio-jet fuel in every country).<br><br>CONDITION:<br><br>Dry matter quantity of a particular feedstock must be greater than the feedstock threshold (to ensure large scale utilisation of bio-jet fuel).
$DMQ(G)\_P_{abc}$	Dry Matter Quantity (GTJ) _ Potential	kg	Amount of 2nd generation dry matter that can be obtained from agricultural waste for GTJ process under potential scenario.
$FT(G)_a$	Feedstock Threshold (GTJ)	kg	Minimum feedstock quantity to produce bio-jet fuel for every feedstock in each country through GTJ process.

5) Dry Matter Value _ Profitable Potential [DMV_PrP$_a$]

$$DMV\_PrP_a = \left(\frac{FC_a}{1000}\right)$$

Abbreviation	Name	Units	Description
DMV_PrP$_a$	Dry Matter Value _ Profitable Potential	US$/kg	2nd generation dry matter quantity price for each country.
FC$_a$	Feedstock Cost	US$/tonne	Cost of feedstock per tonne for every 2nd generation feedstock, from producer perspective.<br><br>Multiplied by 0.001 to convert from per dry tonne to per kilogram.

6) Upper Boundary Export Value (GTJ) _ Profitable Potential [UBEV(G)_PrP$_{abcde}$]

$$UBEV(G)\_PrP_{abcde} = \left[\frac{\left(\frac{JFP_e}{158.987294928}\right)}{JD} - \left(\frac{PC(G)_d}{BD}\right)\right] \times GY - \frac{PTC}{1000}$$

Abbreviation	Name	Units	Description
UBEV(G)_PrP$_{abcde}$	Upper Boundary Export Value (GTJ) _ Profitable Potential	US$/kg	The highest cost that can be spent per kilogram of dry matter in feedstock for bio-jet fuel production to be profitable through GTJ process under different COP / JFP and PC condition.
JFP$_e$	Jet Fuel Price	US$/bbl	Jet fuel price derived from crude oil price based on the market trend.<br><br>NOTE:<br><br>Obtained by multiplying crude oil price (in US$/bbl) with 1.21682489655049. Refer to the economic model (Excel Workbook) for more information. Divided by 158.987294928 to convert from per barrel of oil to per litre.
PC(G)$_d$	Processing Cost (GTJ)	US$/L	Total processing cost per litre of pure bio-jet fuel produced from GTJ feedstock.
JD	Jet Fuel Density	kg/L	Density of jet fuel at a temperature of 15 °C.
BD	Bio-Jet Fuel Density	kg/L	Density of bio-jet fuel at a temperature of 15 °C.
GY	GTJ Yield	-	Yield of bio-jet fuel through GTJ process.
PTC	Pretreatment Cost	US$/tonne	Drying cost per fry tonne of 2nd generation feedstock for GTJ (gasification) process.<br><br>Multiplied by 0.001 to convert from per dry tonne to per kilogram.

7) Bio-Jet Fuel Production (GTJ) _ Natural Resource Economics [BP(G)_NRE]

Intensified:

$$BP(G)\_I_{abc} = \frac{DMQ(G)\_I_{abc} \times GY}{BD}$$

Harvested:

$$BP(G)\_H_{abc} = \frac{DMQ(G)\_H_{abc} \times GY}{BD}$$

Potential:

$$BP(G)\_P_{abc} = \frac{UDMQ(G)\_P_{abc} \times GY}{BD}$$

Profitable Potential:

$$If\ DMV\_PrP_a < UBEV(G)\_PrP_{abcde},$$
$$BP(G)\_PrP_{abcde} = BP(G)\_P_{abc},$$
$$else\ BP(G)\_PrP_{abcde} = 0.$$

Abbreviation	Name	Units	Description
BP(G)_NRE	Bio-Jet Fuel Production (GTJ) _ Natural Resource Economics	L	Bio-jet fuel production volume from GTJ process under different NRE scenarios.
$DMQ(G)\_NRE_{abc}$	Dry Matter Quantity (GTJ) _ Natural Resource Economics	kg	Amount of 2nd generation dry matter that can be obtained from agricultural waste for GTJ process under different NRE scenarios.
$UDMQ(G)\_P_{abc}$	Usable Dry Matter Quantity (GTJ) _ Potential	kg	Amount of 2nd generation dry matter from GTJ wastes that can be used to produce bio-jet fuel (actual usable mass that will be used to produce bio-jet fuel in every country).<br><br>CONDITION:<br><br>Dry matter quantity of a particular feedstock must be greater than the feedstock threshold (to ensure large scale utilisation of bio-jet fuel).
DMV_PrP_a	Dry Matter Value _ Profitable Potential	US$/kg	2nd generation dry matter quantity price for each country.
$UBEV(G)\_PrP_{abcde}$	Upper Boundary Export Value (GTJ) _ Profitable Potential	US$/kg	The highest cost that can be spent per kilogram of dry matter in feedstock for bio-jet fuel production to be profitable through GTJ process under different COP / JFP and PC condition.
GY	GTJ Yield	-	Yield of bio-jet fuel through GTJ process.
BD	Bio-Jet Fuel Density	kg/L	Density of bio-jet fuel at a temperature of 15 °C.

8) Number of Available Feedstock (GTJ) _ Natural Resource Economics [NAF(G)_NRE]

Intensified:

$$If\ BP(G)\_I_{abc} > 0,$$
$$NAF(G)\_I_{ac} = 1,$$
$$else\ NAF(G)\_I_{ac} = 0.$$

Harvested:

$$If\ BP(G)\_H_{abc} > 0,$$
$$NAF(G)\_H_{ac} = 1,$$
$$else\ NAF(G)\_H_{ac} = 0.$$

Potential:

$$If\ BP(G)\_P_{abc} > 0,$$
$$NAF(G)\_P_{ac} = 1,$$
$$else\ NAF(G)\_P_{ac} = 0.$$

Profitable Potential:

$$If\ BP(G)\_PrP_{abcde} > 0,$$
$$NAF(G)\_PrP_{acde} = 1,$$
$$else\ NAF(G)\_PrP_{acde} = 0.$$

Abbreviation	Name	Units	Description
NAF(G)_NRE	Number of Available Feedstock (GTJ) _ Natural Resource Economics	-	Number of available GTJ wastes available to produce bio-jet fuel under different NRE scenarios.<br><br>NOTE:<br><br>Maximum number of available GTJ wastes is 11.
BP(G)_NRE	Bio-Jet Fuel Production (GTJ) _ Natural Resource Economics	L	Bio-jet fuel production volume from GTJ process under different NRE scenarios.

9) Current Dominant Feedstock (GTJ) [$CDF(G)_{ac}$]

$$CDF(G)_{ac} = Feedstock\ with\ \max[BP(G)\_P_{abc}]$$

Abbreviation	Name	Units	Description
$CDF(G)_{ac}$	Current Dominant Feedstock (GTJ)	-	GTJ feedstock with the highest bio-jet fuel production for each country.
$BP(G)\_P_{abc}$	Bio-Jet Fuel Production (GTJ) _ Potential	L	Bio-jet fuel production volume from GTJ process under potential scenario.

10) Domestic Plantation Area (GTJ) [$DPA(G)_{abc}$]

$$DPA(G)_{abc} = \frac{BP_c \times 10^6}{\left(\frac{CY(G)_{bc}}{10}\right) \times WR_{ab} \times WCR \times \left(\frac{DMP_{ab}}{100}\right) \times GY} \; for\ CDF(G)_{ac}\ only$$

Abbreviation	Name	Units	Description
$DPA(G)_{abc}$	Domestic Plantation Area (GTJ)	ha	Current GTJ domestic feedstock plantation area for each country (with GTJ dominant feedstock).
BP_c	Current Bio-Jet Fuel Production	1000 tonnes	Current bio-jet fuel production volume in each country in real life.<br><br>Multiplied by 1×10^6 to convert from 1000 tonnes to kilogram.
$CY(G)_{bc}$	Crop Yield (GTJ)	hg/ha	Quantity produced from each hectare of plantation area for each GTJ crop in each country.<br><br>Multiplied by 0.1 to convert from hecto to kilo.
$CDF(G)_{ac}$	Current Dominant Feedstock (GTJ)	-	GTJ feedstock with the highest bio-jet fuel production for each country.
WR_{ab}	Waste Ratio	%	Percentage of wastes from dry matter of GTJ (2nd generation) feedstock.
WCR	Waste Collectible Ratio	%	Percentage of collectible agricultural waste per unit of total amount of agriculture waste (2nd generation).
DMP_{ab}	Dry Matter Percentage	%	Percentage of dry matter in each type of GTJ (2nd generation) feedstock.
GY	GTJ Yield	-	Yield of bio-jet fuel through GTJ process.

11) Plantation Area (GTJ) _ Natural Resource Economics [PA(G)_NRE]

Intensified:

$$PA(G)\_I_{abc} = \frac{BP(G)\_I_{abc} \times BD}{\left(\frac{HCY(G)_b}{10}\right) \times WR_{ab} \times WCR \times \left(\frac{DMP_{ab}}{100}\right) \times GY}$$

Harvested:

$$PA(G)\_H_{abc} = \frac{BP(G)\_H_{abc} \times BD}{\left(\frac{CY(G)_{bc}}{10}\right) \times WR_{ab} \times WCR \times \left(\frac{DMP_{ab}}{100}\right) \times GY}$$

Potential:

$$PA(G)\_P_{abc} = \frac{BP(G)\_P_{abc} \times BD}{\left(\frac{CY(G)_{bc}}{10}\right) \times WR_{ab} \times WCR \times \left(\frac{DMP_{ab}}{100}\right) \times GY}$$

Profitable Potential:

$$If\ BP(G)\_PrP_{abcde} > 0,$$
$$PA(G)\_PrP_{abcde} = PA(G)\_P_{abc},$$
$$else\ PA(G)\_PrP_{abcde} = 0.$$

Abbreviation	Name	Units	Description
PA(G)_NRE	Plantation Area (GTJ) _ Natural Resource Economics	ha	Plantation area required to produce bio-jet fuel under different NRE scenarios for each GTJ waste in each country.
BP(G)_NRE	Bio-Jet Fuel Production (GTJ) _ Natural Resource Economics	L	Bio-jet fuel production volume from GTJ process under different NRE scenarios.
$HCY(G)_b$	Highest Crop Yield (GTJ)	hg/ha	Setting highest yield for every GTJ crop in the world. This is to get the highest crop production for every country by assuming that every country has the best technology for the crop production.<br><br>Multiplied by 0.1 to convert from hecto to kilo.
$CY(G)_{bc}$	Crop Yield (GTJ)	hg/ha	Quantity produced from each hectare of plantation area for each GTJ crop in each country.<br><br>Multiplied by 0.1 to convert from hecto to kilo.
BD	Bio-Jet Fuel Density	kg/L	Density of bio-jet fuel at a temperature of 15 °C.
WR_{ab}	Waste Ratio	%	Percentage of wastes from dry matter of GTJ (2nd generation) feedstock.
WCR	Waste Collectible Ratio	%	Percentage of collectible agricultural waste per unit of total amount of agriculture waste (2nd generation).
DMP_{ab}	Dry Matter Percentage	%	Percentage of dry matter in each type of GTJ (2nd generation) feedstock.
GY	GTJ Yield	-	Yield of bio-jet fuel through GTJ process.

12) Water Required for Production (GTJ) _ Natural Resource Economics [WRP(G)_NRE]

Intensified:

$$WRP(G)\_I_{abc} = BP(G)\_I_{abc} \times WR(G)$$

Harvested:

$$WRP(G)\_H_{abc} = BP(G)\_H_{abc} \times WR(G)$$

Potential:

$$WRP(G)\_P_{abc} = BP(G)\_P_{abc} \times WR(G)$$

Profitable Potential:

$$WRP(G)\_PrP_{abcde} = BP(G)\_PrP_{abcde} \times WR(G)$$

Abbreviation	Name	Units	Description
WRP(G)_NRE	Water Required for Production (GTJ) _ Natural Resource Economics	L	Total amount of water required for GTJ process to produce bio-jet fuel for each country under different NRE scenarios.
BP(G)_NRE	Bio-Jet Fuel Production (GTJ) _ Natural Resource Economics	L	Bio-jet fuel production volume from GTJ process under different NRE scenarios.
WR(G)	Water Required (GTJ)	$L/L_{bio\text{-}jet}$	Water required to produce 1 litre of bio-jet fuel through GTJ process.

13) Crop Yield (GTJ) _ Profitable Potential [$CY(G)\_PrP_{bcde}$]

$$CY(G)\_PrP_{bcde} = \frac{BP(G)\_PrP_{abcde}}{BP(O)\_H_{abc} + BP(E)\_H_{abc} + BP(G)\_H_{abc}} \times CY(G)_{bc}$$

Abbreviation	Name	Units	Description
$CY(G)\_PrP_{bcde}$	Crop Yield (GTJ) _ Profitable Potential	hg/ha	Quantity produced per hectare of plantation area for each GTJ crops for each country under different COP / JFP and PC condition.
$BP(G)\_PrP_{abcde}$	Bio-Jet Fuel Production (GTJ) _ Profitable Potential	L	Bio-jet fuel production volume from GTJ process under different COP / JFP and PC condition.
$BP(O)\_H_{abc}$	Bio-Jet Fuel Production (OTJ) _ Harvested	L	Bio-jet fuel production volume from OTJ process under harvested scenario.
$BP(E)\_H_{abc}$	Bio-Jet Fuel Production (ETJ) _ Harvested	L	Bio-jet fuel production volume from ETJ process under harvested scenario.
$BP(G)\_H_{abc}$	Bio-Jet Fuel Production (GTJ) _ Harvested	L	Bio-jet fuel production volume from GTJ process under harvested scenario.
$CY(G)_{bc}$	Crop Yield (GTJ)	hg/ha	Quantity produced from each hectare of plantation area for each GTJ crop in each country.

14) Volatility of Crop Production (GTJ) _ Profitable Potential [$VolCP\_PrP_{bcde}$]

$$VolCP(G)\_PrP_{bcde} = \frac{BP(G)\_PrP_{abcde}}{BP(O)\_P_{abc} + BP(E)\_P_{abc} + BP(G)\_P_{abc}} \times VolCP_{bc}$$

Abbreviation	Name	Units	Description
$VolCP(G)\_PrP_{bcde}$	Volatility of Crop Production (GTJ) _ Profitable Potential	%	A measure of the tendency for the production quantity of GTJ crop to vary across 15 years for each country under different COP / JFP and PC condition.
$BP(G)\_PrP_{abcde}$	Bio-Jet Fuel Production (GTJ) _ Profitable Potential	L	Bio-jet fuel production volume from GTJ process under different COP / JFP and PC condition.
$BP(O)\_P_{abc}$	Bio-Jet Fuel Production (OTJ) _ Potential	L	Bio-jet fuel production volume from OTJ process under potential scenario.
$BP(E)\_P_{abc}$	Bio-Jet Fuel Production (ETJ) _ Potential	L	Bio-jet fuel production volume from ETJ process under potential scenario.
$BP(G)\_P_{abc}$	Bio-Jet Fuel Production (GTJ) _ Potential	L	Bio-jet fuel production volume from GTJ process under potential scenario.
$VolCP_{bc}$	Crop Production Volatility	%	A measure of the tendency for the production quantity of each crop to vary across 15 years in every country.

15) Producer Price Volatility (GTJ) _ Profitable Potential [$VolPDP(G)\_PrP_{bcde}$]

$$VolPDP(G)\_PrP_{bcde} = \frac{BP(G)\_PrP_{abcde}}{BP(O)\_P_{abc} + BP(E)\_P_{abc} + BP(G)\_P_{abc}} \times VolPDP_{bc}$$

Abbreviation	Name	Units	Description
$VolPDP(G)\_PrP_{bcde}$	Producer Price Volatility (GTJ) _ Profitable Potential	%	A measure of the tendency for the producer price of GTJ crop to vary across 15 years for each country under different COP / JFP and PC condition.
$BP(G)\_PrP_{abcde}$	Bio-Jet Fuel Production (GTJ) _ Profitable Potential	L	Bio-jet fuel production volume from GTJ process under different COP / JFP and PC condition.
$BP(O)\_P_{abc}$	Bio-Jet Fuel Production (OTJ) _ Potential	L	Bio-jet fuel production volume from OTJ process under potential scenario.
$BP(E)\_P_{abc}$	Bio-Jet Fuel Production (ETJ) _ Potential	L	Bio-jet fuel production volume from ETJ process under potential scenario.
$BP(G)\_P_{abc}$	Bio-Jet Fuel Production (GTJ) _ Potential	L	Bio-jet fuel production volume from GTJ process under potential scenario.
$VolPDP_{bc}$	Producer Price Volatility	%	A measure of the tendency for the producer price of each crop to vary across 15 years in each country.

D) Energy Security – Production (NRE)

1) Current Bio-Jet Fuel Production [CBP_c]

$$CBP_c = \frac{BP_c \times 10^6}{BD}$$

Abbreviation	Name	Units	Description
CBP_c	Current Bio-Jet Fuel Production	L	Current bio-jet fuel production for each country in real life.
BP_c	Current Bio-Jet Fuel Production	1000 tonnes	Current bio-jet fuel production volume in each country in real life.<br><br>Multiplied by 1×10^6 to convert from 1000 tonnes to kilogram.
BD	Bio-Jet Fuel Density	kg/L	Density of bio-jet fuel at a temperature of 15 °C.

2) Total Bio-Jet Fuel Production _ Natural Resource Economics [TBP_NRE]

Intensified:

$$TBP\_I_c = \sum BP(O)\_I_{abc} + \sum BP(E)\_I_{abc} + \sum BP(G)\_I_{abc}$$

Harvested:

$$TBP\_H_c = \sum BP(O)\_H_{abc} + \sum BP(E)\_H_{abc} + \sum BP(G)\_H_{abc}$$

Potential:

$$TBP\_P_c = \sum BP(O)\_P_{abc} + \sum BP(E)\_P_{abc} + \sum BP(G)\_P_{abc}$$

Profitable Potential:

$$TBP\_PrP_{cde} = \sum BP(O)\_PrP_{abcde} + \sum BP(E)\_PrP_{abcde} + \sum BP(G)\_PrP_{abcde}$$

Abbreviation	Name	Units	Description
TBP_NRE	Total Bio-Jet Fuel Production _ Natural Resource Economics	L	Total bio-jet fuel production volume under different NRE scenarios.
BP(O)_NRE	Bio-Jet Fuel Production (OTJ) _ Natural Resource Economics	L	Bio-jet fuel production volume from OTJ process under different NRE scenarios.
BP(E)_NRE	Bio-Jet Fuel Production (ETJ) _ Natural Resource Economics	L	Bio-jet fuel production volume from ETJ process under different NRE scenarios.
BP(G)_NRE	Bio-Jet Fuel Production (GTJ) _ Natural Resource Economics	L	Bio-jet fuel production volume from GTJ process under different NRE scenarios.

3) Total Blending Ratio _ Natural Resource Economics [TBR_NRE]

Intensified:

$$TBR\_I_c = \frac{TBP\_I_c \times JD}{(JFC_c \times 10^6)} \times 100\%$$

Harvested:

$$TBR\_H_c = \frac{TBP\_H_c \times JD}{(JFC_c \times 10^6)} \times 100\%$$

Potential:

$$TBR\_P_c = \frac{(TBP\_P_c + CBP_c) \times JD}{(JFC_c \times 10^6)} \times 100\%$$

Profitable Potential:

$$TBR\_PrP_{cde} = \frac{(TBP\_PrP_{cde} + CBP_c) \times JD}{(JFC_c \times 10^6)} \times 100\%$$

Abbreviation	Name	Units	Description
TBR_NRE	Total Blending Ratio _ Natural Resource Economics	%	Percentage of bio-jet fuel amount that can be blended with conventional jet fuel under different NRE scenarios.
TBP_NRE	Total Bio-Jet Fuel Production _ Natural Resource Economics	L	Total bio-jet fuel production volume under different NRE scenarios.
CBP_c	Current Bio-Jet Fuel Production	L	Current bio-jet fuel production for each country in real life.
JFC_c	Jet Fuel Consumption	1000 tonnes	Amount of jet fuel consumed by each country.<br><br>Multiplied by 1×10^6 to convert from 1000 tonnes to kilogram.
JD	Jet Fuel Density	kg/L	Density of jet fuel at a temperature of 15 °C.

4) Filtered Blending Ratio _ Natural Resource Economics [FBR_NRE]

Intensified:

$$If\ TBR\_I_c > MBR,$$
$$FBR\_I_c = MBR,$$
$$else\ FBR\_I_c = TBR\_I_c.$$

Harvested:

$$If\ TBR\_H_c > MBR,$$
$$FBR\_H_c = MBR,$$
$$else\ FBR\_H_c = TBR\_H_c.$$

Potential:

$$If\ TBR\_P_c > MBR,$$
$$FBR\_P_c = MBR,$$
$$else\ FBR\_P_c = TBR\_P_c.$$

Profitable Potential:

$$If\ TBR\_PrP_{cde} > MBR,$$
$$FBR\_PrP_{cde} = MBR,$$
$$else\ FBR\_PrP_{cde} = TBR\_PrP_{cde}.$$

Abbreviation	Name	Units	Description
FBR_NRE	Filtered Blending Ratio _ Natural Resource Economics	%	Filtered total blending ratio with the allowable maximum blending ratio regulated by ASTM D7566 – 19 (50%), under different NRE scenarios.
TBR_NRE	Total Blending Ratio _ Natural Resource Economics	%	Percentage of bio-jet fuel amount that can be blended with conventional jet fuel under different NRE scenarios.
MBR	Maximum Blending Ratio	%	Maximum allowable blending ratio by the regulation (ASTMD7566 – 19).<br><br>NOTE:<br><br>Current regulated maximum blending ratio for Oil-to-Jet (HEFA), Ethanol-to-Jet (ATJ), Gas-to-Jet (FT process) is 50%.

5) Natural Resource Economics [TBEV_NRE]

Intensified:

$$If\ FBR\_I_c = MBR,$$
$$TBEV\_I_c = TBP\_I_c - \frac{(JFC_c \times 10^6)}{JD} \times \left(\frac{FBR\_I_c}{100}\right),$$
$$else\ TBEV\_I_c = 0.$$

Harvested:

$$If\ FBR\_H_c = MBR,$$
$$TBEV\_H_c = TBP\_H_c - \frac{(JFC_c \times 10^6)}{JD} \times \left(\frac{FBR\_H_c}{100}\right),$$

$$else\ TBEV\_H_c = 0.$$

Potential:

$$If\ FBR\_P_c = MBR,$$
$$TBEV\_P_c = (TBP\_P_c + CBP_c) - \frac{(JFC_c \times 10^6)}{JD} \times \left(\frac{FBR\_P_c}{100}\right),$$
$$else\ TBEV\_P_c = 0.$$

Profitable Potential:

$$If\ FBR\_PrP_{cde} = MBR,$$
$$TBEV\_PrP_{cde} = (TBP\_PrP_{cde} + CBP_c) - \frac{(JFC_c \times 10^6)}{JD} \times \left(\frac{FBR\_PrP_{cde}}{100}\right),$$
$$else\ TBEV\_PrP_{cde} = 0.$$

Abbreviation	Name	Units	Description
TBEV_NRE	Total Bio-Jet Fuel Export Volume _ Natural Resource Economics	L	Volume of bio-jet fuel that can be exported if the total blending ratio exceeds the maximum blending ratio allowed by ASTM D7566 – 19 (50%), depending on each NRE scenario.
FBR_NRE	Filtered Blending Ratio _ Natural Resource Economics	%	Filtered total blending ratio with the allowable maximum blending ratio regulated by ASTM D7566 – 19 (50%), under different NRE scenarios.
MBR	Maximum Blending Ratio	%	Maximum allowable blending ratio by the regulation (ASTMD7566 – 19).<br><br>NOTE:<br><br>Current regulated maximum blending ratio for Oil-to-Jet (HEFA), Ethanol-to-Jet (ATJ), Gas-to-Jet (FT process) is 50%.
TBP_NRE	Total Bio-Jet Fuel Production _ Natural Resource Economics	L	Total bio-jet fuel production volume under different NRE scenarios.
CBP_c	Current Bio-Jet Fuel Production	L	Current bio-jet fuel production for each country in real life.
JFC_c	Jet Fuel Consumption	1000 tonnes	Amount of jet fuel consumed by each country.<br><br>Multiplied by 1×10^6 to convert from 1000 tonnes to kilogram.
JD	Jet Fuel Density	kg/L	Density of jet fuel at a temperature of 15 °C.

6) Change in Gross Domestic Product _ Profitable Potential [$CGDP\_PrP_{cde}$]

$$CGDP\_PrP_{cde} = \left(MFSP(O) \times \sum BP(O)\_PrP_{abcde}\right) + \left(MFSP(E) \times \sum BP(E)\_PrP_{abcde}\right) + \left(MFSP(G) \times \sum BP(G)\_PrP_{abcde}\right) - \left(\sum EV(O)_{ac} + \sum EV(E)_{ac}\right) \times 1000 - \left\{\left(\frac{JFP_e}{158.987294928}\right) \times \left[\frac{(JFC_c \times 10^6)}{JD} \times \left(\frac{FBR\_PrP_{cde}}{100}\right)\right]\right\}$$

Abbreviation	Name	Units	Description
$CGDP\_PrP_{cde}$	Change in Gross Domestic Product _ Profitable Potential	US$	Change in GDP of each country, benefited from bio-jet fuel production under different COP / JFP and PC condition.
$BP(O)\_PrP_{abcde}$	Bio-Jet Fuel Production (OTJ) _ Profitable Potential	L	Bio-jet fuel production volume from OTJ process under different COP / JFP and PC condition.
$BP(E)\_PrP_{abcde}$	Bio-Jet Fuel Production (ETJ) _ Profitable Potential	L	Bio-jet fuel production volume from ETJ process under different COP / JFP and PC condition.
$BP(G)\_PrP_{abcde}$	Bio-Jet Fuel Production (GTJ) _ Profitable Potential	L	Bio-jet fuel production volume from GTJ process under different COP / JFP and PC condition.
$EV(O)_{ac}$	Export Value (OTJ)	1000 US$	Total value (US$) of trade export of each OTJ feedstock of each country.<br><br>Multiplied by 1000 to convert from 1000 US$ to US$.
$EV(E)_{ac}$	Export Value (ETJ)	1000 US$	Total value (US$) of trade export of each ETJ feedstock of each country.<br><br>Multiplied by 1000 to convert from 1000 US$ to US$.
JFP_e	Jet Fuel Price	US$/bbl	Jet fuel price derived from crude oil price based on the market trend.<br><br>NOTE:<br><br>Obtained by multiplying crude oil price (in US$/bbl) with 1.21682489655049. Refer to the economic model (Excel Workbook) for more information. Divided by 158.987294928 to convert from per barrel of oil to per litre.
FBR_PrP_{cde}	Filtered Blending Ratio _ Profitable Potential	%	Filtered total blending ratio with the allowable maximum blending ratio regulated by ASTM D7566 – 19 (50%), under different COP / JFP and PC condition.
JFC_c	Jet Fuel Consumption	1000 tonnes	Amount of jet fuel consumed by each country.<br><br>Multiplied by 1×10^6 to convert from 1000 tonnes to kilogram.
JD	Jet Fuel Density	kg/L	Density of jet fuel at a temperature of 15 °C.

7) Total Number of Available Feedstock _ Natural Resource Economics [TNAF_NRE]

Intensified:

$$TNAF\_I_c = \sum NAF(O)\_I_{ac} + \sum NAF(E)\_I_{ac} + \sum NAF(G)\_I_{ac}$$

Harvested:

$$TNAF\_H_c = \sum NAF(O)\_H_{ac} + \sum NAF(E)\_H_{ac} + \sum NAF(G)\_H_{ac}$$

Potential:

$$TNAF\_P_c = \sum NAF(O)\_P_{ac} + \sum NAF(E)\_P_{ac} + \sum NAF(G)\_P_{ac}$$

Profitable Potential:

$$TNAF\_PrP_{cde} = \sum NAF(O)\_PrP_{acde} + \sum NAF(E)\_PrP_{acde} + \sum NAF(G)\_PrP_{acde}$$

Abbreviation	Name	Units	Description
TNAF_NRE	Total Number of Available Feedstock _ Natural Resource Economics	-	Total number of available feedstocks (including 3 production pathways) to produce bio-jet fuel under each NRE scenario for each country.<br><br>NOTE:<br><br>Maximum total number of available feedstocks is 39.
NAF(O)_NRE	Number of Available Feedstock (OTJ) _ Natural Resource Economics	-	Number of available OTJ feedstocks available to produce bio-jet fuel under different NRE scenarios.<br><br>NOTE:<br><br>Maximum number of available OTJ feedstocks is 16.
NAF(E)_NRE	Number of Available Feedstock (ETJ) _ Natural Resource Economics	-	Number of available ETJ feedstocks available to produce bio-jet fuel under different NRE scenarios.<br><br>NOTE:<br><br>Maximum number of available ETJ feedstocks is 12.
NAF(G)_NRE	Number of Available Feedstock (GTJ) _ Natural Resource Economics	-	Number of available GTJ wastes available to produce bio-jet fuel under different NRE scenarios.<br><br>NOTE:<br><br>Maximum number of available GTJ wastes is 11.

E) Energy Security – Energy Use (NRE)

1) Current Bio-Jet Fuel Production in Energy [$CBPE_c$]

Potential & Profitable Potential:

$$CBPE_c = CBP_c \times BEC \times BD$$

Abbreviation	Name	Units	Description
$CBPE_c$	Current Bio-Jet Fuel Production in Energy	MJ	Current bio-jet fuel production energy content for each country in real life.
CBP_c	Current Bio-Jet Fuel Production	L	Current bio-jet fuel production for each country in real life.
BEC	Bio-Jet Fuel Energy Content	MJ/kg	Energy content (lower heating value) of bio-jet fuel.
BD	Bio-Jet Fuel Density	kg/L	Density of bio-jet fuel at a temperature of 15 °C.

2) Total Bio-Jet Fuel Production in Energy _ Natural Resource Economics [TBPE_NRE]

Intensified:

$$TBPE\_I_c = TBP\_I_c \times BEC \times BD$$

Harvested:

$$TBPE\_H_c = TBP\_H_c \times BEC \times BD$$

Potential:

$$TBPE\_P_c = TBP\_P_c \times BEC \times BD$$

Profitable Potential:

$$TBPE\_PrP_{cde} = TBP\_PrP_{cde} \times BEC \times BD$$

Abbreviation	Name	Units	Description
TBPE_NRE	Total Bio-Jet Fuel Production in Energy _ Natural Resource Economics	MJ	Total amount of bio-jet fuel energy content that can be produced under different NRE scenarios.
TBP_NRE	Total Bio-Jet Fuel Production _ Natural Resource Economics	L	Total bio-jet fuel production volume under different NRE scenarios.
BEC	Bio-Jet Fuel Energy Content	MJ/kg	Energy content (lower heating value) of bio-jet fuel.
BD	Bio-Jet Fuel Density	kg/L	Density of bio-jet fuel at a temperature of 15 °C.

3) Jet Fuel Consumption in Energy [$JFCE_c$]

$$JFCE_c = (JFC_c \times 10^6) \times JEC$$

Abbreviation	Name	Units	Description
$JFCE_c$	Jet Fuel Consumption in Energy	MJ	Amount of jet fuel used by every country (in terms of energy).
JFC_c	Jet Fuel Consumption	1000 tonnes	Amount of jet fuel consumed by each country.<br><br>Multiplied by 1×10^6 to convert from 1000 tonnes to kilogram.
JEC	Jet Fuel Energy Content	MJ/kg	Energy content (lower heating value) of jet fuel.

4) Biofuel & Waste Energy _ Natural Resource Economics [BWE_NRE]

Intensified:

$$BWE\_I_c = TBPE\_I_c + [ES(biofuels\ and\ waste)_c \times 41\,868\,000]$$

Harvested:

$$BWE\_H_c = TBPE\_H_c + [ES(biofuels\ and\ waste)_c \times 41\,868\,000]$$

Potential:

$$BWE\_P_c = TBPE\_P_c + [ES(biofuels\ and\ waste)_c \times 41\,868\,000]$$

Profitable Potential:

$$BWE\_PrP_{cde} = TBPE\_PrP_{cde} + [ES(biofuels\ and\ waste)_c \times 41\,868\,000]$$

Abbreviation	Name	Units	Description
BWE_NRE	Biofuel & Waste Energy _ Natural Resource Economics	MJ	Energy content of biofuels and waste energy sector of each country under different NRE scenarios.
TBPE_NRE	Total Bio-Jet Fuel Production in Energy _ Natural Resource Economics	MJ	Total amount of bio-jet fuel energy content that can be produced under different NRE scenarios.
ES(biofuels and waste)$_c$	Energy Source (Biofuels and Waste)	ktoe	Energy of biofuels and waste sector of each country.<br><br>Multiplied by 41868000 to convert from kilotonnes of oil equivalent to megajoules.

5) Total Primary Energy Supply _ Natural Resource Economics [TPES_NRE]

Intensified:

$$TPES\_I_c = [ES(coal)_c + ES(crude\ oil)_c + ES(oil\ products)_c + ES(natural\ gas)_c + ES(nuclear)_c + ES(hydro)_c + ES(wind, solar, etc.)_c] \times 41868000 + BWE\_I_c$$

Harvested:

$$TPES\_H_c = [ES(coal)_c + ES(crude\ oil)_c + ES(oil\ products)_c + ES(natural\ gas)_c + ES(nuclear)_c + ES(hydro)_c + ES(wind, solar, etc.)_c] \times 41868000 + BWE\_H_c$$

Potential:

$$TPES\_P_c = [ES(coal)_c + ES(crude\ oil)_c + ES(oil\ products)_c + ES(natural\ gas)_c + ES(nuclear)_c + ES(hydro)_c + ES(wind, solar, etc.)_c] \times 41868000 + BWE\_P_c$$

Profitable Potential:

$$TPES\_PrP_{cde} = [ES(coal)_c + ES(crude\ oil)_c + ES(oil\ products)_c + ES(natural\ gas)_c + ES(nuclear)_c + ES(hydro)_c + ES(wind, solar, etc.)_c] \times 41868000 + BWE\_PrP_{cde}$$

Abbreviation	Name	Units	Description
TPES_NRE	Total Primary Energy Supply _ Natural Resource Economics	MJ	Sum of primary energy supply after accounting for the bio-jet fuel produced under each NRE scenario for every country.
$ES(coal)_c$	Energy Source (Coal)	ktoe	Energy of coal sector of each country.<br><br>Multiplied by 41868000 to convert from kilotonnes of oil equivalent to megajoules.
$ES(crude\ oil)_c$	Energy Source (Crude Oil)	ktoe	Energy of crude oil sector of each country.<br><br>Multiplied by 41868000 to convert from kilotonnes of oil equivalent to megajoules.
$ES(oil\ products)_c$	Energy Source (Oil Products)	ktoe	Energy of oil products sector of each country.<br><br>Multiplied by 41868000 to convert from kilotonnes of oil equivalent to megajoules.
$ES(natural\ gas)_c$	Energy Source (Natural Gas)	ktoe	Energy of natural gas sector of each country.<br><br>Multiplied by 41868000 to convert from kilotonnes of oil equivalent to megajoules.
$ES(nuclear)_c$	Energy Source (Nuclear)	ktoe	Energy of nuclear sector of each country.<br><br>Multiplied by 41868000 to convert from kilotonnes of oil equivalent to megajoules.
$ES(hydro)_c$	Energy Source (Hydro)	ktoe	Energy of hydro-powered sector of each country.<br><br>Multiplied by 41868000 to convert from kilotonnes of oil equivalent to megajoules.
$ES(wind,solar,etc.)_c$	Energy Source (Wind,solar,etc.)	ktoe	Energy of renewable (wind, solar, etc.) sector of each country.<br><br>Multiplied by 41868000 to convert from kilotonnes of oil equivalent to megajoules.
BWE_NRE	Biofuel & Waste Energy _ Natural Resource Economics	MJ	Energy content of biofuels and waste energy sector of each country under different NRE scenarios.

6) Energy Use per Capita _ Natural Resource Economics [EUC_NRE]

Intensified:

$$EUC\_I_c = \frac{TPES\_I_c - \left(JFCE_c \times \frac{FBR\_I_c}{100}\right)}{Population_c \times 1000}$$

Harvested:

$$EUC\_H_c = \frac{TPES\_H_c - \left(JFCE_c \times \frac{FBR\_H_c}{100}\right)}{Population_c \times 1000}$$

Potential:

$$EUC\_P_c = \frac{TPES\_P_c - \left(JFCE_c \times \frac{FBR\_P_c}{100}\right)}{Population_c \times 1000}$$

Profitable Potential:

$$EUC\_PrP_{cde} = \frac{TPES\_PrP_{cde} - \left(JFCE_c \times \frac{FBR\_PrP_{cde}}{100}\right)}{Population_c \times 1000}$$

Abbreviation	Name	Units	Description
EUC_NRE	Energy Use per Capita _ Natural Resource Economics	MJ/capita	Amount of energy used for each person under each NRE scenario for every country.
TPES_NRE	Total Primary Energy Supply _ Natural Resource Economics	MJ	Sum of primary energy supply after accounting for the bio-jet fuel produced under each NRE scenario for every country.
$JFCE_c$	Jet Fuel Consumption in Energy	MJ	Amount of jet fuel used by every country (in terms of energy).
FBR_NRE	Filtered Blending Ratio _ Natural Resource Economics	%	Filtered total blending ratio with the allowable maximum blending ratio regulated by ASTM D7566 – 19 (50%), under different NRE scenarios.
$Population_c$	Total Population	1000 pax	Total population of each country.<br><br>Multiplied by 1000 to convert from 1000 pax to pax.

7) Biofuel Percentage _ Natural Resource Economics [BPCT_NRE]

Intensified:

$$BPCT\_I_c = \frac{BWE\_I_c}{TPES\_I_c - \left(JFCE_c \times \frac{FBR\_I_c}{100}\right)} \times 100\%$$

Harvested:

$$BPCT\_H_c = \frac{BWE\_H_c}{TPES\_H_c - \left(JFCE_c \times \frac{FBR\_H_c}{100}\right)} \times 100\%$$

Potential:

$$BPCT\_P_c = \frac{BWE\_P_c}{TPES\_P_c - \left(JFCE_c \times \frac{FBR\_P_c}{100}\right)} \times 100\%$$

Profitable Potential:

$$BPCT\_PrP_{cde} = \frac{BWE\_PrP_{cde}}{TPES\_PrP_{cde} - \left(JFCE_c \times \frac{FBR\_PrP_{cde}}{100}\right)} \times 100\%$$

Abbreviation	Name	Units	Description
BPCT_NRE	Biofuel Percentage _ Natural Resource Economics	%	Percentage of biofuels and waste energy sector to the total energy used by each country under different NRE scenarios.
TPES_NRE	Total Primary Energy Supply _ Natural Resource Economics	MJ	Sum of primary energy supply after accounting for the bio-jet fuel produced under each NRE scenario for every country.
BWE_NRE	Biofuel & Waste Energy _ Natural Resource Economics	MJ	Energy content of biofuels and waste energy sector of each country under different NRE scenarios.
$JFCE_c$	Jet Fuel Consumption in Energy	MJ	Amount of jet fuel used by every country (in terms of energy).
FBR_NRE	Filtered Blending Ratio _ Natural Resource Economics	%	Filtered total blending ratio with the allowable maximum blending ratio regulated by ASTM D7566 – 19 (50%), under different NRE scenarios.

8) Difference in Biofuel Percentage _ Natural Resource Economics [DBPCT_NRE]

Intensified:

$$DBPCT\_I_c = BPCT\_I_c - \left(\frac{ES(biofuels\ and\ waste)_c \times 41\,868\,000}{TPES\_I_c - TBPE\_I_c} \times 100\%\right)$$

Harvested:

$$DBPCT\_H_c = BPCT\_H_c - \left(\frac{ES(biofuels\ and\ waste)_c \times 41\,868\,000}{TPES\_H_c - TBPE\_H_c} \times 100\%\right)$$

Potential:

$$DBPCT\_P_c = BPCT\_P_c - \left(\frac{ES(biofuels\ and\ waste)_c \times 41\,868\,000}{TPES\_P_c - TBPE\_P_c} \times 100\%\right)$$

Profitable Potential:

$$DBPCT\_PrP_{cde} = BPCT\_PrP_{cde} - \left(\frac{ES(biofuels\ and\ waste)_c \times 41\,868\,000}{TPES\_PrP_{cde} - TBPE\_PrP_{cde}} \times 100\%\right)$$

Abbreviation	Name	Units	Description
DBPCT_NRE	Difference in Biofuel Percentage _ Natural Resource Economics	%	Percentage difference to the original biofuels and waste energy sector after taking bio-jet fuel production of each country into account, under different NRE scenarios.
BPCT_NRE	Biofuel Percentage _ Natural Resource Economics	%	Percentage of biofuels and waste energy sector to the total energy used by each country under different NRE scenarios.
ES(biofuels and waste)$_c$	Energy Source (Biofuels and Waste)	ktoe	Energy of biofuels and waste sector of each country.<br><br>Multiplied by 41868000 to convert from kilotonnes of oil equivalent to megajoules.
TPES_NRE	Total Primary Energy Supply _ Natural Resource Economics	MJ	Sum of primary energy supply after accounting for the bio-jet fuel produced under each NRE scenario for every country.
TBPE_NRE	Total Bio-Jet Fuel Production in Energy _ Natural Resource Economics	MJ	Total amount of bio-jet fuel energy content that can be produced under different NRE scenarios.

9) Bio-Jet Fuel Shares in Energy _ Natural Resource Economics [BSE_NRE]

Intensified:

$$BSE\_I_c = \frac{TBPE\_I_c}{TPES\_I_c - \left(JFCE_c \times \frac{FBR\_I_c}{100}\right)} \times 100\%$$

Harvested:

$$BSE\_H_c = \frac{TBPE\_H_c}{TPES\_H_c - \left(JFCE_c \times \frac{FBR\_H_c}{100}\right)} \times 100\%$$

Potential:

$$BSE\_P_c = \frac{(TBPE\_P_c + CBPE_c)}{TPES\_P_c - \left(JFCE_c \times \frac{FBR\_P_c}{100}\right)} \times 100\%$$

Profitable Potential:

$$BSE\_PrP_{cde} = \frac{(TBPE\_PrP_{cde} + CBPE_c)}{TPES\_PrP_{cde} - \left(JFCE_c \times \frac{FBR\_PrP_{cde}}{100}\right)} \times 100\%$$

Abbreviation	Name	Units	Description
BSE_NRE	Bio-Jet Fuel Shares in Energy _ Natural Resource Economics	%	Percentage of bio-jet fuel to the total energy used by each country under each NRE scenario.
TPES_NRE	Total Primary Energy Supply _ Natural Resource Economics	MJ	Sum of primary energy supply after accounting for the bio-jet fuel produced under each NRE scenario for every country.
TBPE_NRE	Total Bio-Jet Fuel Production in Energy _ Natural Resource Economics	MJ	Total amount of bio-jet fuel energy content that can be produced under different NRE scenarios.
$CBPE_c$	Current Bio-Jet Fuel Production in Energy	MJ	Current bio-jet fuel production energy content for each country in real life.
$JFCE_c$	Jet Fuel Consumption in Energy	MJ	Amount of jet fuel used by every country (in terms of energy).
FBR_NRE	Filtered Blending Ratio _ Natural Resource Economics	%	Filtered total blending ratio with the allowable maximum blending ratio regulated by ASTM D7566 – 19 (50%), under different NRE scenarios.

10) Renewable Shares in Energy _ Natural Resource Economics [RSE_NRE]

Intensified:

$$RSE\_I_c = \frac{BWE\_I_c + [ES(wind, solar, etc.)_c \; + ES(hydro)_c] \times 41868000}{TPES\_I_c - \left(JFCE_c \times \frac{FBR\_I_c}{100}\right)} \times 100\%$$

Harvested:

$$RSE\_H_c = \frac{BWE\_H_c + [ES(wind, solar, etc.)_c \; + ES(hydro)_c] \times 41868000}{TPES\_H_c - \left(JFCE_c \times \frac{FBR\_H_c}{100}\right)} \times 100\%$$

Potential:

$$RSE\_P_c = \frac{BWE\_P_c + [ES(wind, solar, etc.)_c \; + ES(hydro)_c] \times 41868000}{TPES\_P_c - \left(JFCE_c \times \frac{FBR\_P_c}{100}\right)} \times 100\%$$

Profitable Potential:

$$RSE\_PrP_{cde} = \frac{BWE\_PrP_{cde} + [ES(wind, solar, etc.)_c \; + ES(hydro)_c] \times 41868000}{TPES\_PrP_{cde} - \left(JFCE_c \times \frac{FBR\_PrP_{cde}}{100}\right)} \times 100\%$$

Abbreviation	Name	Units	Description
RSE_NRE	Renewable Shares in Energy _ Natural Resource Economics	%	Percentage of renewable energy after considering the energy content of bio-jet fuel produced into total primary energy source of a country under different NRE scenarios.
BWE_NRE	Biofuel & Waste Energy _ Natural Resource Economics	MJ	Energy content of biofuels and waste energy sector of each country under different NRE scenarios.
$ES(hydro)_c$	Energy Source (Hydro)	ktoe	Energy of hydro-powered sector of each country.<br><br>Multiplied by 41868000 to convert from kilotonnes of oil equivalent to megajoules.
$ES(wind,solar,etc.)_c$	Energy Source (Wind,solar,etc.)	ktoe	Energy of renewable (wind, solar, etc.) sector of each country.<br><br>Multiplied by 41868000 to convert from kilotonnes of oil equivalent to megajoules.
TPES_NRE	Total Primary Energy Supply _ Natural Resource Economics	MJ	Sum of primary energy supply after accounting for the bio-jet fuel produced under each NRE scenario for every country.
$JFCE_c$	Jet Fuel Consumption in Energy	MJ	Amount of jet fuel used by every country (in terms of energy).
FBR_NRE	Filtered Blending Ratio _ Natural Resource Economics	%	Filtered total blending ratio with the allowable maximum blending ratio regulated by ASTM D7566 – 19 (50%), under different NRE scenarios.

11) Current Renewable Shares in Energy _ Natural Resource Economics [CRSE_NRE]

Intensified:

$$CRSE\_I_c = \frac{[ES(biofuels\ and\ waste)_c + ES(wind\ , solar, etc.)_c\ +\ ES(hydro)_c] \times 41868000}{TPES\_I_c - TBPE\_I_c} \times 100\%$$

Harvested:

$$CRSE\_H_c = \frac{[ES(biofuels\ and\ waste)_c + ES(wind\ , solar, etc.)_c\ +\ ES(hydro)_c] \times 41868000}{TPES\_H_c - TBPE\_H_c} \times 100\%$$

Potential:

$$CRSE\_P_c = \frac{[ES(biofuels\ and\ waste)_c + ES(wind\ , solar, etc.)_c\ +\ ES(hydro)_c] \times 41868000}{TPES\_P_c - TBPE\_P_c} \times 100\%$$

Profitable Potential:

$$CRSE\_PrP_{cde} = \frac{[ES(biofuels\ and\ waste)_c + ES(wind\ , solar, etc.)_c\ +\ ES(hydro)_c] \times 41868000}{TPES\_PrP_{cde} - TBPE\_PrP_{cde}} \times 100\%$$

Abbreviation	Name	Units	Description
CRSE_NRE	Current Renewable Shares in Energy _ Natural Resource Economics	%	The original percentage of renewable energy in a country without considering the energy content of bio-jet fuel under different NRE scenarios.
ES(biofuels and waste)$_c$	Energy Source (Biofuels and Waste)	ktoe	Energy of biofuels and waste sector of each country.<br><br>Multiplied by 41868000 to convert from kilotonnes of oil equivalent to megajoules.
ES(hydro)$_c$	Energy Source (Hydro)	ktoe	Energy of hydro-powered sector of each country.<br><br>Multiplied by 41868000 to convert from kilotonnes of oil equivalent to megajoules.
ES(wind,solar,etc.)$_c$	Energy Source (Wind,solar,etc.)	ktoe	Energy of renewable (wind, solar, etc.) sector of each country.<br><br>Multiplied by 41868000 to convert from kilotonnes of oil equivalent to megajoules.
TPES_NRE	Total Primary Energy Supply _ Natural Resource Economics	MJ	Sum of primary energy supply after accounting for the bio-jet fuel produced under each NRE scenario for every country.
TBPE_NRE	Total Bio-Jet Fuel Production in Energy _ Natural Resource Economics	MJ	Total amount of bio-jet fuel energy content that can be produced under different NRE scenarios.

12) Difference in Renewable Shares in Energy _ Natural Resource Economics [DRSE_NRE]

Intensified:

$$DRSE\_I_c = RSE\_I_c - CRSE\_I_c$$

Harvested:

$$DRSE\_H_c = RSE\_H_c - CRSE\_H_c$$

Potential:

$$DRSE\_P_c = RSE\_P_c - CRSE\_P_c$$

Profitable Potential:

$$DRSE\_PrP_{cde} = RSE\_PrP_{cde} - CRSE\_PrP_{cde}$$

Abbreviation	Name	Units	Description
DRSE_NRE	Difference in Renewable Shares in Energy _ Natural Resource Economics	%	Percentage change of renewable energy compared to current renewable energy content of each country under different NRE scenarios.
RSE_NRE	Renewable Shares in Energy _ Natural Resource Economics	%	Percentage of renewable energy after considering the energy content of bio-jet fuel produced into total primary energy source of a country under different NRE scenarios.
CRSE_NRE	Current Renewable Shares in Energy _ Natural Resource Economics	%	The original percentage of renewable energy in a country without considering the energy content of bio-jet fuel under different NRE scenarios.

13) Herfindahl-Hirschman Index _ Natural Resource Economics [HHI_NRE]

Intensified:

$$HHI\_I_c = \frac{ES(coal)_c^{\,2} + ES(crude\ oil)_c^{\,2} + \left[ES(oil\ products)_c - \left(\frac{JFCE_c}{41868000} \times \frac{FBR\_I_c}{100}\right)\right]^2 + ES(natural\ gas)_c^{\,2} + ES(nuclear)_c^{\,2} + ES(hydro)_c^{\,2} + ES(wind, solar, etc.)_c^{\,2} + \left(\frac{BWE\_I_c}{41868000}\right)^2}{\left[\frac{TPES\_I_c}{41868000} - \left(\frac{JFCE_c}{41868000} \times \frac{FBR\_I_c}{100}\right)\right]^2}$$

Harvested:

$$HHI\_H_c = \frac{ES(coal)_c^{\,2} + ES(crude\ oil)_c^{\,2} + \left[ES(oil\ products)_c - \left(\frac{JFCE_c}{41868000} \times \frac{FBR\_H_c}{100}\right)\right]^2 + ES(natural\ gas)_c^{\,2} + ES(nuclear)_c^{\,2} + ES(hydro)_c^{\,2} + ES(wind, solar, etc.)_c^{\,2} + \left(\frac{BWE\_H_c}{41868000}\right)^2}{\left[\frac{TPES\_H_c}{41868000} - \left(\frac{JFCE_c}{41868000} \times \frac{FBR\_H_c}{100}\right)\right]^2}$$

Potential:

$$HHI\_P_c = \frac{ES(coal)_c^{\,2} + ES(crude\ oil)_c^{\,2} + \left[ES(oil\ products)_c - \left(\frac{JFCE_c}{41868000} \times \frac{FBR\_P_c}{100}\right)\right]^2 + ES(natural\ gas)_c^{\,2} + ES(nuclear)_c^{\,2} + ES(hydro)_c^{\,2} + ES(wind, solar, etc.)_c^{\,2} + \left(\frac{BWE\_P_c}{41868000}\right)^2}{\left[\frac{TPES\_P_c}{41868000} - \left(\frac{JFCE_c}{41868000} \times \frac{FBR\_P_c}{100}\right)\right]^2}$$

Profitable Potential:

$$HHI\_PrP_{cde} = \frac{ES(coal)_c^{\,2} + ES(crude\ oil)_c^{\,2} + \left[ES(oil\ products)_c - \left(\frac{JFCE_c}{41868000} \times \frac{FBR\_PrP_{cde}}{100}\right)\right]^2 + ES(natural\ gas)_c^{\,2} + ES(nuclear)_c^{\,2} + ES(hydro)_c^{\,2} + ES(wind, solar, etc.)_c^{\,2} + \left(\frac{BWE\_PrP_{cde}}{41868000}\right)^2}{\left[\frac{TPES\_PrP_{cde}}{41868000} - \left(\frac{JFCE_c}{41868000} \times \frac{FBR\_PrP_{cde}}{100}\right)\right]^2}$$

Abbreviation	Name	Units	Description
HHI_NRE	Herfindahl-Hirschman Index _ Natural Resource Economics	-	Herfindahl-Hirschman index of each country under different NRE scenarios.<br><br>NOTE:<br><br>High HHI means higher shares of bio-jet fuel energy in each country, thus lowers the competition and increases the saturation in the market, and vice versa.
$ES(coal)_c$	Energy Source (Coal)	ktoe	Energy of coal sector of each country.
$ES(crude\ oil)_c$	Energy Source (Crude Oil)	ktoe	Energy of crude oil sector of each country.
$ES(oil\ products)_c$	Energy Source (Oil Products)	ktoe	Energy of oil products sector of each country.
$ES(natural\ gas)_c$	Energy Source (Natural Gas)	ktoe	Energy of natural gas sector of each country.
$ES(nuclear)_c$	Energy Source (Nuclear)	ktoe	Energy of nuclear sector of each country.
$ES(hydro)_c$	Energy Source (Hydro)	ktoe	Energy of hydro-powered sector of each country.
$ES(wind,solar,etc.)_c$	Energy Source (Wind,solar,etc.)	ktoe	Energy of renewable (wind, solar, etc.) sector of each country.
BWE_NRE	Biofuel & Waste Energy _ Natural Resource Economics	MJ	Energy content of biofuels and waste energy sector of each country under different NRE scenarios.<br><br>Divided by 41868000 to convert from megajoules to kilotonnes of oil equivalent.
TPES_NRE	Total Primary Energy Supply _ Natural Resource Economics	MJ	Sum of primary energy supply after accounting for the bio-jet fuel produced under each NRE scenario for every country.<br><br>Divided by 41868000 to convert from megajoules to kilotonnes of oil equivalent.
$JFCE_c$	Jet Fuel Consumption in Energy	MJ	Amount of jet fuel used by every country (in terms of energy).<br><br>Divided by 41868000 to convert from megajoules to kilotonnes of oil equivalent.
FBR_NRE	Filtered Blending Ratio _ Natural Resource Economics	%	Filtered total blending ratio with the allowable maximum blending ratio regulated by ASTM D7566 – 19 (50%), under different NRE scenarios.

14) New Jet Fuel Import Volume _ Natural Resource Economics [NJIV_NRE]

Intensified:

$$NJIV\_I_c = \frac{(JFI_c \times 10^6)}{JD} - (TBP\_I_c - TBEV\_I_c)$$

Harvested:

$$NJIV\_H_c = \frac{(JFI_c \times 10^6)}{JD} - (TBP\_H_c - TBEV\_H_c)$$

Potential:

$$NJIV\_P_c = \frac{(JFI_c \times 10^6)}{JD} - (TBP\_P_c + CBP_c - TBEV\_P_c)$$

Profitable Potential:

$$NJIV\_PrP_{cde} = \frac{(JFI_c \times 10^6)}{JD} - (TBP\_PrP_{cde} + CBP_c - TBEV\_PrP_{cde})$$

Abbreviation	Name	Units	Description
NJIV_NRE	New Jet Fuel Import Volume _ Natural Resource Economics	L	New jet fuel import volume when bio-jet fuel is produced from the feedstocks of each country under different NRE scenarios.
JFI_c	Jet Fuel Import	1000 tonnes	Current jet fuel import quantity of each country.<br><br>Multiplied by 1×10^6 to convert from 1000 tonnes to kilogram.
TBP_NRE	Total Bio-Jet Fuel Production _ Natural Resource Economics	L	Total bio-jet fuel production volume under different NRE scenarios.
TBEV_NRE	Total Bio-Jet Fuel Export Volume _ Natural Resource Economics	L	Volume of bio-jet fuel that can be exported if the total blending ratio exceeds the maximum blending ratio allowed by ASTM D7566 – 19 (50%), depending on each NRE scenario.
CBP_c	Current Bio-Jet Fuel Production	L	Current bio-jet fuel production for each country in real life.
JD	Jet Fuel Density	kg/L	Density of jet fuel at a temperature of 15 °C.

15) Current Jet Fuel Import Dependency [$CJFID_c$]

$$CJFID_c = \frac{JFI_c}{JFC_c} \times 100\%$$

Abbreviation	Name	Units	Description
$CJFID_c$	Current Jet Fuel Import Dependency	%	Percentage of jet fuel import dependency of each country in real life.
JFI_c	Jet Fuel Import	1000 tonnes	Current jet fuel import quantity of each country.
JFC_c	Jet Fuel Consumption	1000 tonnes	Amount of jet fuel consumed by each country.

16) Import Dependency _ Natural Resource Economics [ID_NRE]

Intensified:

$$ID\_I_c = \frac{NJIV\_I_c \times JD}{(JFC_c \times 10^6)} \times 100\%$$

Harvested:

$$ID\_H_c = \frac{NJIV\_H_c \times JD}{(JFC_c \times 10^6)} \times 100\%$$

Potential:

$$ID\_P_c = \frac{NJIV\_P_c \times JD}{(JFC_c \times 10^6)} \times 100\%$$

Profitable Potential:

$$ID\_PrP_{cde} = \frac{NJIV\_PrP_{cde} \times JD}{(JFC_c \times 10^6)} \times 100\%$$

Abbreviation	Name	Units	Description
ID_NRE	Import Dependency _ Natural Resource Economics	%	Jet fuel import dependency after taking the production of bio-jet fuel from the feedstocks of each country into account, under different NRE scenarios.
NJIV_NRE	New Jet Fuel Import Volume _ Natural Resource Economics	L	New jet fuel import volume when bio-jet fuel is produced from the feedstocks of each country under different NRE scenarios.
JFC_c	Jet Fuel Consumption	1000 tonnes	Amount of jet fuel consumed by each country.<br><br>Multiplied by 1×10^6 to convert from 1000 tonnes to kilogram.
JD	Jet Fuel Density	kg/L	Density of jet fuel at a temperature of 15 °C.

[Future Use]

Intensified:

$$ID\_I_c = \frac{BIV_c - \left(BEV_c + \frac{TBP\_I_c \times BD}{1 \times 10^6}\right)}{BIV_c - BEV_c + BP_c} \times 100\%$$

Harvested:

$$ID\_H_c = \frac{BIV_c - \left(BEV_c + \frac{TBP\_H_c \times BD}{1 \times 10^6}\right)}{BIV_c - BEV_c + BP_c} \times 100\%$$

Potential:

$$ID\_P_c = \frac{BIV_c - \left(BEV_c + \frac{TBP\_P_c \times BD}{1 \times 10^6} + BP_c\right)}{BIV_c - BEV_c + BP_c} \times 100\%$$

Profitable Potential:

$$ID\_PrP_{cde} = \frac{BIV_c - \left(BEV_c + \frac{TBP\_PrP_{cde} \times BD}{1 \times 10^6} + BP_c\right)}{BIV_c - BEV_c + BP_c} \times 100\%$$

Abbreviation	Name	Units	Description
ID_NRE	Import Dependency _ Natural Resource Economics	%	Jet fuel import dependency after taking the production of bio-jet fuel from the feedstocks of each country into account, under different NRE scenarios.
BIV_c	Bio-jet Fuel Import	1000 tonnes	Import volume of pure bio-jet fuel for each country.
BEV_c	Bio-jet Fuel Export	1000 tonnes	Export volume of pure bio-jet fuel for each country.
TBP_NRE	Total Bio-Jet Fuel Production _ Natural Resource Economics	L	Total bio-jet fuel production volume under different NRE scenarios.<br><br>Multiplied by 1×10^{-6} to convert from kilogram to 1000 tonnes
BD	Bio-Jet Fuel Density	kg/L	Density of bio-jet fuel at a temperature of 15 °C.
BP_c	Current Bio-Jet Fuel Production	1000 tonnes	Current bio-jet fuel production volume in each country in real life.

F) Energy Security – Environmental Emissions (NRE)

1) Jet Fuel Emission [JFE_{cf}]

$$JFE_{cf} = (JFC_c \times 10^6) \times \frac{JFEI_f}{1000}$$

Abbreviation	Name	Units	Description
JFE_{cf}	Jet Fuel Emission	kg	Amount of emission of NO_x, CO, UHC or soot by jet fuel in each country.
JFC_c	Jet Fuel Consumption	1000 tonnes	Amount of jet fuel consumed by each country.<br><br>Multiplied by 1×10^6 to convert from 1000 tonnes to kilogram.
$JFEI_f$	Jet Fuel Emission Index	g/kg	Emission indices of jet fuel for each type of post-combustion gaseous molecules (NO_x, CO, UHC or soot).<br><br>Multiplied by 0.001 to convert from gram to kilogram.

2) Bio-Jet Fuel Emission _ Natural Resource Economics [BE_NRE]

Intensified:

$$BE\_I_{cf} = (TBP\_I_c \times BD) \times \frac{BEI_f}{1000}$$

Harvested:

$$BE\_H_{cf} = (TBP\_H_c \times BD) \times \frac{BEI_f}{1000}$$

Potential:

$$BE\_P_{cf} = [(TBP\_P_c + CBP_c) \times BD] \times \frac{BEI_f}{1000}$$

Profitable Potential:

$$BE\_PrP_{cdef} = [(TBP\_PrP_{cde} + CBP_c) \times BD] \times \frac{BEI_f}{1000}$$

Abbreviation	Name	Units	Description
BE_NRE	Bio-Jet Fuel Emission _ Natural Resource Economics	kg	Amount of emission of NO_x, CO, UHC or soot by bio-jet fuel in each country under different NRE scenarios.
TBP_NRE	Total Bio-Jet Fuel Production _ Natural Resource Economics	L	Total bio-jet fuel production volume under different NRE scenarios.
CBP_c	Current Bio-Jet Fuel Production	L	Current bio-jet fuel production for each country in real life.
BEI_f	Bio-Jet Fuel Emission Index	g/kg	Emission indices of bio-jet fuel for each type of post-combustion gaseous molecules (NO_x, CO, UHC or soot).<br><br>Multiplied by 0.001 to convert from gram to kilogram.
JD	Jet Fuel Density	kg/L	Density of jet fuel at a temperature of 15 °C.

3) Emission Reduction _ Natural Resource Economics [ER_NRE]

Intensified:

$$ER\_I_{cf} = \left[JFE_{cf} \times \left(\frac{FBR\_I_c}{100}\right)\right] - BE\_I_{cf}$$

Harvested:

$$ER\_H_{cf} = \left[JFE_{cf} \times \left(\frac{FBR\_H_c}{100}\right)\right] - BE\_H_{cf}$$

Potential:

$$ER\_P_{cf} = \left[JFE_{cf} \times \left(\frac{FBR\_P_c}{100}\right)\right] - BE\_P_{cf}$$

Profitable Potential:

$$ER\_PrP_{cdef} = \left[JFE_{cf} \times \left(\frac{FBR\_PrP_{cde}}{100}\right)\right] - BE\_PrP_{cdef}$$

Abbreviation	Name	Units	Description
ER_NRE	Emission Reduction _ Natural Resource Economics	kg	Amount of NO_x, CO, UHC or soot emissions reduced if bio-jet fuel is produced in each country under different NRE scenarios.
JFE_{cf}	Jet Fuel Emission	kg	Amount of emission of NO_x, CO, UHC or soot by jet fuel in each country.
FBR_NRE	Filtered Blending Ratio _ Natural Resource Economics	%	Filtered total blending ratio with the allowable maximum blending ratio regulated by ASTM D7566 – 19 (50%), under different NRE scenarios.
BE_NRE	Bio-Jet Fuel Emission _ Natural Resource Economics	kg	Amount of emission of NO_x, CO, UHC or soot by bio-jet fuel in each country under different NRE scenarios.

4) Percentage Emission Reduction _ Natural Resource Economics [PER_NRE]

Intensified:

$$PER\_I_{cf} = \frac{ER\_I_{cf}}{JFE_{cf}} \times 100\%$$

Harvested:

$$PER\_H_{cf} = \frac{ER\_H_{cf}}{JFE_{cf}} \times 100\%$$

Potential:

$$PER\_P_{cf} = \frac{ER\_P_{cf}}{JFE_{cf}} \times 100\%$$

Profitable Potential:

$$PER\_PrP_{cdef} = \frac{ER\_PrP_{cdef}}{JFE_{cf}} \times 100\%$$

Abbreviation	Name	Units	Description
PER_NRE	Percentage Emission Reduction _ Natural Resource Economics	%	Percentage reduction of NO_x, CO, UHC or soot emission if bio-jet fuel is produced in each country under different NRE scenarios.
ER_NRE	Emission Reduction _ Natural Resource Economics	kg	Amount of NO_x, CO, UHC or soot emissions reduced if bio-jet fuel is produced in each country under different NRE scenarios.
JFE_{cf}	Jet Fuel Emission	kg	Amount of emission of NO_x, CO, UHC or soot by jet fuel in each country.

5) Domestic Plantation Area [DPA_c]

$$DPA_c = \max[DPA(O)_{abc}, DPA(E)_{abc}, DPA(G)_{abc}]$$

Abbreviation	Name	Units	Description
DPA_c	Domestic Plantation Area	ha	Plantation area of the dominant feedstock amongst each production pathway.<br><br>NOTE:<br><br>To prevent any overlapping of the crops' plantation area.
$DPA(O)_{abc}$	Domestic Plantation Area (OTJ)	ha	Current OTJ domestic feedstock plantation area for each country (with OTJ dominant feedstock).
$DPA(E)_{abc}$	Domestic Plantation Area (ETJ)	ha	Current ETJ domestic feedstock plantation area for each country (with ETJ dominant feedstock).
$DPA(G)_{abc}$	Domestic Plantation Area (GTJ)	ha	Current GTJ domestic feedstock plantation area for each country (with GTJ dominant feedstock).

6) Total Plantation Area _ Natural Resource Economics [TPA_NRE]

Intensified:

$$TPA\_I_c = \sum PA(O)\_I_{abc} + \sum PA(E)\_I_{abc} + \sum PA(G)\_I_{abc}$$

Harvested:

$$TPA\_H_c = \sum PA(O)\_H_{abc} + \sum PA(E)\_H_{abc} + \sum PA(G)\_H_{abc}$$

Potential:

$$TPA\_P_c = \sum PA(O)\_P_{abc} + \sum PA(E)\_P_{abc} + \sum PA(G)\_P_{abc}$$

Profitable Potential:

$$TPA\_PrP_{cde} = \sum PA(O)\_PrP_{abcde} + \sum PA(E)\_PrP_{abcde} + \sum PA(G)\_PrP_{abcde}$$

Abbreviation	Name	Units	Description
TPA_NRE	Total Plantation Area _ Natural Resource Economics	ha	Total plantation area of feedstock required by each country in each NRE scenario.
PA(O)_NRE	Plantation Area (OTJ) _ Natural Resource Economics	ha	Plantation area required to produce bio-jet fuel under different NRE scenarios for each OTJ feedstock in each country.
PA(E)_NRE	Plantation Area (ETJ) _ Natural Resource Economics	ha	Plantation area required to produce bio-jet fuel under different NRE scenarios for each ETJ feedstock in each country.
PA(G)_NRE	Plantation Area (GTJ) _ Natural Resource Economics	ha	Plantation area required to produce bio-jet fuel under different NRE scenarios for each GTJ waste in each country.

7) Land Use Emission _ Natural Resource Economics [LUEm_NRE]

Intensified:

$$LUEm\_I_c = \frac{TPA\_I_c}{TAL_c \times 1000} \times (CNEm_c \times 10^6)$$

Harvested:

$$LUEm\_H_c = \frac{TPA\_H_c}{TAL_c \times 1000} \times (CNEm_c \times 10^6)$$

Potential:

$$LUEm\_P_c = \frac{(TPA\_P_c + DPA_c)}{TAL_c \times 1000} \times (CNEm_c \times 10^6)$$

Profitable Potential:

$$LUEm\_PrP_{cde} = \frac{(TPA\_PrP_{cde} + DPA_c)}{TAL_c \times 1000} \times (CNEm_c \times 10^6)$$

Abbreviation	Name	Units	Description
LUEm_NRE	Land Use Emission _ Natural Resource Economics	kg CO_2eq	Amount of GHG emission due to the usage (ILUC) of the total plantation area of each country under different NRE scenarios.
TPA_NRE	Total Plantation Area _ Natural Resource Economics	ha	Total plantation area of feedstock required by each country in each NRE scenario.
DPA_c	Domestic Plantation Area	ha	Plantation area of the dominant feedstock amongst each production pathway.<br><br>NOTE:<br><br>To prevent any overlapping of the crops' plantation area.
TAL_c	Total Agricultural Land	1000 ha	Sum of areas for arable land, permanent crops and permanent pastures for agricultural purposes for each country.<br><br>Multiplied by 1000 to convert from 1000 hectares to hectares.
$CNEm_c$	Cropland Net Emissions	CO_2eq gigagrams	Net emissions in CO_2eq for all cropland (for agricultural purposes) of each country.<br><br>Multiplied by 1×10^6 to convert from gigagram to kilogram.

8) Fertiliser Use Emission _ Natural Resource Economics [FertUEm_NRE]

Intensified:

$$FertUEm\_I_c = TPA\_I_c \times AvgN_c \times (SynNFEm_c \times 10^6)$$

Harvested:

$$FertUEm\_H_c = TPA\_H_c \times AvgN_c \times (SynNFEm_c \times 10^6)$$

Potential:

$$FertUEm\_P_c = (TPA\_P_c + DPA_c) \times AvgN_c \times (SynNFEm_c \times 10^6)$$

Profitable Potential:

$$FertUEm\_PrP_{cde} = (TPA\_PrP_{cde} + DPA_c) \times AvgN_c \times (SynNFEm_c \times 10^6)$$

Abbreviation	Name	Units	Description
FertUEm_NRE	Fertiliser Use Emission _ Natural Resource Economics	kg CO_2eq	Amount of GHG emission due to the usage of nitrogenous fertiliser on the total plantation area of each country under different NRE scenarios.
TPA_NRE	Total Plantation Area _ Natural Resource Economics	ha	Total plantation area of feedstock required by each country in each NRE scenario.
DPA_c	Domestic Plantation Area	ha	Plantation area of the dominant feedstock amongst each production pathway.<br><br>NOTE:<br><br>To prevent any overlapping of the crops' plantation area.
$AvgN_c$	Average Nitrogen N Use per Area of Cropland	kg/ha	Average nitrogen N used per cropland area for each country.
$SynNFEm_c$	Total Synthetic Nitrogen Fertilizers Emissions	CO_2eq gigagrams	Total emissions from the use of nitrogenous fertilisers in each country.<br><br>Multiplied by 1×10^6 to convert from gigagram to kilogram.

9) GHG Emission _ Natural Resource Economics [GHGEm_NRE]

Intensified:

$$GHGEm\_I_c = LUEm\_I_c + FertUEm\_I_c$$

Harvested:

$$GHGEm\_H_c = LUEm\_H_c + FertUEm\_H_c$$

Potential:

$$GHGEm\_P_c = LUEm\_P_c + FertUEm\_P_c$$

Profitable Potential:

$$GHGEm\_PrP_{cde} = LUEm\_PrP_{cde} + FertUEm\_PrP_{cde}$$

Abbreviation	Name	Units	Description
GHGEm_NRE	GHG Emission _ Natural Resource Economics	kg CO_2eq	Total amount of GHG emission due to bio-jet fuel production under different NRE scenarios.
LUEm_NRE	Land Use Emission _ Natural Resource Economics	kg CO_2eq	Amount of GHG emission due to the usage (ILUC) of the total plantation area of each country under different NRE scenarios.
FertUEm_NRE	Fertiliser Use Emission _ Natural Resource Economics	kg CO_2eq	Amount of GHG emission due to the usage of nitrogenous fertiliser on the total plantation area of each country under different NRE scenarios.

G) Water Security – Water Stress (NRE)

1) Baseline Water Stress Index [$BWSI_c$]

$$BWSI_c = \frac{AWW_c}{TRWR}$$

Abbreviation	Name	Units	Description
$BWSI_c$	Baseline Water Stress Index	-	A measure of the amount of pressure exerted to natural water resources due to agricultural activity in each country. It is the total annual water withdrawals (consider agricultural only, not municipal and industrial) expressed as a percent of the total annual available flow (renewable supply).<br><br>NOTE:<br><br>Higher values indicate more competition among users.
AWW_c	Agricultural Water Withdrawal	10^9 m^3/year	Total agricultural water withdrawal of each country.<br><br>Multiplied by 1×10^9 to convert from 10^9 cubic metre to cubic metre.
$TRWR_c$	Total Renewable Water Resources	10^9 m^3/year	Total renewable water resources of each country.<br><br>Multiplied by 1×10^9 to convert from 10^9 cubic metre to cubic metre.

2) Total Crop Water Required _ Natural Resource Economics [TCWR_NRE]

Intensified:

$$TCWR\_I_c = \sum CWR(O)\_I_{abc} + \sum CWR(E)\_I_{abc}$$

Harvested:

$$TCWR\_H_c = \sum CWR(O)\_H_{abc} + \sum CWR(E)\_H_{abc}$$

Potential:

$$TCWR\_P_c = \sum CWR(O)\_P_{abc} + \sum CWR(E)\_P_{abc}$$

Profitable Potential:

$$TCWR\_PrP_{cde} = \sum CWR(O)\_PrP_{abcde} + \sum CWR(E)\_PrP_{abcde}$$

Abbreviation	Name	Units	Description
TCWR_NRE	Total Crop Water Required _ Natural Resource Economics	m^3	Total volume of water required to produce the quantity of each crop or feedstock for bio-jet fuel production in each country under different NRE scenarios.
CWR(O)_NRE	Crop Water Required (OTJ) _ Natural Resource Economics	m^3	Water required to produce each OTJ feedstock for bio-jet fuel production in each country under different NRE scenarios.
CWR(E)_NRE	Crop Water Required (ETJ) _ Natural Resource Economics	m^3	Water required to produce each ETJ feedstock for bio-jet fuel production in each country under different NRE scenarios.

3) Total Water Required _ Natural Resource Economics [TWR_NRE]

Intensified:

$$TWR\_I_c = \sum WRP(O)\_I_{abc} + \sum WRP(G)\_I_{abc} + \sum WRP(G)\_I_{abc} + (TCWR\_I_c \times 1000)$$

Harvested:

$$TWR\_H_c = \sum WRP(O)\_H_{abc} + \sum WRP(G)\_H_{abc} + \sum WRP(G)\_H_{abc} + (TCWR\_H_c \times 1000)$$

Potential:

$$TWR\_P_c = \sum WRP(O)\_P_{abc} + \sum WRP(G)\_P_{abc} + \sum WRP(G)\_P_{abc} + (TCWR\_P_c \times 1000)$$

Profitable Potential:

$$TWR\_PrP_{cde} = \sum WRP(O)\_PrP_{abcde} + \sum WRP(G)\_PrP_{abcde} + \sum WRP(G)\_PrP_{abcde} + (TCWR\_PrP_{cde} \times 1000)$$

Abbreviation	Name	Units	Description
TWR_NRE	Total Water Required _ Natural Resource Economics	L	Total amount of water required to produce bio-jet fuel (from scratch) in each country under different NRE scenarios.
WRP(O)_NRE	Water Required for Production (OTJ) _ Natural Resource Economics	L	Total amount of water required for OTJ process to produce bio-jet fuel for each country under different NRE scenarios.
WRP(E)_NRE	Water Required for Production (ETJ) _ Natural Resource Economics	L	Total amount of water required for ETJ process to produce bio-jet fuel for each country under different NRE scenarios.
WRP(G)_NRE	Water Required for Production (GTJ) _ Natural Resource Economics	L	Total amount of water required for GTJ process to produce bio-jet fuel for each country under different NRE scenarios.
TCWR_NRE	Total Crop Water Required _ Natural Resource Economics	m^3	Total volume of water required to produce the quantity of each crop or feedstock for bio-jet fuel production in each country under different NRE scenarios.<br><br>Multiplied by 1000 to convert from cubic metre to litre.

4) Fraction of Water Stress Index _ Natural Resource Economics [FWSI_NRE]

Intensified:

$$FWSI\_I_c = \frac{TWR\_I_c}{(Precipitation_c + TRWR_c) \times 10^{12}}$$

Harvested:

$$FWSI\_H_c = \frac{TWR\_H_c}{(Precipitation_c + TRWR_c) \times 10^{12}}$$

Potential:

$$FWSI\_P_c = \frac{TWR\_P_c}{(Precipitation_c + TRWR_c) \times 10^{12}}$$

Profitable Potential:

$$FWSI\_PrP_{cde} = \frac{TWR\_PrP_{cde}}{(Precipitation_c + TRWR_c) \times 10^{12}}$$

Abbreviation	Name	Units	Description
FWSI_NRE	Fraction of Water Stress Index _ Natural Resource Economics	-	Fraction of contribution to the water stress of each country by their corresponding bio-jet fuel production under different NRE scenarios.
TWR_NRE	Total Water Required _ Natural Resource Economics	L	Total amount of water required to produce bio-jet fuel (from scratch) in each country under different NRE scenarios.
$Precipitation_c$	Long-term Average Annual Precipitation in Volume	10^9 m^3/year	Total rainwater volume of each country yearly.<br><br>Multiplied by 1×10^9 to convert from 10^9 cubic metre to cubic metre.
$TRWR_c$	Total Renewable Water Resources	10^9 m^3/year	Total renewable water resources of each country.<br><br>Multiplied by 1×10^9 to convert from 10^9 cubic metre to cubic metre.

5) Water Withdrawal per Capita _ Natural Resource Economics [WWC_NRE]

Intensified:

$$WWC\_I_c = \frac{TWR\_I_c}{Population_c \times 1000}$$

Harvested:

$$WWC\_H_c = \frac{TWR\_H_c}{Population_c \times 1000}$$

Potential:

$$WWC\_P_c = \frac{\left(\frac{CBP_c}{1000} \times BD\right) \times \sum \frac{TWF_{abc}}{1000} + TWR\_P_c}{Population_c \times 1000}$$

Profitable Potential:

$$WWC\_PrP_{cde} = \frac{\left(\frac{CBP_c}{1000} \times BD\right) \times \sum \left(\frac{TWF_{abc}}{1000}\right) + TWR\_PrP_{cde}}{Population_c \times 1000}$$

Abbreviation	Name	Units	Description
WWC_NRE	Water Withdrawal per Capita _ Natural Resource Economics	L/capita	Amount of water being withdrawn from the society in each country due to bio-jet fuel production under each NRE scenario.
TWR_NRE	Total Water Required _ Natural Resource Economics	L	Total amount of water required to produce bio-jet fuel (from scratch) in each country under different NRE scenarios.
TWF_{abc}	Total Water Footprint	m^3/tonne	Total water required per ton of crop / feedstock production for each country.<br><br>Multiplied by 0.001 to convert from tonnes to kilogram under intensified scenario only.
$Population_c$	Total Population	1000 pax	Total population of each country.<br><br>Multiplied by 1000 to convert from 1000 pax to pax.
CBP_c	Current Bio-Jet Fuel Production	L	Current bio-jet fuel production for each country in real life.
BD	Bio-Jet Fuel Density	kg/L	Density of bio-jet fuel at a temperature of 15 °C.

6) Total Green Water Footprint _ Natural Resource Economics [TGNWF_NRE]

Intensified:

$$TGNWF\_I_c = TBP\_I_c \times BD \times \sum\left(\frac{GNWF_{abc}}{1000}\right)$$

Harvested:

$$TGNWF\_H_c = TBP\_H_c \times BD \times \sum\left(\frac{GNWF_{abc}}{1000}\right)$$

Potential:

$$TGNWF\_P_c = (CBP_c + TBP\_P_c) \times BD \times \sum\left(\frac{GNWF_{abc}}{1000}\right)$$

Profitable Potential:

$$TGNWF\_PrP_{cde} = (CBP_c + TBP\_PrP_{cde}) \times BD \times \sum\left(\frac{GNWF_{abc}}{1000}\right)$$

Abbreviation	Name	Units	Description
TGNWF_NRE	Total Green Water Footprint _ Natural Resource Economics	m^3	Total amount of rainwater used for bio-jet fuel production of each country under each NRE scenario.
TBP_NRE	Total Bio-Jet Fuel Production _ Natural Resource Economics	L	Total bio-jet fuel production volume under different NRE scenarios.
CBP_c	Current Bio-Jet Fuel Production	L	Current bio-jet fuel production for each country in real life.
$GNWF_{abc}$	Green Water Footprint	m^3/tonne	Water precipitation (rainwater) required per ton of crop / feedstock production for each country.<br><br>Multiplied by 0.001 to convert from tonnes to kilogram.
BD	Bio-Jet Fuel Density	kg/L	Density of bio-jet fuel at a temperature of 15 °C.

7) Total Blue Water Footprint _ Natural Resource Economics [TBLWF_NRE]

Intensified:

$$TBLWF\_I_c = TBP\_I_c \times BD \times \sum \left(\frac{BLWF_{abc}}{1000}\right)$$

Harvested:

$$TBLWF\_H_c = TBP\_H_c \times BD \times \sum \left(\frac{BLWF_{abc}}{1000}\right)$$

Potential:

$$TBLWF\_P_c = (CBP_c + TBP\_P_c) \times BD \times \sum \left(\frac{BLWF_{abc}}{1000}\right)$$

Profitable Potential:

$$TBLWF\_PrP_{cde} = (CBP_c + TBP\_PrP_{cde}) \times BD \times \sum \left(\frac{BLWF_{abc}}{1000}\right)$$

Abbreviation	Name	Units	Description
TBLWF_NRE	Total Blue Water Footprint _ Natural Resource Economics	m^3	Total amount of irrigation water used for bio-jet fuel production of each country under each NRE scenario.
TBP_NRE	Total Bio-Jet Fuel Production _ Natural Resource Economics	L	Total bio-jet fuel production volume under different NRE scenarios.
CBP_c	Current Bio-Jet Fuel Production	L	Current bio-jet fuel production for each country in real life.
$BLWF_{abc}$	Blue Water Footprint	m^3/tonne	Irrigation water used per ton of crop / feedstock production for each country.<br><br>Multiplied by 0.001 to convert from tonnes to kilogram.
BD	Bio-Jet Fuel Density	kg/L	Density of bio-jet fuel at a temperature of 15 °C.

8) Total Grey Water Footprint _ Natural Resource Economics [TGYWF_NRE]

Intensified:

$$TGYWF\_I_c = TBP\_I_c \times BD \times \sum\left(\frac{GYWF_{abc}}{1000}\right)$$

Harvested:

$$TGYWF\_H_c = TBP\_H_c \times BD \times \sum\left(\frac{GYWF_{abc}}{1000}\right)$$

Potential:

$$TGYWF\_P_c = (CBP_c + TBP\_P_c) \times BD \times \sum\left(\frac{GYWF_{abc}}{1000}\right)$$

Profitable Potential:

$$TGYWF\_PrP_{cde} = (CBP_c + TBP\_PrP_{cde}) \times BD \times \sum\left(\frac{GYWF_{abc}}{1000}\right)$$

Abbreviation	Name	Units	Description
TGYWF_NRE	Total Grey Water Footprint _ Natural Resource Economics	m^3	Total amount of fresh and groundwater used for bio-jet fuel production of each country under each NRE scenario.
TBP_NRE	Total Bio-Jet Fuel Production _ Natural Resource Economics	L	Total bio-jet fuel production volume under different NRE scenarios.
CBP_c	Current Bio-Jet Fuel Production	L	Current bio-jet fuel production for each country in real life.
$GYWF_{abc}$	Grey Water Footprint	m^3/tonne	Fresh water required to dilute pollution caused per ton of crop / feedstock production for each country.<br><br>Multiplied by 0.001 to convert from tonnes to kilogram.
BD	Bio-Jet Fuel Density	kg/L	Density of bio-jet fuel at a temperature of 15 °C.

H) Water Security – Agriculture (NRE)

1) Green Water Scarcity Index _ Natural Resource Economics [GNWSI_NRE]

Intensified:

$$GNWSI\_I_c = \frac{TGNWF\_I_c}{Precipitation_c \times 10^9}$$

Harvested:

$$GNWSI\_H_c = \frac{TGNWF\_H_c}{Precipitation_c \times 10^9}$$

Potential:

$$GNWSI\_P_c = \frac{TGNWF\_P_c}{Precipitation_c \times 10^9}$$

Profitable Potential:

$$GNWSI\_PrP_{cde} = \frac{TGNWF\_PrP_{cde}}{Precipitation_c \times 10^9}$$

Abbreviation	Name	Units	Description
GNWSI_NRE	Green Water Scarcity Index _ Natural Resource Economics	-	Rainwater insufficiency due to the total green water footprint for bio-jet fuel production in each country under each NRE scenario.
TGNWF_NRE	Total Green Water Footprint _ Natural Resource Economics	m^3	Total amount of rainwater used for bio-jet fuel production of each country under each NRE scenario.
$Precipitation_c$	Long-term Average Annual Precipitation in Volume	10^9 m^3/year	Total rainwater volume of each country yearly.<br><br>Multiplied by 1×10^9 to convert from 10^9 cubic metre to cubic metre.

2) Blue Water Scarcity Index _ Natural Resource Economics [BLWSI_NRE]

Intensified:

$$BLWSI\_I_c = \frac{TBLWF\_I_c}{TRWR_c \times 10^9}$$

Harvested:

$$BLWSI\_H_c = \frac{TBLWF\_H_c}{TRWR_c \times 10^9}$$

Potential:

$$BLWSI\_P_c = \frac{TBLWF\_P_c}{TRWR_c \times 10^9}$$

Profitable Potential:

$$BLWSI\_PrP_{cde} = \frac{TBLWF\_PrP_{cde}}{TRWR_c \times 10^9}$$

Abbreviation	Name	Units	Description
BLWSI_NRE	Blue Water Scarcity Index _ Natural Resource Economics	-	Irrigation water insufficiency due to the total blue water footprint for bio-jet fuel production in each country under each NRE scenario.
TBLWF_NRE	Total Blue Water Footprint _ Natural Resource Economics	m^3	Total amount of irrigation water used for bio-jet fuel production of each country under each NRE scenario.
$TRWR_c$	Total Renewable Water Resources	10^9 m^3/year	Total renewable water resources of each country.<br><br>Multiplied by 1×10^9 to convert from 10^9 cubic metre to cubic metre.

3) Grey Water Scarcity Index _ Natural Resource Economics [GYWSI_NRE]

Intensified:

$$GYWSI\_I_c = \frac{TGYWF\_I_c}{(Precipitation_c + TRWR_c) \times 10^9}$$

Harvested:

$$GYWSI\_H_c = \frac{TGYWF\_H_c}{(Precipitation_c + TRWR_c) \times 10^9}$$

Potential:

$$GYWSI\_P_c = \frac{TGYWF\_P_c}{(Precipitation_c + TRWR_c) \times 10^9}$$

Profitable Potential:

$$GYWSI\_PrP_{cde} = \frac{TGYWF\_PrP_{cde}}{(Precipitation_c + TRWR_c) \times 10^9}$$

Abbreviation	Name	Units	Description
GYWSI_NRE	Grey Water Scarcity Index _ Natural Resource Economics	-	Fresh and groundwater insufficiency due to the total grey water footprint for bio-jet fuel production in each country under each NRE scenario.
TGYWF_NRE	Total Grey Water Footprint _ Natural Resource Economics	m^3	Total amount of fresh and groundwater used for bio-jet fuel production of each country under each NRE scenario.
$Precipitation_c$	Long-term Average Annual Precipitation in Volume	10^9 m^3/year	Total rainwater volume of each country yearly.<br><br>Multiplied by 1×10^9 to convert from 10^9 cubic metre to cubic metre.
$TRWR_c$	Total Renewable Water Resources	10^9 m^3/year	Total renewable water resources of each country.<br><br>Multiplied by 1×10^9 to convert from 10^9 cubic metre to cubic metre.

4) Agricultural Dependency _ Natural Resource Economics [AD_NRE]

Intensified:

$$AD\_I_c = \frac{TWR\_I_c}{AWW_c \times 10^{12}} \times 100\%$$

Harvested:

$$AD\_H_c = \frac{TWR\_H_c}{AWW_c \times 10^{12}} \times 100\%$$

Potential:

$$AD\_P_c = \frac{(CBP_c \times BD) \times \sum \frac{TWF_{abc}}{1000} + \frac{TWR\_P_c}{1000}}{AWW_c \times 10^9} \times 100\%$$

Profitable Potential:

$$AD\_PrP_{cde} = \frac{(CBP_c \times BD) \times \sum \frac{TWF_{abc}}{1000} + \frac{TWR\_PrP_{cde}}{1000}}{AWW_c \times 10^9} \times 100\%$$

Abbreviation	Name	Units	Description
AD_NRE	Agricultural Dependency _ Natural Resource Economics	%	Dependency of water used in Bio-jet fuel production to agricultural water usage for each NRE scenario.
TWR_NRE	Total Water Required _ Natural Resource Economics	L	Total amount of water required to produce bio-jet fuel (from scratch) in each country under different NRE scenarios.<br><br>Multiplied by 0.001 to convert from litre to cubic metre for potential and profitable potential scenarios only.
AWW_c	Agricultural Water Withdrawal	10^9 m^3/year	Total agricultural water withdrawal of each country.<br><br>Multiplied by 1×10^{12} to convert from 10^9 cubic metre to litre for intensified and harvested scenarios only.<br><br>Multiplied by 1×10^9 to convert from 10^9 cubic metre to cubic metre for potential and profitable potential scenarios only.
CBP_c	Current Bio-Jet Fuel Production	L	Current bio-jet fuel production for each country in real life.
BD	Bio-Jet Fuel Density	kg/L	Density of bio-jet fuel at a temperature of 15 °C.
TWF_{abc}	Total Water Footprint	m^3/tonne	Total water required per ton of crop / feedstock production for each country.<br><br>Multiplied by 0.001 to convert from tonnes to kilogram.

I) Food Security – Availability (NRE)

1) Cropland per Capita _ Natural Resource Economics [CLC_NRE]

Intensified:

$$CLC\_I_c = \frac{TPA\_I_c}{Population_c \times 1000}$$

Harvested:

$$CLC\_H_c = \frac{TPA\_H_c}{Population_c \times 1000}$$

Potential:

$$CLC\_P_c = \frac{TPA\_P_c + DPA_c}{Population_c \times 1000}$$

Profitable Potential:

$$CLC\_PrP_{cde} = \frac{TPA\_PrP_{cde} + DPA_c}{Population_c \times 1000}$$

Abbreviation	Name	Units	Description
CLC_NRE	Cropland per Capita _ Natural Resource Economics	ha/capita	Total area of cropland used by the society in each country for bio-jet fuel production under different NRE scenarios.
TPA_NRE	Total Plantation Area _ Natural Resource Economics	ha	Total plantation area of feedstock required by each country in each NRE scenario.
DPA_c	Domestic Plantation Area	ha	Plantation area of the dominant feedstock amongst each production pathway.<br><br>NOTE:<br><br>To prevent any overlapping of the crops' plantation area.
$Population_c$	Total Population	1000 pax	Total population of each country.<br><br>Multiplied by 1000 to convert from 1000 pax to pax.

2) Bio-Jet Fuel Production for Crops _ Natural Resource Economics [BPC_NRE]

Intensified:

$$BPC\_I_{bc} = BP(O)\_I_{abc} + BP(E)\_I_{abc} + BP(G)\_I_{abc}$$

Harvested:

$$BPC\_H_{bc} = BP(O)\_H_{abc} + BP(E)\_H_{abc} + BP(G)\_H_{abc}$$

Potential:

$$BPC\_P_{bc} = BP(O)\_P_{abc} + BP(E)\_P_{abc} + BP(G)\_P_{abc}$$

Profitable Potential:

$$BPC\_PrP_{bcde} = BP(O)\_PrP_{abcde} + BP(E)\_PrP_{abcde} + BP(G)\_PrP_{abcde}$$

Abbreviation	Name	Units	Description
BPC_NRE	Bio-Jet Fuel Production for Crops _ Natural Resource Economics	L	Volume of bio-jet fuel produced from each corresponding crop through OTJ, ETJ and GTJ processes in each country under different NRE scenarios.<br><br>NOTE:<br><br>• Be careful especially when calculating across OTJ, ETJ & GTJ processes that share the same corresponding crops, as follows: Barley, Maize, Oats, Oil Palm Fruit, Rice, paddy, and Wheat. Other corresponding crops do not repeat across OTJ, ETJ and GTJ processes.
BP(O)_NRE	Bio-Jet Fuel Production (OTJ) _ Natural Resource Economics	L	Bio-jet fuel production volume from OTJ process under different NRE scenarios.
BP(E)_NRE	Bio-Jet Fuel Production (ETJ) _ Natural Resource Economics	L	Bio-jet fuel production volume from ETJ process under different NRE scenarios.
BP(G)_NRE	Bio-Jet Fuel Production (GTJ) _ Natural Resource Economics	L	Bio-jet fuel production volume from GTJ process under different NRE scenarios.

3) Crop Yield _ Natural Resource Economics [CY_NRE]

Intensified:

$$\begin{aligned}
&If\ \max[CY(O)_{bc}] \neq 0,\\
&CY_I_b = \max[CY(O)_{bc}],\\
&else\ if\ \max[CY(E)_{bc}] \neq 0,\\
&CY_I_b = \max[CY(E)_{bc}],\\
&else\ if\ \max[CY(G)_{bc}] \neq 0,\\
&CY_I_b = \max[CY(G)_{bc}],\\
&else\ leave\ it\ blank.
\end{aligned}$$

Harvested:

$$\begin{aligned}
&If\ PQ(O)_{bc}\ or\ AH(O)_{bc} \neq 0,\\
&CY_H_{bc} = CY(O)_{bc},\\
&else\ if\ PQ(E)_{bc}\ or\ AH(E)_{bc} \neq 0,
\end{aligned}$$

$$CY\_H_{bc} = CY(E)_{bc},$$
$$else\ if\ PQ(G)_{bc}\ or\ AH(G)_{bc} \neq 0,$$
$$CY\_H_{bc} = CY(G)_{bc},$$
$$else\ leave\ it\ blank.$$

Potential:

$$CY\_P_{bc} = \frac{BPC\_P_{bc}}{BPC\_H_{bc}} \times CY\_H_{bc}$$

Abbreviation	Name	Units	Description
CY_NRE	Crop Yield _ Natural Resource Economics	hg/ha	Quantity produced from each hectare of plantation area for each crop in each country under different NRE scenarios.<br><br>NOTE:<br><br>• Values will be the same for all countries under intensified scenario. Be careful especially when calculating across OTJ, ETJ & GTJ processes that share the same corresponding crops, as follows: Barley, Maize, Oats, Oil Palm Fruit, Rice, paddy, and Wheat. Other corresponding crops do not repeat across OTJ, ETJ and GTJ processes.
BPC_NRE_{bc}	Bio-Jet Fuel Production for Crops _ Natural Resource Economics	L	Volume of bio-jet fuel produced from each corresponding crop through OTJ, ETJ and GTJ processes in each country under different NRE scenarios.<br><br>NOTE:<br><br>• Be careful especially when calculating across OTJ, ETJ & GTJ processes that share the same corresponding crops, as follows: Barley, Maize, Oats, Oil Palm Fruit, Rice, paddy, and Wheat. Other corresponding crops do not repeat across OTJ, ETJ and GTJ processes.
$CY(O)_{bc}$	Crop Yield (OTJ)	hg/ha	Quantity produced from each hectare of plantation area for each OTJ crop in each country.
$CY(E)_{bc}$	Crop Yield (ETJ)	hg/ha	Quantity produced from each hectare of plantation area for each ETJ crop in each country.
$CY(G)_{bc}$	Crop Yield (GTJ)	hg/ha	Quantity produced from each hectare of plantation area for each GTJ crop in each country.
$PQ(O)_{bc}$	Production Quantity (OTJ)	tonnes	Production quantity for each OTJ crop in each country.
$PQ(E)_{bc}$	Production Quantity (ETJ)	tonnes	Production quantity for each ETJ crop in each country.
$PQ(G)_{bc}$	Production Quantity (GTJ)	tonnes	Production quantity for each GTJ crop in each country.
$AH(O)_{bc}$	Area Harvested (OTJ)	ha	Total land area used for OTJ crop plantation of each country.
$AH(E)_{bc}$	Area Harvested (ETJ)	ha	Total land area used for ETJ crop plantation of each country.
$AH(G)_{bc}$	Area Harvested (GTJ)	ha	Total land area used for GTJ crop plantation of each country.

4) Total Crop Yield _ Natural Resource Economics [TCY_NRE]

Intensified:

$$TCY\_I_c = \sum CY\_I_b$$

Harvested:

$$TCY\_H_c = \sum CY\_H_{bc}$$

Potential:

$$TCY\_P_c = \sum CY\_P_{bc}$$

Profitable Potential:

$$TCY\_PrP_{cde} = \sum \left(\frac{BPC\_PrP_{bcde}}{BPC\_H_{bc}} \times CY\_H_{bc} \right)$$
$$= \sum \left(\frac{BP(O)\_PrP_{abcde} + BP(E)\_PrP_{abcde} + BP(O)\_PrP_{abcde}}{BP(O)\_H_{abc} + BP(E)\_H_{abc} + BP(G)\_H_{abc}} \times CY\_H_{bc} \right)$$
$$= CY(O)\_PrP_{bcde} + CY(E)\_PrP_{bcde} + CY(G)\_PrP_{bcde}$$

Abbreviation	Name	Units	Description
TCY_NRE	Total Crop Yield _ Natural Resource Economics	hg/ha	Sum of the quantity produced from each hectare of plantation area for each crop in each country under different NRE scenarios.<br><br>NOTE:<br><br>• Values will be the same for all countries under intensified scenario. Be careful especially when calculating across OTJ, ETJ & GTJ processes that share the same corresponding crops, as follows: Barley, Maize, Oats, Oil Palm Fruit, Rice, paddy, and Wheat. Other corresponding crops do not repeat across OTJ, ETJ and GTJ processes.
CY_NRE	Crop Yield _ Natural Resource Economics	hg/ha	Quantity produced from each hectare of plantation area for each crop in each country under different NRE scenarios.<br><br>NOTE:<br><br>Values will be the same for all countries under intensified scenario. Be careful especially when calculating across OTJ, ETJ & GTJ processes that share the same corresponding crops, as follows: Barley, Maize, Oats, Oil Palm Fruit, Rice, paddy, and Wheat. Other corresponding crops do not repeat across OTJ, ETJ and GTJ processes.
$CY(O)\_PrP_{bcde}$	Crop Yield (OTJ) _ Profitable Potential	hg/ha	Quantity produced per hectare of plantation area for each OTJ crops for each country under different COP / JFP and PC condition.
$CY(E)\_PrP_{bcde}$	Crop Yield (ETJ) _ Profitable Potential	hg/ha	Quantity produced per hectare of plantation area for each ETJ crops for each country under different COP / JFP and PC condition.
$CY(G)\_PrP_{bcde}$	Crop Yield (GTJ) _ Profitable Potential	hg/ha	Quantity produced per hectare of plantation area for each GTJ crops for each country under different COP / JFP and PC condition.

5) Volatility of Crop Production _ Natural Resource Economics [VolCP_NRE$_{bc}$]

Intensified:

$$VolCP\_I_{bc} = \frac{\sigma_{PQ\_I_{bc}}}{\mu_{PQ\_I_{bc}}} \times 100\% = VolCPI_{bc}$$

Harvested:

$$VolCP\_H_{bc} = \frac{\sigma_{PQ_{bc}}}{\mu_{PQ_{bc}}} \times 100\% = VolCP_{bc}$$

Potential:

$$If\ BPC\_P_{bc} > 0,$$
$$VolCP\_P_{bc} = VolCP\_H_{bc},$$
$$else\ VolCP\_P_{bc} = 0.$$

Abbreviation	Name	Units	Description
VolCP_NRE$_{bc}$	Volatility of Crop Production _ Natural Resource Economics	%	A measure of the tendency for the production quantity of the 28 crops to vary across 15 years for each country under each NRE scenario.
VolCPI$_{bc}$	Intensified Crop Production Volatility	%	A measure of the tendency for the intensified production quantity of each crop to vary across 15 years in every country.
VolCP$_{bc}$	Crop Production Volatility	%	A measure of the tendency for the production quantity of each crop to vary across 15 years in every country.
BPC_P$_{bc}$	Bio-Jet Fuel Production for Crops _ Potential	L	Volume of bio-jet fuel produced from each corresponding crop through OTJ, ETJ and GTJ processes in each country under potential scenario.<br><br>NOTE:<br><br>• Be careful especially when calculating across OTJ, ETJ & GTJ processes that share the same corresponding crops, as follows: Barley, Maize, Oats, Oil Palm Fruit, Rice, paddy, and Wheat. Other corresponding crops do not repeat across OTJ, ETJ and GTJ processes.

6) Volatility of Agricultural Production _ Natural Resource Economics [VolAP_NRE]

Intensified:

$$VolAP\_I_c = \sum VolCP\_I_{bc}$$

Harvested:

$$VolAP\_H_c = \sum VolCP\_H_{bc}$$

Potential:

$$If\ TBP\_P_c > 0,$$

$$VolAP\_P_c = \sum VolCP\_P_{bc},$$
$$else\ VolAP\_P_c = 0.$$

Profitable Potential:

$$VolAP\_PrP_{cde} = \sum (VolCP\_PrP_{bcde})$$
$$= \sum \left(\frac{BPC\_PrP_{bcde}}{BPC\_P_{bc}} \times VolCP_{bc}\right)$$
$$= \sum \left(\frac{BP(O)\_PrP_{abcde} + BP(E)\_PrP_{abcde} + BP(O)\_PrP_{abcde}}{BP(O)\_H_{abc} + BP(E)\_H_{abc} + BP(G)\_H_{abc}} \times VolCP_{bc}\right)$$
$$= VolCP(O)\_PrP_{bcde} + VolCP(E)\_PrP_{bcde} + VolCP(G)\_PrP_{bcde}$$

Abbreviation	Name	Units	Description
VolAP_NRE	Volatility of Agricultural Production _ Natural Resource Economics	%	Sum of all the tendency for the production quantity of the 28 crops to vary across 15 years for each country under each NRE scenario.
$VolCP\_NRE_{bc}$	Volatility of Crop Production _ Natural Resource Economics	%	A measure of the tendency for the production quantity of the 28 crops to vary across 15 years for each country under each NRE scenario.
TBP_P_c	Total Bio-Jet Fuel Production _ Potential	L	Total bio-jet fuel production volume under potential scenario.
$VolCP(O)\_PrP_{bcde}$	Volatility of Crop Production (OTJ) _ Profitable Potential	%	A measure of the tendency for the production quantity of OTJ crop to vary across 15 years for each country under different COP / JFP and PC condition.
$VolCP(E)\_PrP_{bcde}$	Volatility of Crop Production (ETJ) _ Profitable Potential	%	A measure of the tendency for the production quantity of ETJ crop to vary across 15 years for each country under different COP / JFP and PC condition.
$VolCP(G)\_PrP_{bcde}$	Volatility of Crop Production (GTJ) _ Profitable Potential	%	A measure of the tendency for the production quantity of GTJ crop to vary across 15 years for each country under different COP / JFP and PC condition.

7) Constant Volatility for Crops [constVol_P_{bc}]

Potential:

$$constVol\_P_{bc} = \frac{VolCP\_P_{bc} \times BPC\_P_{bc}}{TPB\_P_c}$$

Abbreviation	Name	Units	Description
constVol_P_{bc}	Constant Volatility for Crops	%	The volatility portions of each crop produced in every country.
VolCP_P_{bc}	Volatility of Crop Production Potential	%	A measure of the tendency for the production quantity of the 28 crops to vary across 15 years for each country under potential scenario.
BPC_P_{bc}	Bio-Jet Fuel Production for Crops Potential	L	Volume of bio-jet fuel produced from each corresponding crop through OTJ, ETJ and GTJ processes in each country under potential scenario.<br><br>NOTE:<br><br>• Be careful especially when calculating across OTJ, ETJ & GTJ processes that share the same corresponding crops, as follows: Barley, Maize, Oats, Oil Palm Fruit, Rice, paddy, and Wheat. Other corresponding crops do not repeat across OTJ, ETJ and GTJ processes.
TBP_P_c	Total Bio-Jet Fuel Production Potential	L	Total bio-jet fuel production volume under potential scenario.

8) Constant Volatility [constVol_P_c]

Potential:

$$constVol\_P_c = \sum (constVol\_P_{bc})$$

Abbreviation	Name	Units	Description
constVol_P_c	Constant Volatility	%	Sum of all the volatility portions of each crop produced in every country.
constVol_P_{bc}	Constant Volatility for Crops	%	The volatility portions of each crop produced in every country.

9) Variable Volatility [varVol_PrP$_{cde}$]

Profitable Potential:

$$varVol\_PrP_{cde} = \sum\left(\frac{VolCP\_PrP_{bcde} \times BPC\_PrP_{bcde}}{TBP\_PrP_{cde}}\right) = \sum\left[\frac{VolCP\_P_{bc} \times (BPC\_PrP_{bcde})^2}{BPC\_P_{bc} \times TBP\_PrP_{cde}}\right]$$

Abbreviation	Name	Units	Description
varVol_PrP$_{cde}$	Variable Volatility	%	Collective volatility of the crops used for bio-jet fuel production under different COP / JFP and PC condition.
VolCP_P$_{bc}$	Volatility of Crop Production _ Potential	%	A measure of the tendency for the production quantity of the 28 crops to vary across 15 years for each country under potential scenario.
BPC_NRE	Bio-Jet Fuel Production for Crops _ Natural Resource Economics	L	Volume of bio-jet fuel produced from each corresponding crop through OTJ, ETJ and GTJ processes in each country under different NRE scenarios.<br><br>NOTE:<br><br>• Be careful especially when calculating across OTJ, ETJ & GTJ processes that share the same corresponding crops, as follows: Barley, Maize, Oats, Oil Palm Fruit, Rice, paddy, and Wheat. Other corresponding crops do not repeat across OTJ, ETJ and GTJ processes.
TBP_PrP$_{cde}$	Total Bio-Jet Fuel Production _ Profitable Potential	L	Total bio-jet fuel production volume under different COP / JFP and PC condition.

10) Total Energy of Feedstock _ Natural Resource Economics [TEF_NRE]

Intensified:

$$TEF\_I_c = \sum EF(O)\_I_{abc} + \sum EF(E)\_I_{abc}$$

Harvested:

$$TEF\_H_c = \sum EF(O)\_H_{abc} + \sum EF(E)\_H_{abc}$$

Potential:

$$TEF\_P_c = \sum EF(O)\_P_{abc} + \sum EF(E)\_P_{abc}$$

Profitable Potential:

$$TEF\_PrP_{cde} = \sum EF(O)\_PrP_{abcde} + \sum EF(E)\_PrP_{abcde}$$

Abbreviation	Name	Units	Description
TEF_NRE	Total Energy of Feedstock _ Natural Resource Economics	MJ	Total energy content of the feedstocks used for bio-jet production in each country under different NRE scenarios.
EF(O)_NRE	Energy of Feedstock (OTJ) _ Natural Resource Economics	MJ	Energy of each OTJ feedstock used by each country under different NRE scenarios.
EF(E)_NRE	Energy of Feedstock (ETJ) _ Natural Resource Economics	MJ	Energy of each ETJ feedstock used by each country under different NRE scenarios.

11) Dietary Energy Supply _ Natural Resource Economics [DES_NRE]

Intensified:

$$DES\_I_c = \frac{TEF\_I_c \times \frac{10000000000}{41868000}}{365 \times (Population_c \times 1000)}$$

Harvested:

$$DES\_H_c = \frac{TEF\_H_c \times \frac{10000000000}{41868000}}{365 \times (Population_c \times 1000)}$$

Potential:

$$DES\_P_c = \frac{TEF\_P_c \times \frac{10000000000}{41868000}}{365 \times (Population_c \times 1000)}$$

Profitable Potential:

$$DES\_PrP_{cde} = \frac{TEF\_PrP_{cde} \times \frac{10000000000}{41868000}}{365 \times (Population_c \times 1000)}$$

Abbreviation	Name	Units	Description
DES_NRE	Dietary Energy Supply _ Natural Resource Economics	kcal/capita/day	Amount of dietary energy required by the society (supplied by total energy of feedstock) under different NRE scenarios.<br><br>NOTE:<br><br>Assuming 365 days per year.
TEF_NRE	Total Energy of Feedstock _ Natural Resource Economics	MJ	Total energy content of the feedstocks used for bio-jet production in each country under different NRE scenarios.<br><br>Multiplied by 1×10^{10} and divided by 41868000 to convert from megajoules to kilocalories.
$Population_c$	Total Population	1000 pax	Total population of each country.<br><br>Multiplied by 1000 to convert from 1000 pax to pax.

12) Average Dietary Energy Supply Adequacy (ADESA) _ Natural Resource Economics [ADESA_NRE]

Intensified:

$$ADESA\_I_c = \frac{DES\_I_c}{ADER_c} \times 100\%$$

Harvested:

$$ADESA\_H_c = \frac{DES\_H_c}{ADER_c} \times 100\%$$

Potential:

$$ADESA\_P_c = \frac{DES\_P_c}{ADER_c} \times 100\%$$

Profitable Potential:

$$ADESA\_PrP_{cde} = \frac{DES\_PrP_{cde}}{ADER_c} \times 100\%$$

Abbreviation	Name	Units	Description
ADESA_NRE	Average Dietary Energy Supply Adequacy (ADESA) _ Natural Resource Economics	%	The adequacy of food supply in terms of calories after accounting bio-jet fuel production in each country under different NRE scenarios.
DES_NRE	Dietary Energy Supply _ Natural Resource Economics	kcal/capita/day	Amount of dietary energy required by the society (supplied by total energy of feedstock) under different NRE scenarios.
$ADER_c$	Average Dietary Energy Requirement	kcal/cap/day	Average caloric intake required to provide energy balance in the population (healthy weights for their genders, ages and activity levels).

13) Fraction of ADESA _ Natural Resource Economics [FADESA_NRE]

Intensified:

$$FADESA\_I_c = \frac{ADESA\_I_c}{100}$$

Harvested:

$$FADESA\_H_c = \frac{ADESA\_H_c}{100}$$

Potential:

$$FADESA\_P_c = \frac{ADESA\_P_c}{100}$$

Profitable Potential:

$$FADESA\_PrP_{cde} = \frac{ADESA\_PrP_{cde}}{100}$$

Abbreviation	Name	Units	Description
FADESA_NRE	Fraction of ADESA _ Natural Resource Economics	-	Fraction of ADESA in each country under different NRE scenarios
ADESA_NRE	Average Dietary Energy Supply Adequacy (ADESA) _ Natural Resource Economics	%	The adequacy of food supply in terms of calories after accounting bio-jet fuel production in each country under different NRE scenarios.

14) New ADESA _ Natural Resource Economics [NADESA_NRE]

Potential:

$$NADESA\_P_c = ADESA_c - ADESA\_P_c$$

Profitable Potential:

$$NADESA\_PrP_{cde} = ADESA_c - ADESA\_PrP_{cde}$$

Abbreviation	Name	Units	Description
NADESA_NRE	New ADESA _ Natural Resource Economics	%	New ADESA after taking feedstocks away from the food market of a country (through import quantities) under different NRE scenarios.
$ADESA_c$	Average Dietary Energy Supply Adequacy	%	Current percentage of Dietary Energy Supply of Average Dietary Energy Requirement, indicating the adequacy of food supply of the country in terms of calories.
ADESA_NRE	Average Dietary Energy Supply Adequacy (ADESA) _ Natural Resource Economics	%	The adequacy of food supply in terms of calories after accounting bio-jet fuel production in each country under different NRE scenarios.

15) Current ADESA _ Natural Resource Economics [CADESA_NRE]

Potential:

$$CADESA\_P_c = \ ADESA_c + ADESA\_P_c$$

Profitable Potential:

$$CADESA\_PrP_{cde} = \ ADESA_c + ADESA\_PrP_{cde}$$

Abbreviation	Name	Units	Description
CADESA_NRE	Current ADESA _ Natural Resource Economics	%	ADESA of the current food market if the feedstocks are used to supply food for the society.
$ADESA_c$	Average Dietary Energy Supply Adequacy	%	Current percentage of Dietary Energy Supply of Average Dietary Energy Requirement, indicating the adequacy of food supply of the country in terms of calories.
ADESA_NRE	Average Dietary Energy Supply Adequacy (ADESA) _ Natural Resource Economics	%	The adequacy of food supply in terms of calories after accounting bio-jet fuel production in each country under different NRE scenarios.

16) Number of People Fed _Natural Resource Economics [NPF_NRE]

Intensified:

$$NPF\_I_c = \frac{TEF\_I_c \times \frac{10000000000}{41868000}}{(365 \times ADER_c)}$$

Harvested:

$$NPF\_H_c = \frac{TEF\_H_c \times \frac{10000000000}{41868000}}{(365 \times ADER_c)}$$

Potential:

$$NPF\_P_c = \frac{TEF\_P_c \times \frac{10000000000}{41868000}}{(365 \times ADER_c)}$$

Profitable Potential:

$$NPF\_PrP_{cde} = \frac{TEF\_PrP_{cde} \times \frac{10000000000}{41868000}}{(365 \times ADER_c)}$$

Abbreviation	Name	Units	Description
NPF_NRE	Number of People Fed _Natural Resource Economics	pax	Number of people fed based on the total calorific values of all feedstocks on the average dietary energy requirement under different NRE scenarios.
TEF_NRE	Total Energy of Feedstock _Natural Resource Economics	MJ	Total energy content of the feedstocks used for bio-jet production in each country under different NRE scenarios.<br><br>Multiplied by 1×10^{10} and divided by 41868000 to convert from megajoules to kilocalories.
$ADER_c$	Average Dietary Energy Requirement	kcal/cap/day	Average caloric intake required to provide energy balance in the population (healthy weights for their genders, ages and activity levels).<br><br>Multiplied by 365 to convert from kilocalories per capita per day to kilocalories per capita.<br><br>NOTE:<br><br>Assuming 365 days per year.

17) Minimum ADESA [$minADESA_c$]

$$minADESA_c = \frac{MDER_c}{ADER_c} \times 100\%$$

Abbreviation	Name	Units	Description
$minADESA_c$	Minimum ADESA	%	Percentage ratio of MDER and ADER of each country.
$MDER_c$	Minimum Dietary Energy Requirement	kcal/cap/day	Average cut-off threshold caloric intake that the population would have to consume to attain their minimum acceptable weight for height, calculated based on the country's demographic weightings.
$ADER_c$	Average Dietary Energy Requirement	kcal/cap/day	Average caloric intake required to provide energy balance in the population (healthy weights for their genders, ages and activity levels).

J) Food Security – Access

1) Depth of Food Deficit [DFD_c]

$$DFD_c = DFD_c$$

Abbreviation	Name	Units	Description
DFD_c	Depth of Food Deficit	kcal/capita/day	Total calories required to lift undernourished from their status, when everything else being constant, in each country.

2) Prevalence of Undernourishment [PU_c]

$$PU_c = PU_c$$

Abbreviation	Name	Units	Description
PU_c	Prevalence of Undernourishment	%	The percentage of total population in each country whose food intake is insufficient to meet dietary energy requirements continuously.

K) Food Security – Stability (NRE)

1) Producer Price Volatility _ Natural Resource Economics [$VolPDP\_NRE_{bc}$]

Intensified:

$$VolPDP\_I_{bc} = \frac{\sigma_{PDP\_I_{bc}}}{\mu_{PDP\_I_{bc}}} \times 100\% = VolPDPI_{bc}$$

Harvested:

$$VolPDP\_H_{bc} = \frac{\sigma_{PDP_{bc}}}{\mu_{PDP_{bc}}} \times 100\% = VolPDP_{bc}$$

Potential:

$$\begin{gathered} If\ BPC\_P_{bc} > 0, \\ VolPCP\_P_{bc} = VolPCP\_H_{bc}, \\ else\ VolDCP\_P_{bc} = 0. \end{gathered}$$

Abbreviation	Name	Units	Description
$VolPDP\_NRE_{bc}$	Producer Price Volatility _ Natural Resource Economics	%	A measure of the tendency for the producer price of each crop to vary across 15 years for each country under different COP / JFP and PC condition.<br><br>NOTE:<br><br>PDP_I_{bc} is the producer price for the country that have intensified crop production, after calculating across 15 years.
$VolPDPI_{bc}$	Intensified Producer Price Volatility	%	A measure of the tendency for the producer price of each crop to vary across 15 years in each country.
$VolPDP_{bc}$	Producer Price Volatility	%	A measure of the tendency for the intensified producer price (defined as having intensified production quantity) of each crop to vary across 15 years in each country.
BPC_P_{bc}	Bio-Jet Fuel Production for Crops _ Potential	L	Volume of bio-jet fuel produced from each corresponding crop through OTJ, ETJ and GTJ processes in each country under potential scenario.<br><br>NOTE:<br><br>• Be careful especially when calculating across OTJ, ETJ & GTJ processes that share the same corresponding crops, as follows: Barley, Maize, Oats, Oil Palm Fruit, Rice, paddy, and Wheat. Other corresponding crops do not repeat across OTJ, ETJ and GTJ processes.

2) Domestic Crop Price Volatility _ Natural Resource Economics [VolDCP_NRE]

Intensified:

$$VolDCP\_I_c = \sum VolPDP\_I_{bc}$$

Harvested:

$$VolDCP\_H_c = \sum VolPDP\_H_{bc}$$

Potential:

$$If\ TBP\_P_c > 0,$$
$$VolDCP\_P_c = \sum VolPDP\_P_{bc},$$
$$else\ VolDCP\_P_c = 0.$$

Profitable Potential:

$$VolDCP\_PrP_{cde} = \sum VolPDP\_PrP_{bcde}$$
$$= \sum \left(\frac{BPC\_PrP_{bcde}}{BPC\_P_{bc}} \times VolPDP_{bc} \right)$$
$$= VolPDP(O)\_PrP_{bcde} + VolPDP(E)\_PrP_{bcde} + VolPDP(G)\_PrP_{bcde}$$

Abbreviation	Name	Units	Description
VolDCP_NRE	Domestic Crop Price Volatility _ Natural Resource Economics	%	Sum of all the tendency for the producer price of the 28 crops to vary across 15 years for each country under each NRE scenario.
$VolPDP\_NRE_{bc}$	Producer Price Volatility _ Natural Resource Economics	%	A measure of the tendency for the producer price of each crop to vary across 15 years for each country under different COP / JFP and PC condition.<br><br>NOTE:<br><br>PDP_I_{bc} is the producer price for the country that have intensified crop production, after calculating across 15 years.
TBP_P_c	Total Bio-Jet Fuel Production _ Potential	L	Total bio-jet fuel production volume under potential scenario.
$VolPDP(O)\_PrP_{bcde}$	Producer Price Volatility (OTJ) _ Profitable Potential	%	A measure of the tendency for the producer price of OTJ crop to vary across 15 years for each country under different COP / JFP and PC condition.
$VolPDP(E)\_PrP_{bcde}$	Producer Price Volatility (ETJ) _ Profitable Potential	%	A measure of the tendency for the producer price of ETJ crop to vary across 15 years for each country under different COP / JFP and PC condition.
$VolPDP(G)\_PrP_{bcde}$	Producer Price Volatility (GTJ) _ Profitable Potential	%	A measure of the tendency for the producer price of GTJ crop to vary across 15 years for each country under different COP / JFP and PC condition.

L) Governance

1) Voice and Accountability [VA_c]

$$VA_c = VA_c$$

Abbreviation	Name	Units	Description
VA_c	Voice and Accountability	%	A measure to reflect perceptions of the extent to which a country's citizens can participate in selecting their government, as well as freedom of expression, freedom of association, and a free media.

2) Political Stability and Absence of Violence [$PSAV_c$]

$$PSAV_c = PSAV_c$$

Abbreviation	Name	Units	Description
$PSAV_c$	Political Stability and Absence of Violence	%	A measure to reflect perceptions of the likelihood of political instability and/or politically motivated violence.

3) Government Effectiveness [GE_c]

$$GE_c = GE_c$$

Abbreviation	Name	Units	Description
GE_c	Government Effectiveness	%	A measure to capture perceptions of the quality of public services, the quality of the civil service and the degree of its independence from political pressures, the quality of policy formulation and implementation, and the credibility of the government's commitment to such policies.

4) Regulatory Quality [RQ_c]

$$RQ_c = RQ_c$$

Abbreviation	Name	Units	Description
RQ_c	Regulatory Quality	%	A measure to capture perceptions of the ability of the government to formulate and implement sound policies and regulations that. permit and promote private sector development.

5) Rule of Law [RL_c]

$$RL_c = RL_c$$

Abbreviation	Name	Units	Description
RL_c	Rule of Law	%	A measure of law constraint and influence towards the behavior of citizen in each country.

6) Control of Corruption [CC_c]

$$CC_c = CC_c$$

Abbreviation	Name	Units	Description
CC_c	Control of Corruption	%	A measure of the public power or bureaucratic regulation exercised for private gain from which it creates corruption in each country, and which may hinder for the foreign investors.

8. Limiting Factor (Profitable Potential)

Supplementary Table 6: Crude Oil Price vs. Blending Ratio (COP vs. BR)

Steps	Descriptions
Concept	**Bio-Jet Fuel Production (Volume) Required at any Blending Ratios** [$xBPRQ_c$]<br><br>For blending ratio, x in the range of 0 to 50 % and intervals of 1, multiply the blending ratio with Current Jet Fuel Consumption [JFC_c] for each country.<br><br>$$xBPRQ_c = \frac{x}{100} \times \left(\frac{JFC_c \times 10^6}{JD}\right), x \in \{0, 1, 2, \dots, 48, 49, 50\}$$
Concept	**Difference in Bio-Jet Fuel Production (Volume)** [dBP_{cde}]<br><br>Subtract [$xBPRQ_c$] with Current Bio-Jet Fuel Production (Volume) [CBP_c] and Total Bio-Jet Fuel Production (Volume) _ Profitable Potential [TBP_PrP_c].<br><br>$$dBP_{cde} = xBPRQ_c - CBP_c - TBP\_PrP_{cde}$$
Concept	**Filtered Difference in Bio-jet Fuel Production (Volume)** [$FdBP_{cde}$]<br><br>A portion of difference in bio-jet fuel production (volume) will remain after the following filter is carried out:<br><br>$$\begin{aligned} &If\ dBP_{cde} > 0, \\ &FdBP_{cde} = dBP_{cde}, \\ &else\ leave\ it\ blank. \end{aligned}$$
Concept	**Maximum Blending Ratio** [$MBR_{COP,cde}$]<br><br>$$MBR_{COP,cde} = BR\ of\ \max[FdBP_{cde}]$$
1	**Derivation for calculating Maximum Blending Ratio** [x_{max}]<br><br>Due to the limitation in terms of the maximum number of rows (1048576) in Microsoft Excel, generating a huge pivot table will be very inconvenient, thus working backwards is the best solution.<br><br>$$\begin{aligned} &x_{max}COP_c\ occurs\ when\ dBP_{cde} = 0, \\ &x_{max}BPRQ_c = CBP_c + TBP\_PrP_{cde}, \\ &\frac{x_{max}}{100} \times \left(\frac{JFC_c \times 10^6}{JD}\right) = CBP_c + TBP\_PrP_{cde}, \\ &x_{max} = \frac{(CBP_c + TBP\_PrP_{cde}) \times 100 \times JD}{JFC_c \times 10^6}. \end{aligned}$$
2	**Maximum Blending Ratio** [x_{max}]<br><br>$$\therefore x_{max} = TBR\_PrP_{cde}.$$

Supplemental Table 7: Water Stress vs. Blending Ratio (WS vs. BR)

Steps	Descriptions
**Concept**	**Proportion of OTJ Contribution to Total Bio-jet Fuel Production _ Profitable Potential** [PPCB(O)$_c$] $$PPCB(O)_c = \frac{BP(O)\_PrP_{cde}}{TBP\_PrP_{cde}}$$ **Proportion of ETJ Contribution to Total Bio-jet Fuel Production _ Profitable Potential** [PPCB(E)$_c$] $$PPCB(E)_c = \frac{BP(E)\_PrP_{cde}}{TBP\_PrP_{cde}}$$ **Proportion of GTJ Contribution to Total Bio-jet Fuel Production _ Profitable Potential** [PPCB(G)$_c$] $$PPCB(G)_c = \frac{BP(G)\_PrP_{cde}}{TBP\_PrP_{cde}}$$
**Concept**	**Bio-jet Fuel Production (OTJ) at any Blending Ratios** [xBFP(O)$_c$] $$xBFP(O)_c = PPCB(O)_c \times xBFP_c$$ **Bio-jet Fuel Production (ETJ) at any Blending Ratios** [xBFP(E)$_c$] $$xBFP(E)_c = PPCB(E)_c \times xBFP_c$$ **Bio-jet Fuel Production (GTJ) at any Blending Ratios** [xBFP(G)$_c$] $$xBFP(G)_c = PPCB(G)_c \times xBFP_c$$
**Concept**	**Water Required for Bio-jet Fuel Production (OTJ) at any Blending Ratios** [WRxBFP(O)$_c$] $$WRxBFP(O)_c = xBFP(O)_c \times WR(O)$$ **Water Required for Bio-jet Fuel Production (ETJ) at any Blending Ratios** [WRxBFP(E)$_c$] $$WRxBFP(E)_c = xBFP(E)_c \times WR(E)$$ **Water Required for Bio-jet Fuel Production (GTJ) at any Blending Ratios** [WRxBFP(G)$_c$] $$WRxBFP(G)_c = xBFP(G)_c \times WR(G)$$
**Concept**	**Total Water Required for Bio-jet Fuel Production at any Blending Ratios** [xTWR$_c$] $$xTWRP_c = WRxBFP(O)_c + WRxBFP(E)_c + WRxBFP(G)_c$$
**Concept**	**Crop Water Required (OTJ) at any Blending Ratios** [CWRxBFP(O)$_c$] $$If\ xBFP(O)_c > 0, \\ CWRxBFP(O)_c = \sum CWR(O)\_PrP_{abc}, \\ else\ CWRxBFP(O)_c = 0.$$ **Crop Water Required (ETJ) at any Blending Ratios** [CWRxBFP(E)$_c$]

	$else\ CWRxBFP(E)_c = 0.$
**Concept**	**Total Crop Water Required at any Blending Ratios** [$xCWR_c$]<br><br>$xTCWR_c = CWRxBFP(O)_c + CWRxBFP(E)_c$
**Concept**	**Fraction of Water Stress Index at any Blending Ratios** [$xFWSI_c$]<br><br>$xFWSI_c = \frac{xTWRP_c + xTCWR_c \times 1000}{(Precipitation_c + TRWR_c) \times 10^{12}}$
**Concept**	**Filtered Fraction of Water Stress Index at any Blending Ratios** [$FxFWSI_c$]<br><br>A portion of fraction of water stress index will remain after the following filter is carried out:<br><br>$If\ xFWSI_c > 0\ and\ xFWSI_c < Water\ Stress\ Limit,$<br>$FxFWSI_c = xFWSI_c,$<br>$else\ leave\ it\ blank.$
**Concept**	**Maximum Blending Ratio** [x_{max}]<br><br>$x_{max} = BR\ of\ \max[FxFWSI_c]$
**1**	**Derivation for calculating Maximum Blending Ratio** [x_{max}]<br><br>Due to the limitation in terms of the maximum number of rows (1048576) in Microsoft Excel, generating a huge pivot table will be very inconvenient, thus working backwards is the best solution.<br><br>Maximum blending ratio will occur when:<br><br>$x_{max}FWSI_c = Water\ Stress\ Limit,$<br>$\frac{x_{max}TWRP_c + x_{max}TCWR_c}{(Precipitation_c + TRWR_c) \times 10^{12}} = 0.032,$<br>$\left(\frac{BP(O)\_PrP_{cde}}{TBP\_PrP_{cde}} \times x_{max}BFP_c \times WR(O)\right) + \left(\frac{BP(E)\_PrP_{cde}}{TBP\_PrP_{cde}} \times x_{max}BFP_c \times WR(E)\right) + \left(\frac{BP(G)\_PrP_{cde}}{TBP\_PrP_{cde}} \times x_{max}BFP_c \times WR(G)\right)$<br>$+ (CWRx_{max}BFP(O)_c + CWRx_{max}BFP(E)_c) \times 1000 = 0.032 \times (Precipitation_c + TRWR_c) \times 10^{12},$<br>$[(BP(O)\_PrP_{cde} \times WR(O)) + (BP(E)\_PrP_{cde} \times WR(E)) + (BP(G)\_PrP_{cde} \times WR(G))] \times \frac{x_{max}BFP_c}{TBP\_PrP_{cde}}$<br>$= 0.032 \times (Precipitation_c + TRWR_c) \times 10^{12} - (CWRx_{max}BFP(O)_c + CWRx_{max}BFP(E)_c) \times 1000,$<br>$x_{max}BFP_c = \frac{[0.032 \times (Precipitation_c + TRWR_c) \times 10^{12} - (CWRx_{max}BFP(O)_c + CWRx_{max}BFP(E)_c) \times 1000] \times TBP\_PrP_{cde}}{[(BP(O)\_PrP_{cde} \times WR(O)) + (BP(E)\_PrP_{cde} \times WR(E)) + (BP(G)\_PrP_{cde} \times WR(G))]},$<br>$\frac{x_{max}}{100} \times \left(\frac{JFC_c \times 10^6}{JD}\right)$<br>$= \frac{[0.032 \times (Precipitation_c + TRWR_c) \times 10^{12} - (CWRx_{max}BFP(O)_c + CWRx_{max}BFP(E)_c) \times 1000] \times TBP\_PrP_{cde}}{[(BP(O)\_PrP_{cde} \times WR(O)) + (BP(E)\_PrP_{cde} \times WR(E)) + (BP(G)\_PrP_{cde} \times WR(G))]},$<br><br>$x_{max} = \frac{[0.032 \times (Precipitation_c + TRWR_c) \times 10^{12} - (CWRx_{max}BFP(O)_c + CWRx_{max}BFP(E)_c) \times 1000] \times TBP\_PrP_{cde} \times 100 \times JD}{[(BP(O)\_PrP_{cde} \times WR(O)) + (BP(E)\_PrP_{cde} \times WR(E)) + (BP(G)\_PrP_{cde} \times WR(G))] \times (JFC_c \times 10^6)}.$
**2**	**Maximum Blending Ratio** [x_{max}]<br><br>$\therefore If\ x_{max} < 0,$<br>$x_{max} = \frac{[0.032 \times (Precipitation_b + TRWR_b) \times 10^{12}] \times TBP\_PrP_{bcd} \times 100 \times JD}{[(BP(O)\_PrP_{bcd} \times WR(O)) + (BP(E)\_PrP_{bcd} \times WR(E)) + (BP(G)\_PrP_{bcd} \times WR(G))] \times (JFC_b \times 10^6)},$<br>$else\ x_{max}$<br>$= \frac{[0.032 \times (Precipitation_b + TRWR_b) \times 10^{12} - (CWRx_{max}BFP(O)_b + CWRx_{max}BFP(E)_b) \times 1000] \times TBP\_PrP_{bcd} \times 100 \times JD}{[(BP(O)\_PrP_{bcd} \times WR(O)) + (BP(E)\_PrP_{bcd} \times WR(E)) + (BP(G)\_PrP_{bcd} \times WR(G))] \times (JFC_b \times 10^6)}.$

Supplementary Table 8: Herfindahl-Hirschman Index vs. Blending Ratio (HHI vs. BR)

<table>
<tr><th>Steps</th><th>Description</th></tr>
<tr><td>1</td><td>

Derivation for calculating Maximum Blending Ratio [$BR_{max,cde}$]

Due to the limitation in terms of the maximum number of rows (1048576) in Microsoft Excel, generating a huge pivot table will be very inconvenient, thus working backwards is the best solution.

By replacing $TBPE\_PrP_{cde}$ and BWE_PrP_{cde},

$$HHI\_PrP_{min,cde} = \frac{\begin{gathered} ES(coal)_c^{\,2} + ES(crude\ oil)_c^{\,2} + \left[ES(oil\ products)_c - \left(\frac{JFCE_c}{41868000} \times \frac{BR_{max,cde}}{100}\right)\right]^2 + ES(natural\ gas)_c^{\,2} + ES(nuclear)_c^{\,2} + ES(hydro)_c^{\,2} + ES(wind, solar, etc.)_c^{\,2} \\ + \left(\left(\frac{JFC_c \times 10^6}{JD \times 100} \times BR_{max,cde} - CBP_c\right) \times \frac{BEC \times BD}{41868000} + ES(biofuels\ and\ waste)_c\right)^2 \end{gathered}}{\left[\begin{gathered} ES(coal)_c + ES(crude\ oil)_c + ES(oil\ products)_c - \left(\frac{JFCE_c}{41868000} \times \frac{BR_{max,cde}}{100}\right) + ES(natural\ gas)_c + ES(nuclear)_c + ES(hydro)_c + ES(wind, solar, etc.)_c \\ + \left(\frac{JFC_c \times 10^6}{JD \times 100} \times BR_{max,cde} - CBP_c\right) \times \frac{BEC \times BD}{41868000} + ES(biofuels\ and\ waste)_c \end{gathered}\right]^2}$$

Differentiate $HHI\_PrP_{min,cde}$ with respect to $BR_{max,cde}$,

$$\frac{d}{d[BR_{max,cde}]}\left(HHI\_PrP_{min,cde}\right) = 2 \times \frac{\begin{gathered} \left[\begin{gathered}\left(\frac{JFC_c \times 10^6}{JD \times 100} \times BR_{max,cde} - CBP_c\right) \times \frac{BEC \times BD}{41868000} - \left(\frac{JFCE_c}{41868000} \times \frac{BR_{max,cde}}{100}\right) + ES(biofuels\ and\ waste)_c + ES(oil\ products)_c + ES(coal)_c + \\ ES(crude\ oil)_c + ES(natural\ gas)_c + ES(nuclear)_c + ES(hydro)_c + ES(wind, solar, etc.)_c\end{gathered}\right] \times \\ \left[\begin{gathered}\left(\frac{JFC_c \times 10^6}{JD \times 100} \times \frac{BEC \times BD}{41868000}\right) \times \left(\left(\frac{JFC_c \times 10^6}{JD \times 100} \times BR_{max,cde} - CBP_c\right) \times \frac{BEC \times BD}{41868000} + ES(biofuels\ and\ waste)_c\right) - \left(\frac{JFCE_c}{41868000} \times \frac{1}{100}\right) \\ \left(ES(oil\ products)_c - \left(\frac{JFCE_c}{41868000} \times \frac{BR_{max,cde}}{100}\right)\right)\end{gathered}\right] - \\ \left(\left(\frac{JFC_c \times 10^6}{JD \times 100} \times \frac{BEC \times BD}{41868000}\right) - \frac{JFCE_c}{41868000} \times \frac{1}{100}\right)\left[\begin{gathered}\left(\left(\frac{JFC_c \times 10^6}{JD \times 100} \times BR_{max,cde} - CBP_c\right) \times \frac{BEC \times BD}{41868000} + ES(biofuels\ and\ waste)_c\right)^2 + \\ \left(ES(oil\ products)_c - \left(\frac{JFCE_c}{41868000} \times \frac{BR_{max,cde}}{100}\right)\right)^2 + ES(coal)_c^{\,2} + ES(crude\ oil)_c^{\,2} + \\ ES(natural\ gas)_c^{\,2} + ES(nuclear)_c^{\,2} + ES(hydro)_c^{\,2} + ES(wind, solar, etc.)_c^{\,2}\end{gathered}\right]\end{gathered}}{\left[\begin{gathered}\left(\frac{JFC_c \times 10^6}{JD \times 100} \times BR_{max,cde} - CBP_c\right) \times \frac{BEC \times BD}{41868000} - \left(\frac{JFCE_c}{41868000} \times \frac{BR_{max,cde}}{100}\right) + \\ ES(biofuels\ and\ waste)_c + ES(oil\ products)_c + ES(coal)_c + ES(crude\ oil)_c + ES(natural\ gas)_c + ES(nuclear)_c + ES(hydro)_c + ES(wind, solar, etc.)_c\end{gathered}\right]^3}$$

Maximum blending ratio will occur when:

$$\frac{d}{d[BR_{max,cde}]}\left(HHI\_PrP_{min,cde}\right) = 0$$

</td></tr>
<tr><td>2</td><td>

Maximum Blending Ratio [$BR_{max,cde}$]

$$\therefore BR_{max,cde} = \frac{\begin{gathered} -\left[\frac{JFCE_c}{41868000 \times 100}\right]\left[\left(\frac{BEC \times BD}{41868000}\right)(CBP_c) - (ES(biofuels\ and\ waste)_c)\right]^2 + \left[\frac{JFCE_c}{41868000 \times 100}\right]\left[\begin{gathered}\left(\begin{gathered}ES(coal)_c + ES(crude\ oil)_c + ES(natural\ gas)_c + ES(nuclear)_c \\ +ES(hydro)_b + ES(wind, solar, etc.)_c\end{gathered}\right)(ES(oil\ products)_c) \\ -\left(\begin{gathered}ES(coal)_c^{\,2} + ES(crude\ oil)_c^{\,2} + ES(natural\ gas)_c^{\,2} + ES(nuclear)_c^{\,2} \\ +ES(hydro)_c^{\,2} + ES(wind, solar, etc.)_c^{\,2}\end{gathered}\right)\end{gathered}\right] + \\ \left[\left(\frac{BEC \times BD}{41868000}\right)(CBP_c) - (ES(biofuels\ and\ waste)_c)\right]\left[\begin{gathered}\left(\frac{JFC_c \times 10^6}{JD \times 100}\right)\left(\frac{BEC \times BD}{41868000}\right)\left(\begin{gathered}ES(coal)_c + ES(crude\ oil)_c + ES(natural\ gas)_c + ES(nuclear)_c + ES(hydro)_c + \\ ES(wind, solar, etc.)_c + ES(oil\ products)_c\end{gathered}\right) \\ -(ES(oil\ products)_c)\left(\frac{JFCE_c}{41868000 \times 100}\right)\end{gathered}\right] \\ + \left[\left(\frac{JFC_c \times 10^6}{JD \times 100}\right)\left(\frac{BEC \times BD}{41868000}\right)\right]\left[\left(ES(coal)_c^{\,2} + ES(crude\ oil)_c^{\,2} + ES(natural\ gas)_c^{\,2} + ES(nuclear)_c^{\,2} + ES(hydro)_c^{\,2} + ES(wind, solar, etc.)_c^{\,2}\right) + (ES(oil\ products)_c)^2\right]\end{gathered}}{\begin{gathered}\left[\left(\frac{JFCE_c}{41868000 \times 100}\right)^2\right]\left[\begin{gathered}(ES(coal)_c + ES(crude\ oil)_c + ES(natural\ gas)_c + ES(nuclear)_c + ES(hydro)_c + ES(wind, solar, etc.)_c) - \\ \left(\left(\frac{BEC \times BD}{41868000}\right)(CBP_c) - (ES(biofuels\ and\ waste)_c)\right)\end{gathered}\right] + \\ \left[\left(\frac{JFCE_c}{41868000 \times 100}\right)\left(\frac{JFC_c \times 10^6}{JD \times 100}\right)\left(\frac{BEC \times BD}{41868000}\right)\right]\left[(ES(oil\ products)_c) - \left(\left(\frac{BEC \times BD}{41868000}\right)(CBP_c) - (ES(biofuels\ and\ waste)_c)\right)\right] + \\ \left[\left(\frac{JFC_c \times 10^6}{JD \times 100}\right)^2\left(\frac{BEC \times BD}{41868000}\right)^2\right][ES(coal)_c + ES(crude\ oil)_c + ES(natural\ gas)_c + ES(nuclear)_c + ES(hydro)_c + ES(wind, solar, etc.)_c + ES(oil\ products)_c]\end{gathered}}$$

</td></tr>
</table>

Supplementary Table 9: Food Stress vs. Blending Ratio (FS vs. BR)

<table>
<tr><th>Steps</th><th>Description</th></tr>
<tr><td>Concept</td><td>For blending ratio, x in range of 1 to 50 %, multiplied the blending ratio with current "Jet Fuel Consumption (JFC_c)" for each country to obtain the "Bio-Jet Fuel Production at the blending ratio (xBP_c)"

$$xBP_c = \frac{x}{100} \times \frac{(JFC_c \times 10^6)}{JD}$$</td></tr>
<tr><td>Concept</td><td>Calculate the energy content in "Bio-Jet Fuel Production at the blending ratio (xBP_c)", $xBPE_c$

$$xBPE_c = xBP_c \times BEC \times BD$$</td></tr>
<tr><td>Concept</td><td>Calculate the Calories Deficit Limit (CDL_c) in each country,

$$CDL_c = Food\ Limit \times (Population_c \times 1000) \times 365 \times \frac{41868000}{1 \times 10^{10}}$$
NOTE:

Unit of Food Limit = kcal/capita/day
Assuming 365 days per year
Conversion from kilocalories to megajoules = 0.0041868</td></tr>
<tr><td>Concept</td><td>Check:
If, $xBPE_c$ < TDC_c;

Then, repeat step 1-4 by increasing blending ratio, x by 1%;

Else;
Then, limiting factor = "Food";
Maximum blending ratio = x-1;</td></tr>
<tr><td>1</td><td>Calories Deficit Limit [CDL_c]

$$CDL_c = Food\ Limit \times (Population_c \times 1000) \times 365 \times \frac{41868000}{1 \times 10^{10}}$$
NOTE:

Unit of Food Limit = kcal/capita/day. Assuming 365 days per year. Conversion from kilocalories to megajoules = 0.0041868.</td></tr>
<tr><td>2</td><td>Derivation of Maximum Blending Ratio [x_{max}]

Due to the limitation in terms of the maximum number of rows (1048576) in Microsoft Excel, generating a huge pivot table will be very inconvenient, thus working backwards is the best solution

Maximum blending ratio occurs when:

$$x_{max}BPE_{cde} = CDL_c,$$
$$x_{max}BP_{cde} \times BEC \times BD = CDL_c,$$
$$\frac{x_{max}}{100} \times \frac{(JFC_c \times 10^6)}{JD} \times BEC \times BD = CDL_c.$$</td></tr>
<tr><td>3</td><td>Maximum Blending Ratio [x_{max}]

$$\therefore x_{max} = \frac{CDL_c \times 100 \times JD}{BEC \times BD \times (JFC_c \times 10^6)}.$$</td></tr>
</table>

Supplementary Table 10: Feedstock Quantity vs. Blending Ratio (FQ vs. BR)

Steps	Description
**1**	When the limiting factor of a country is "Crude Oil Price", the maximum blending ratio (x_{max}) will be equal to Total Blending Ratio _ Profitable Potential (TBR_PrP_{cde}).
**2**	If the maximum blending ratio of Crude Oil Price is equal to the Total Blending Ratio _ Potential (TBR_P_c), then the limiting factor for a country will be its "Feedstock Quantity", otherwise the limiting factor for that country remains as "Crude Oil Price".<br><br>$Provided\ that\ Limiting\ Factor_c\ =\ "Crude\ Oil\ Price",$<br>$if\ x_{max} = TBR\_PrP_{cde} = TBR\_P_c,$<br>$then\ Limiting\ Factor_c\ =\ "Feedstock\ Quantity",$<br>$else\ Limiting\ Factor_c\ =\ "Crude\ Oil\ Price".$

Supplementary Table 11: Summary of the limiting factor calculation

Steps	Description
1	**Comparing Crude Oil Price, Water Stress, Herfindahl-Hirschman Index and Food Stress.**<br><br>Compare the blending ratios for these possible limiting factors for every country.<br><br>When one of the possible limiting factors has the lowest blending ratio, then it is the limiting factor of a particular country. Its corresponding blending ratio (x %) will be the maximum blending ratio of that country.<br><br>The maximum blending ratio allowed by each limiting factor (Water Stress, Food Stress, Herfindahl-Hirschman Index, Crude Oil Price) are compared equally without any sequences, the limiting factor that allows the lowest blending ratio among these 4 limiting factors is chosen to be the final/main limiting factor.
2	**Comparing Feedstock Quantity with Crude Oil Price.**<br><br>Determine whether the blending ratio for a country is Feedstock Quantity when the blending ratio (after step 1) is Crude Oil Price.<br><br>Refer to Supplementary Table 10 Feedstock Quantity vs. Blending Ratio (FQ vs. BR) for more details.

9. Indexing

$$Let\ calculated\ data\ to\ be\ denoted\ as\ x_n, 1 \leq n \leq 155.$$

1) Min-Max Normalisation

$$\frac{x_n - \min(x_1, x_2, x_3, \dots \dots, x_{153}, x_{154}, x_{155})}{\max(x_1, x_2, x_3, \dots \dots, x_{153}, x_{154}, x_{155}) - \min(x_1, x_2, x_3, \dots \dots, x_{153}, x_{154}, x_{155})} \times 100\%$$

2) Reversed Min-Max Normalisation

$$100\% - \frac{x_n - \min(x_1, x_2, x_3, \dots \dots, x_{153}, x_{154}, x_{155})}{\max(x_1, x_2, x_3, \dots \dots, x_{153}, x_{154}, x_{155}) - \min(x_1, x_2, x_3, \dots \dots, x_{153}, x_{154}, x_{155})} \times 100\%$$

3) Max Normalisation

$$\frac{x_n}{\max(x)} \times 100\%,$$
$$e.g. \max(Number\ of\ Available\ Feedstock) = 39.$$

4) Ranking

$$If\ x_n = 0,$$
$$0,$$
$$else\ SUMPRODUCT\left(\frac{x_n < [x_1 \cdots \quad x_n \quad \cdots x_{155}]}{COUNTIF([x_1 \cdots \quad x_n \quad \cdots x_{155}], [x_1 \cdots \quad x_n \quad \cdots x_{155}])}\right) + 1.$$

5) Percentage

$$x_n$$
