# Supplementary material for: Limitations to sustainable renewable jet fuels production attributed to cost than energy-water-food resource availability
Source: Nat Commun. 2023 Dec 9;14:8156. doi: 10.1038/s41467-023-44049-6 (PMC10710432; doi:10.1038/s41467-023-44049-6)
Supplement: Supplementary file 3 — Description of additional supplementary files [file 41467_2023_44049_MOESM3_ESM.pdf]

Description of additional supplementary files

Supplementary Dataset 1- Global Sustainability Index Ranking

Supplementary Dataset 2- Feedstock

Supplementary Dataset 3- Economic Scenarios

Supplementary Dataset 4- EWF Index Indicators

Supplementary Dataset 5- Dataset List and Source

Supplementary Dataset 6- Figure Data
